# Underlying export characteristics and projected country positions in the agri-food trade: A global system analysis

**DOI:** 10.1038/s41538-026-00944-y

**Published:** 2026-06-26

**Authors:** Amirhosein Ghozatfar, Tina Sardashti, Deniz Berfin Karakoc

**Affiliations:** 1 Department of Management Science, University of Strathclyde, Glasgow, UK; 2 STOR-i Centre for Doctoral Training, Lancaster University, Lancaster, UK; 3 School of Computing and Augmented Intelligence, Arizona State University, Tempe, AZ USA

**Keywords:** Economics, Economics, Environmental social sciences, Politics and international relations

## Abstract

The global agriculture and food trade plays a critical role in ensuring food security and economic stability. A country’s agri-food export share is contingent upon various social, economic, and physical factors, and it defines its position within the global trade system. In this study, we propose a machine learning framework to systematically identify the most informative characteristics associated with export shares of countries and explore their projected positions within the agri-food trade. Our study focuses on 25 countries with varying export shares spanning from 1988 to 2022. The results show that the performance of logistics systems, population, reliability of government, and value of agricultural production have the greatest impact on shaping the export share of a country. Further, by 2035, the United States, the Netherlands, Brazil, Germany, China, and Australia are projected to be the leading exporters of global agriculture and food trade, following current trends closely. Our findings can offer insights to policymakers and researchers, enabling more strategic decision-making.

## Introduction

The global agriculture and food trade is a major contributor to the economic growth of many countries^1^, particularly for the ones that have a comparative advantage of supplying these essential goods through their expansive exports^2^. Substantial contributions to agri-food trade feed countries' national agricultural sector, which is a critical source of employment^3^ that alleviates poverty^4^. It also initiates large-scale infrastructure investments^5^, open international relations^6^, and advanced technological developments across relevant sectors^7^ which add value to a country’s position within the global scene. Yet, the relationship between these improvements and export shares of a country is bilateral. Thus, it is important to better understand the underlying export characteristics of countries and more accurately project their future positions at the global scale, as agri-food trade structure provides a foundational opportunity for mitigating economic shocks countries might experience^8^.

Complementary to economic stability, exports of agri-food commodities ensure global food security by bridging supply with demand in distant locations^9^. Countries that face limited domestic supply capacity rely on the trade system as their main food source to meet daily energy and nutrition intake needs^10^. Food security of these countries with high dependence on supply of agri-food commodities from abroad is highly vulnerable to downstream impacts of risks that originate in their suppliers^11^ as evidence shows that supply chain disruptions, pandemics, labor shortages, and trade restrictions within the global trade system lead to food price inflation and reduced access to essential goods^12^. Given the increasing projections of food-insecure population by 2030 worldwide^13^, revealing the underlying characteristics of successful agri-food exports is urgently needed as it will ensure progress towards UN Sustainable Development Goal 2: Zero Hunger^14^.

Over the years, researchers have pinpointed several key factors, such as economic policies and development, that could boost a country’s agricultural growth and potentially contribute to its export share^15,16^. They have concluded that reducing barriers such as tariffs and quotas has facilitated greater trade flows between European countries^17^. Similarly, tariff reductions and infrastructure developments have led to a considerable expansion of EU–China bilateral agricultural trade^18^. Further, a positive association between regional trade agreements, agricultural trade in Africa^19^, and agricultural trade connectivity around the globe has been discussed in detail^20^. In addition, key drivers of forming global food trade communities^21^, as well as influential economic, historical, and structural factors of agri-food exports from Italy to non-EU Mediterranean countries^22^ have been introduced. Likewise, upgrading to higher-value products - from raw commodities to processed goods - has appeared as a supportive factor in exports^23^. Following the identification of key factors, separate studies have proposed long-term forecasts of Russia’s agri-food export gains^24^, Nigeria’s agri-food exports to 70 major trading partners^25^, and global rice market growth^26^. A relevant and substantial literature has studied the global agri-food trade structure^27,28^ and how its diversity contributes to resilience^29,30^, food access^31^ and nutrition security^32^.

While existing literature unravels important information for agri-food exports, most analyses are restricted to a single country, region, commodity, or supportive factor, which limits a systematic comparison of the relative importance across a broad set of informative characteristics between exporters. Thus, a global scale examination of the diverse social, economic, and physical characteristics that explain the agri-food export share of countries is still missing. Subsequently, systematically understanding the historical patterns within these informative characteristics to project the future position of exporters within the global agri-food trade has not been addressed yet. Our study fills this important gap by developing a machine learning framework to comprehensively evaluate the historical and projected positioning of 25 countries with varying agri-food export shares between 1988 and 2022. Providing such system-level, descriptive, and projection-based insights into agri-food trade dynamics can support more informed research, policy, and decision-making.

Here, we first examine which social, economic, and physical characteristics, and to what extent, can be informative to explain agri-food export share patterns across countries. Building on this, we explore country export-position projections toward 2035. Lastly, we assess the concentration of their agri-food supplies to discuss potential risks against their positions within the global system. The research questions guiding this study are: (1) Which social, economic, and physical characteristics are informative to explain agri-food export shares? (2) How is exporter positioning in agri-food trade projected to evolve toward 2035? (3) How concentrated are agri-food supplies of these leading exporters globally?

## Results

In this section, we address each research question in detail and discuss the importance of our findings under food security and the economic stability of countries. We also evaluate the advantages, limitations, and future work directions of our study.

### Patterns and informative characteristics of agri-food exports

**Fig. 1:**
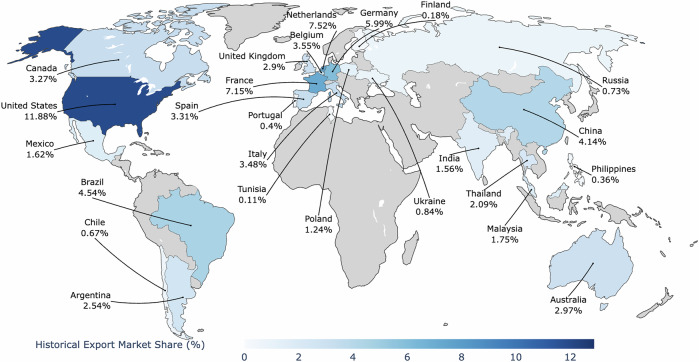
Average export shares of 25 countries between 1988 and 2022. Darker shades indicate larger export shares, whereas lighter shades indicate lower export shares. These 25 countries have complete data historically. Gray-shaded areas are out of scope for this study.

An analysis of historical agri-food export data from 1988 to 2022 identifies several structurally prominent countries across a wide range of geographies and market share profiles. Figure 1 illustrates the 25 countries of interest with their corresponding average agri-food export share between 1988 and 2022, which collectively cover over 75% of the global agri-food market.

**Fig. 2:**
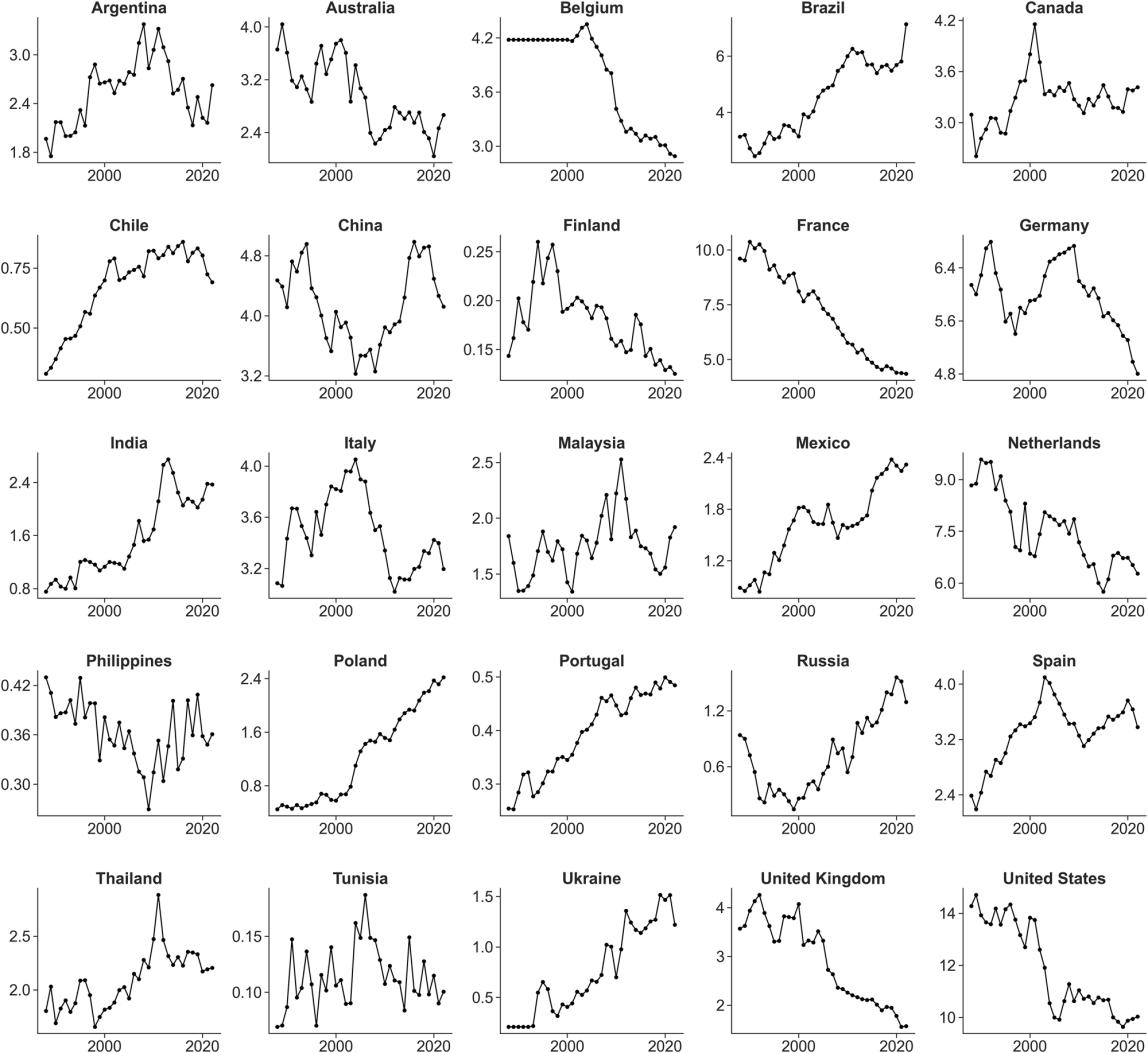
Historical trends in agri-food export shares of 25 countries from 1988 to 2022. Unit of per exporter share is a percentage, and collectively they cover over 75% of the entire market.

Over time, Fig. 2 reveals broadly smooth, long-run trends for agri-food export share across many countries. In general, emerging markets and developing economies exhibit long-term increases in their share of exports, whereas mature economies tend to show volatile or gradually declining shares. During the years of the COVID-19 downturn, most export shares either leveled off or dipped instead of following their prior increasing trends, which is also corroborated by the United Nations Trade & Development Reports^33^.

More specifically, the United States shows a decreasing trend, as well as European countries such as the United Kingdom, France, Germany, Belgium, and the Netherlands experience decreases in their global agri-food export shares over time. This can be explained by the changing composition of the products traded internationally and declining relative productivity in the early 2000s^34^. Further, it can be attributed to intensified global competition and market shifts, in which emerging exporters, notably BRIC countries (Brazil, Russia, India, and China), and trade liberalization have pressured these traditional exporters^35,36^. Supportively, Brazil, Argentina, Chile, India, Mexico, Poland, Portugal, Russia, Spain, Thailand, and Ukraine indicate increasing trends in their export shares. This can be generally linked to rapidly growing food demand and production in their domestic markets^37^ as the Food and Agriculture Organization of the United Nations (FAO) highlights that population growth, urbanization, and rising incomes in developing countries have spurred stronger food demand and output^38^ (please refer to Table S12 in the SI for the statistical tests for time trends of agri-food export share per country).

Further, historical data underscores the long-standing dominance of a few key countries in spite of fluctuations and recent decreases. For instance, the United States is continuously the foremost power, commanding an average of 11.88% of the market share, ranking it first both globally and within this study’s cohort. Several European countries demonstrate the continent’s significant role in agri-food trade. The Netherlands, France, and Germany historically hold the second, third, and fourth positions, respectively, with average export shares between 6% and 7%. Beyond Brazil, China, and Canada are other significant agri-food exporters, all of whom continuously maintain top-ten rankings (please refer to Table S8 in SI for the correspondence between global vs. cohort ranking of these countries according to their average export market shares). The global rankings confirm the representative and diverse nature of the selected countries for analyzing the dynamics of the global agri-food export market, which also ensures a comprehensive picture.

Our analysis also reveals the key characteristics of countries for explaining their agri-food export share patterns. Table 1 summarizes these informative characteristics with their extent of association with agri-food export shares. The first tier of informative characteristics—relatively higher degree of impact—includes ‘Logistics Performance Index’; ‘Population’; ‘Government Reliability Index’; and ‘Total Value of Crop, Animal, and Aquaculture Production’. The second tier—relatively moderate degree of impact—includes ‘GDP’; ‘Infrastructure Investment and Maintenance in Transport’; and ‘Share of Food, Beverage, and Tobacco Subsector’s Value Added within the Total Manufacturing Sector’. The last tier—relatively lower degree of impact—includes ‘Number of Trade Agreements’ as the explanatory factors of varying agri-food export shares among the studied 25 nations. However, ‘Disaster Days within a Year’, ‘Geopolitical Risk Index’, and ‘Agriculture, Forestry, and Fishing Annual Value Added (% Growth)’ provide little to no information on agri-food export share patterns; thus, their impact is not considered statistically significant (Please refer to text and Tables S9–S11 in SI for a more detailed explanation). Our findings on the informative characteristics of export market share patterns are supported by the previous literature^39,40^. Even though these studies focus on individual factors or select countries^41,42^, they still give us confidence in our novel, systematic, and comprehensive findings.

**Table 1:** Informative characteristics for explaining agri-food export share patterns over the last three decades across 25 countries collectively

Informative characteristics	Degree of association
Logistics performance index	1.36
Population	1.19
Government reliability index	1.17
Total value of crop, animal, and aquaculture production	1.07
GDP	0.85
Infrastructure investment and maintenance in transport	0.74
Share of food, beverage, and tobacco subsector’s value added within the total manufacturing sector	0.64
Number of trade agreements	0.41

The mutual information coefficient quantifies each characteristic’s statistical association with export shares, where larger values indicate greater shared information across observed patterns.

### Country-specific agri-food export positioning projections

**Fig. 3:**
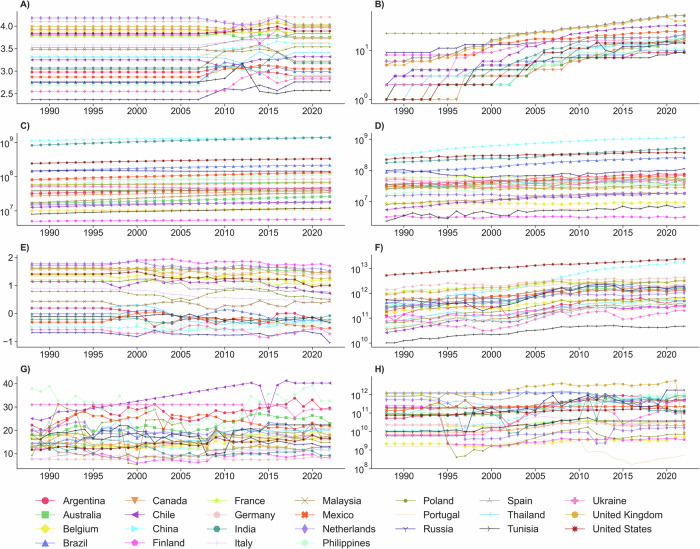
Historical trends for informative characteristics of 25 countries from 1988 to 2022. **A** Logistics performance index, **B** number of trade agreements, **C** population, **D** total value of crop, animal, and aquaculture production [thousand $ USD], **E** government reliability index, **F** GDP [$ USD], **G** share of food, beverage, and tobacco subsector’s value added within the total manufacturing sector [percentage], **H** infrastructure investment and maintenance in transport [$ USD]. For a clearer visualization, **C**–**D-F-H** are plotted on a log scale.

In Table 2, 2035 forecasts of informative characteristics of agri-food export shares—under baseline conditions—are presented. Given their historical trends in Fig. 3, some of these characteristics provide anticipated projections as they have more stable increases. Conversely, some informative characteristics experience more fluctuations between 1988 and 2022 among 25 countries which lead to a higher variation in the projected values (please refer to Table S13–S37 in the SI for the statistical test of time trends for each explanatory factor per country). The estimated future trends of informative characteristics are the foundation of our 2035 projections for exporter-specific positioning in the agri-food trade system.

**Table 2:** 2035 forecasts for informative characteristics of agri-food export share patterns by country

	Logistics performance index (LPI)	Number of trade agreements	Population	Total value of crop, animal, and aquaculture production	Government reliability index	GDP	Share of food, beverage, and tobacco subsector’s value added within the total manufacturing sector	Infrastructure maintenance and investment in transport
Argentina	2.91	16	5.00E + 07	8.95E + 07	−0.25	5.12E+11	29.73	4.28E+11
Australia	3.70	34	3.02E + 07	5.71E + 07	1.42	1.67E+12	25.16	1.44E+12
Belgium	4.00	78	1.60E + 07	1.11E + 07	1.01	7.28E+11	16.90	9.02E+09
Brazil	3.01	15	2.48E + 08	3.21E + 08	−0.24	1.64E+12	17.00	6.13E+11
Canada	3.69	20	4.20E + 07	5.78E + 07	1.42	2.23E+12	19.24	2.80E+10
Chile	3.44	39	2.25E + 07	2.07E + 07	0.68	3.69E+11	39.47	7.02E+11
Germany	4.24	78	8.56E + 07	5.12E + 07	1.28	4.31E+12	7.80	3.89E+10
Finland	4.09	78	9.76E + 06	4.57E + 06	1.62	3.38E+11	11.24	7.67E+09
France	3.79	78	7.04E + 07	5.69E + 07	0.96	3.13E+12	20.28	4.05E+10
India	3.24	27	1.49E + 09	6.76E + 08	0.29	4.70E+12	10.05	3.15E+11
Italy	3.77	78	6.36E + 07	3.97E + 07	0.59	2.32E+12	11.47	4.51E+10
Malaysia	3.30	22	3.78E + 07	2.04E + 07	0.48	4.35E+11	13.07	5.74E+11
Mexico	3.08	30	1.36E + 08	8.60E + 07	−0.20	1.36E+12	21.29	2.25E+11
Netherlands	3.98	78	2.17E + 07	2.03E + 07	1.47	1.27E+12	19.65	2.12E+10
China	3.73	24	1.48E + 09	1.31E + 09	−0.30	2.76E+13	12.31	1.08E+12
Philippines	2.94	13	1.34E + 08	3.65E + 07	−0.07	5.17E+11	37.40	2.13E+12
Poland	3.68	78	4.22E + 07	3.92E + 07	0.75	8.95E+11	17.27	1.13E+10
Portugal	3.69	78	1.44E + 07	8.73E + 06	0.95	2.98E+11	17.77	3.96E+09
Russia	2.70	20	1.43E + 08	1.62E + 08	−0.94	1.86E+12	16.30	8.60E+11
Spain	4.03	78	5.04E + 07	5.31E + 07	0.79	1.61E+12	21.04	1.46E+12
Thailand	3.42	19	7.19E + 07	5.41E + 07	−0.09	6.61E+11	21.18	9.80E+11
Tunisia	2.69	13	1.61E + 07	8.66E + 06	−0.10	8.68E+10	24.36	4.46E+10
Ukraine	2.94	26	4.19E + 07	4.48E + 07	−0.72	2.61E+11	23.81	2.89E+11
United Kingdom	3.93	43	7.04E + 07	3.02E + 07	1.18	3.64E+12	18.95	3.78E+12
United States	3.84	15	3.63E + 08	3.61E + 08	0.93	3.07E+13	17.73	1.86E+11

The countries are listed in alphabetical order. 2035 projections for each characteristic per country are computed individually.

For instance, ‘GDP’ is a key characteristic where 2035 projections match with anticipated increases based on historical trends (see Fig. S3 in SI). Particularly for the United States and China, our forecasts highlight substantial increases. These predictions suggest that these countries will remain in pivotal positions within the global trade system^43^. Given its steady upwards trend, our projections for ‘Population’ also lead to anticipated increases in 2035 for every country, particularly for China and India (see Fig. S8 in SI). A significant increase in ‘Number of Trade Agreements’ is projected for European countries (see Fig. S7 in SI) based on the continuum of trade initiatives, such as open borders and regional agreements^44^. ‘Infrastructure Investment and Maintenance in Transport’ is another informative characteristic with generally increasing trends through time (see Fig. S5 in SI). Except for Finland, Mexico, the Netherlands, and France, which seem to stabilize their spending, the rest of the projections for 2035 are on the higher end, which are in line with recent government initiatives on increasing funding for transportation projects in Europe^45^, Asia^46^, Australia^47^, as well as South and North America^48,49^.

‘Logistics Performance Index’ exhibits slight fluctuations over the historical period, leading to minor decreases or increases in projections among countries (see Fig. S6 in SI). For instance, Australia, Belgium, Canada, France, the Netherlands, the United Kingdom, and the United States are projected to experience relatively small reductions, which can be explained by the inherent difficulty of continuous optimization at already high levels^50^. Also, Russia is projected to maintain its declining trend, which can be supported by the shortage of skilled workers and persistent challenges in deploying, maintaining, and securing digital tools^51^. On the contrary, ‘Government Reliability Index’ generates high fluctuations within its projections (see Fig. S4 in SI). In 2035, Finland, Germany, Australia, Canada, and the Netherlands are estimated to experience slight decreases in their high reliability indices, whereas Russia, India, Mexico, and China are projected to experience slight increases in their low reliability indices. This can be explained by lower-governance countries gaining small momentum as reforms take hold, whereas higher-governance countries often see relatively small gains as they are already committed to continuous reforms^52^.

In addition, ‘Share of food, beverage, and tobacco subsector’s value added within the total manufacturing sector’ highlights fluctuations through time for each country (see Fig. S6 in SI); thus, 2035 projections vary highly. Generally, Brazil, Chile, Germany, Mexico, and Ukraine are predicted to experience slight decreases, which can be due to the growth of other manufacturing sub-sectors. Lastly, ‘Total value of crop, animal, and aquaculture production’ has almost a steady increase for each country between 1988 and 2022 (see Fig. S9 in SI), resulting in the same trends for future predictions where China, India, the United States, and Brazil are projected to become some of the main dominating global agri-food producers in 2035^53,54^.

**Fig. 4:**
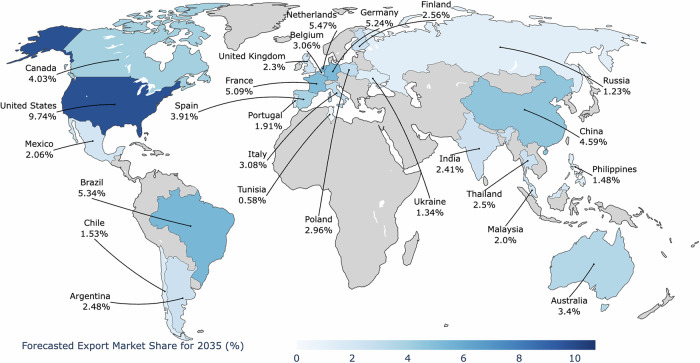
Export share projections for 25 countries in 2035. Darker shades indicate higher export shares, whereas lighter shades indicate lower export shares. The unit is percentage, and collectively these 25 countries cover over 75% of the entire market.

Following the 2035 projections of informative characteristics, we also predict the future agri-food ‘Export shares’ as seen in Fig. 4—again under baseline conditions. Our projections reveal an agri-food trade landscape characterized by enduring dominance and significant shifts in export positioning. The 2035 projections suggest a continued concentration of agricultural exports in the United States, the Netherlands, Germany, and France (see Table S38 in SI). This grouping of high-export countries mirrors current trade patterns^55^.

Some of the projected changes in leading exporter positions include the ascent of Brazil. This is reflected through Brazil’s predicted increases in the agriculture and logistics sector, as well as trade agreements^56^. In addition, Canada and Australia are projected to elevate their positions in the market by increasing their export shares in 2035, with the increasing trends in their agri-food sector, GDP, and logistics sector^57,58^. Moreover, ASEAN countries of this study (Malaysia, Philippines, and Thailand), as well as China, India, and Tunisia, are projected to show positive growth in every single informative characteristic, leading their export shares to expand in 2035^59,60^. Lastly, European countries remain a critical hub for agri-food trade in our projections, supported by the boost and sustaining strong performance in almost all of their informative characteristics, which are supported by the existing literature on the agri-food export dynamics of European countries^61,62^.

On the contrary, the United Kingdom, Russia, and Ukraine are projected to have almost stable or declining export shares in 2035, leading to a gradual erosion of their strategic advantages. For the United Kingdom, this projection could be a potential reflection of post-Brexit trade frictions^63^. Similarly, declining Russia and Ukraine projections may be a reflection of regional instability and geopolitical disruptions on their export capabilities^64,65^. For Argentina and the United States, a relatively stable trend through 2035 is expected, potentially influenced by the emergence of new strong competitors (please refer to SI for a more detailed discussion).

This work is among the first to offer projections for leading exporter positions in the agri-food trade under baseline conditions, which are the United States, Brazil, the EU, Canada, and Australia. Nevertheless, our conclusions corroborate the body of literature and government reports that focus on local trends in individual suppliers^66,67^. Our projections point towards a more intercontinental agri-food system where the export leaders will be extended more homogeneously across global regions (Please refer to text and Table S39 in SI for a sensitivity analysis of our future country positioning projections).

### Regional supply concentrations

**Fig. 5:**
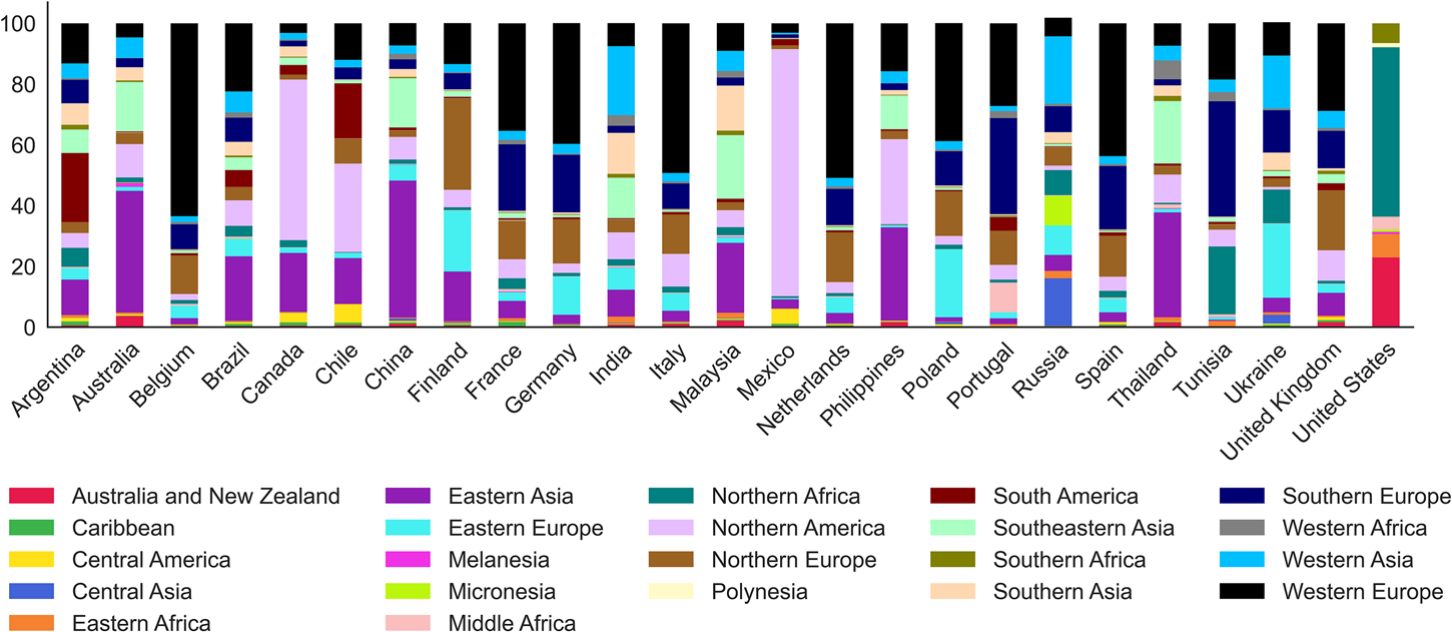
Historical average regional export share [%] for 25 countries. The figure illustrates, on average, what percent of each country’s exports are allocated to various importer regions over the period 1988–2022. Countries with higher export shares to importers in a single region have high supply concentration—low export diversity, whereas nations with low export shares to importers in many regions have low supply concentration—high export diversity.

In Fig. 5, we illustrate the percentage of supplies directed across global regions per 25 countries—averaged over time (please see Table S41 in SI for the categorization of global regions based on FAOSTAT records). This gives a comprehensive perspective on how regionally concentrated—or diversified—each country’s supplies are. We observe that these 25 countries show varying supply concentrations. Several of them do not diversify their supplies, whereas others reach a broader set of importing regions (please refer to text and Figs. S11–S35 in SI for historical regional supply concentrations per exporter).

One of the most clear observations is that many of the European exporters have very high supply concentrations, as almost all of their importers are located within parts of Europe. The Netherlands, Italy, Spain, and Belgium all export almost 50% of their total supply to Western Europe, and over 10% of the supplies are dedicated to Northern Europe, which is similar for Germany and France. This finding is understandable given the open borders, trade policies, and geographic proximity within the EU^68^. Further, Canada and Mexico exhibit a high concentration of supplies, as over 50% of their agri-food commodity exports are to the United States. This is a reflection of open trade agreements within North America and the ability to transport agri-food commodities efficiently. Due to the expanding highway and railway systems that connect the entire continent^69^ and high agri-food demand within the United States^70^, Canada, and Mexico, incline towards low export diversification.

On the contrary, many exporters in Asia showcase low regional supply concentrations. Particularly, India, Malaysia, the Philippines, and Russia depict the lowest regional supply concentrations, e.g., the highest export diversification. These countries still provide the majority of their supplies to importers within their continent. Yet, they supply considerable proportions—around 10% each—to other regions, including North America, Europe, and Africa, which complements their export diversity. Such observations could be due to the contributions of staple grains (e.g., rice and soy) within their agricultural production^71^, thus, their strategic trade power over these globally the most consumed agri-food commodities^72^.

**Fig. 6:**
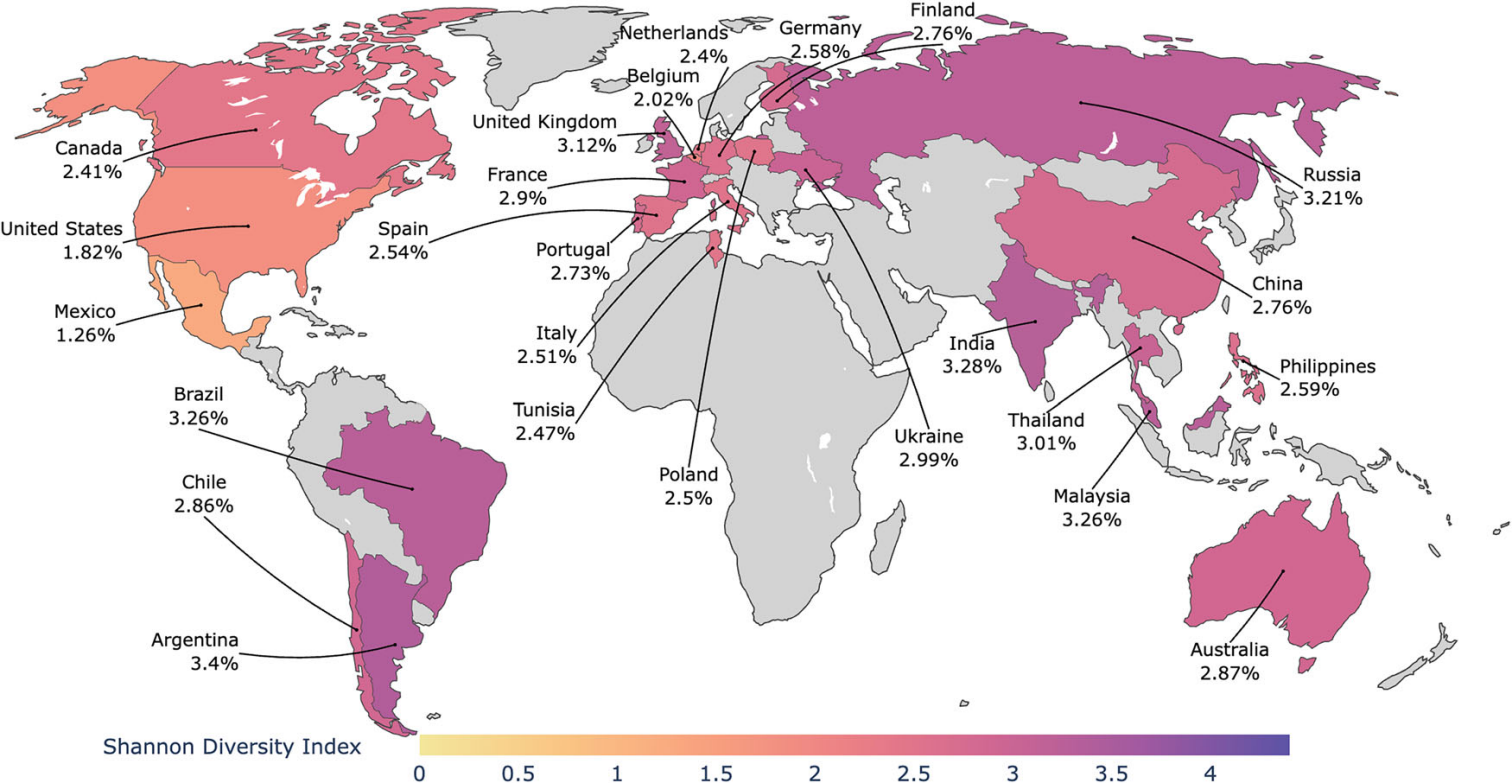
Average Shannon Diversity Index for 25 countries over the period 1988–2022. Darker shades correspond to greater historical diversity in export destinations, whereas lighter shades denote lower diversity.

Lastly, to fairly compare supply concentrations among 25 exporters, we present their Shannon diversity indices (see Table S40 in SI). As seen in Fig. 6, Argentina, India, Malaysia, Brazil, and Russia exhibit relatively higher export diversity. In contrast, France, Australia, Chile, and Finland display moderately concentrated supply patterns while Mexico’s exports are highly concentrated (please see Fig. S10 in the SI for the historical Shannon diversity index of all countries).

These supply concentrations have existed continuously over the last three decades per country, which makes it crucial to reflect on their implications for export dependency in times of local crisis within their importers. As evidence shows, climate change-induced weather events, economic crises, geopolitical disputes, and rapidly changing trade policies in importing nations can drastically impact the competitive position of these exporters. For instance, with such a high concentration of supplies in a single region, export revenues within Europe have decreased heavily due to Brexit^73^, and with the recent tariffs imposed by the United States, Canada and Mexico have faced a challenge in maintaining their trade-led economic well-being^74^. These occurrences highlight how essential it is for countries to diversify their exports and globalize their supplies to enhance their economic growth and trade positions^75^.

## Discussion

This study introduces a machine learning framework to identify informative relationships and underlying patterns within the agri-food exports. Compared to the existing literature that studies local trends within individual countries, our novelty is designing a descriptive and predictive analysis on identifying informative characteristics, projected leading positions, and supply concentrations of agri-food exporters collectively at the global scale. As we analyze a panel dataset that includes social, economic, and physical factors over a three-decade span for 25 countries, our study systematically captures both temporal trends and spatial variations.

We conclude that logistics systems, the agriculture and food sector, economic growth, and the reliability of the government ruling collectively play a central role in explaining the agri-food export share of a country. Our 2035 projections highlight the United States, Germany, the Netherlands, France, and Brazil as projected leading exporters of agri-food commodities, collectively. We also reveal that agri-food exports of many European countries are heavily concentrated in Europe, whereas exports from Canada and Mexico are mainly designated for the United States. In contrast, many exporters in Asia continuously diversify their supplies across regions. These patterns position low-diversity exporters in a more vulnerable state and high-diversity exporters in a less vulnerable state against local shocks within their main importers. Based on our findings, ongoing global efforts can prioritize retraining from geopolitical conflicts, export bans, and sudden tariff policy changes to ensure a well-functioning agri-food trade system.

The primary limitation of this study arises from data availability issues, which constrains the depth and scope of analysis. Indeed, neither all exporter countries nor finer granularity in agri-food commodities are studied due to concerns about missing data. However, our data sources are well-accepted organizational platforms that are constantly being regulated and updated^76^. Thus, our findings have a solid foundation and a significant importance to initiate better-informed trade strategies around the globe. Further, our projections are interpreted as conditional, scenario-based predictions in line with our data analytics study of agricultural and food trade from a system-perspective. The revealed historical associations between export shares and their informative characteristics during 1988–2022 are broadly assumed to remain unchanged. Hence, our projections should be interpreted as indicative of the prospective positioning of leading exporters under baseline conditions (please refer to SI for a more detailed discussion).

Given that, future research can build on our approach as it is highly generalizable and broadly applicable. Our data-driven machine learning framework can be extended to a trade system of individual agri-food commodities across all the remaining exporters at various spatial scales. Such studies can incorporate heterogeneous market structures and distinct explanatory variables per commodity. Further, studies with a more econometrics focus can include impacts of exchange-rate movements, commodity price cycles, and supply disruptions across competing exporters to drive more dynamic forecasts, as well as they can simulate structural breaks arising from geopolitical events, climate shocks, policy regime shifts, or transformations in food systems to assess their impacts on leading exporter positioning within the global scene.

## Methods

We create a panel data set of country-specific agri-food export shares spanning over 30 years and potential informative characteristics of explaining export patterns. This is a heavily data-driven framework; thus, the availability of complete data defines the study scope in terms of time period (1988–2022) and exporters (a total of 25 countries across the globe with varying export shares of agri-food commodities) that are evaluated. Yet, we collectively cover 75% of the agri-food export market by focusing on these select countries.

**Fig. 7:**
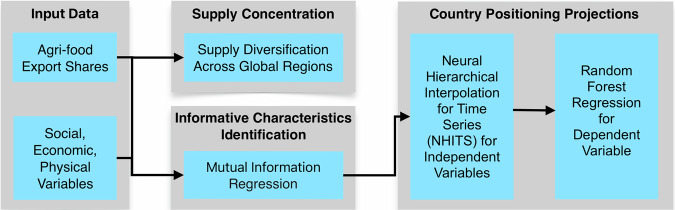
Schematic of our machine learning framework introduced in this study. We compile a panel dataset covering 25 countries with varying export shares of agri-food commodities from 1988 to 2022. We use this dataset to identify the informative characteristics of a country’s agri-food export share, project agri-food export positions in 2035, and reveal their supply concentration to highlight vulnerability against local risks in their importers.

We develop a machine learning framework to better understand the export-driven agri-food trade system dynamics. We first identify the informative characteristics of agri-food market export shares and then analyze their historical trends to compute 2035 predictions of export shares. We further evaluate the supply concentration of these exporters across global regions. Figure 7 summarizes our machine learning framework. Below, we describe our study design, informative characteristic identification, future projections, and supply concentration procedures in detail.

### Study design

Our machine learning framework is heavily data-driven. We collect empirical data on agri-food exports and their potential informative characteristics at an annual time step between 1988 and 2022 for 25 countries with varying market shares. Here, we consider the entirety of agri-food commodities that are available in the FAOSTAT database to define export market share (see Table S1 in SI). This is a deliberate design choice to provide a more comprehensive and stable understanding of agri-food export patterns, as lower granularity in commodity specifications allows complete data that is available for a higher number of countries in a longer time period (please refer to SI for a more detailed explanation of input data). Further, we adopt *‘export share’* as an outcome-based indicator of a country’s position within the global agri-food trade system, rather than a direct measure of its productivity or comparative advantage. Thus, historical data on country export shares provide a system-level perspective on their positioning^77^.

Table 3 lists the considered social, economic, and physical factors that are assumed to be informative on explaining agri-food export share patterns across countries globally, according to a thorough literature review^2,78,79^.

**Table 3:** A list of input data in this study

Factor’s name	Data source
Export market share [percentage]	Food and Agriculture Organization^94^
GDP [$ USD]	World Bank^95^
Government reliability index	World Bank^81^
Logistics performance index	World Bank^96^
Number of trade agreements	World Bank^97^
Population	U.S. Census Bureau International Database^98^
Share of food, beverage, and tobacco subsector’s value added within the total manufacturing sector [percentage]	World Bank^99^
Infrastructure investment and maintenance in transport [$ USD]	World Bank^100^ Organization for Economic Co-operation and Development^101,102^
Total value of crop, animal, and aquaculture production [thousand $ USD]	USDA Economic Research Service^103^
Disaster days within a year [%]	The International Disaster Database^104^
Geopolitical risk index	American Economic Association^105,106^
Agriculture, forestry, and fishing, annual value added [% growth]	World Bank^107^

All data is collected individually for each exporter annually between 1988 and 2022. Export market share is the independent variable, whereas the rest are its potential informative characteristic in our machine learning framework. These datasets are complete for 25 countries within the given time span, which defines the scope of our study.

‘Logistics performance index’ reflects the robustness of the infrastructure, efficiency of customs, and quality of logistics services that facilitate value chain management. Lower values give insights into the existence of logistical challenges (e.g., inefficient ports, burdensome customs procedures, or inadequate transport and storage systems), which can act as a significant impediment to country-specific export activities^80^. Similarly, ‘government reliability index’ indicates how stable, predictable, and transparent the governmental policy environment is^81^, where positive values can be conducive to fostering trade, attracting investment, and ensuring confidence to international stakeholders^81^. A larger ‘Population’ can contribute to a higher labor availability in the context of trade^82^, a higher ‘GDP’ reflects a country’s economic size and productive capacity to boost trade, and a higher ‘Number of Trade Agreements’ suggests open trade policies, thus, favorable export conditions. Further, ‘Infrastructure Investment and Maintenance in Transport’ represents government spending on building and maintaining all modes of transport and related logistics networks. As well-performing transport infrastructure lowers trade costs, shortens delivery times, and improves market access, it can improve trade activeness^83^. Lastly, ‘total value of crop, animal, and aquaculture production’ highlights a country’s agricultural output, whereas ‘share of food, beverage, and tobacco subsector’s value added within the total manufacturing sector’ reflects the extent of domestic food processing. They can collectively account for the total agri-food commodity supply and its contribution to export capacity^84^ (please refer to SI for a more detailed explanation of each factor and their computations).

### Informative characteristic identification

To examine which social, economic, and physical factors are informative for explaining agri-food export share patterns, we apply mutual information regression to our panel data. Mutual information regression is a machine learning-based feature selection approach to capture dependencies between variables^85^. Here, it quantifies how much information one factor holds about ‘export market share’, indicating the strength of statistical dependence between them^86^. Factors with higher mutual information coefficients provide greater information on export; thus, they are identified as the informative characteristics of export share.

Eq. (1) is the mutual information regression formula where *X* denotes an individual factor, *Y* represents ‘export market share’, and *MI*(*X*; *Y*) is the mutual information coefficient. *p*(*x*, *y*) is the joint probability density function, and *p*(*x*) and *p*(*y*) are the marginal probability density functions of *X* and *Y*, respectively (please refer to the SI for the formulation of mutual information regression for discrete variables).

1 $$MI(X;Y)={\int }_{\mathcal Y}{\int }_{\mathcal X}p(x,y){\mathrm{log}}\left(\frac{p(x,y)}{p(x)p(y)}\right)dxdy$$

### Future projections

To predict the future positioning of agri-food exporters within the global trade system, we first compute 2035 projections of the informative characteristics of export share. Following, we calculate the 2035 projections of ‘export market share’ for 25 countries collectively (please refer to the SI for a detailed comparison explanation of our approach vs. estimating export shares individually across countries). For the projections of the informative characteristics, we employ the Neural Hierarchical Interpolation for Time Series model (NHITS)^87^. NHITS is a machine learning model that is designed for time series data^88^ as it is effective in capturing and combining short-term fluctuations and long-term trends, which leads to high accuracy and stable forecasts^89^ (please refer to text and Tables S2–S4 in SI for a more detailed explanation, formulation, parameter tuning, and accuracy of the NHITS model).

For ‘export market share’ projections, we utilize random forest regression, which is a non-parametric machine learning method to model complex relationships and predict future values^90^. It aggregates the predictions—‘export market share’ values in our case—from multiple decision trees^91^, as averaging across decision trees produces a more robust and accurate forecast while eliminating the risk of overfitting^92^ (please refer to text and Tables S5–S7 in SI for a more detailed explanation, formulation, parameter tuning, and accuracy of the random forest regression model).

### Regional supply concentrations

To assess the supply concentration of exporters, we implement the Shannon diversity index, which is widely used across domains to quantify evenness^93^. Here, this index measures how evenly an exporter distributes its supplies across various importers grouped in geographic regions. Thus, it captures both the number of exported regions and the evenness of supply amount distributions. As formulated in Eq. (2), *H* is the Shannon diversity index and *p*_*i*_ is the percentage of supplies directed to importing region *i*. *H* is computed for each exporter-year combination, and higher values indicate less concentrated - more diverse—distribution of exports.

2 $$H=-\mathop{\sum }\limits_{i=1}^{N}{p}_{i}\,{\rm{ln}}\,{p}_{i}$$

## Supplementary information

Supplementary Information

## Data Availability

All data sources are listed in the methods section of the paper and are freely available online. The export market share data is collected from [FAO] (https://www.fao.org/faostat/en). The GDP data is gathered from [World Bank] (https://data.worldbank.org/indicator/NY.GDP.MKTP.CD?most_recent_year_desc=true). Data for the government reliability index is drawn from [World Bank] (https://www.worldbank.org/en/publication/worldwide-governance-indicators). Data on the logistics performance index is retrieved from [World Bank] (https://lpi.worldbank.org/index.php/international). Data regarding the number of trade agreements is derived from [World Bank] (https://datatopics.worldbank.org/dta/table.html). Population data is obtained from [US Census] (https://www.census.gov/data-tools/demo/idb/?dashboard_page=country&COUNTRY_YR_ANIM=2025). Data on the food, beverage, and tobacco subsector’s value added within the total manufacturing sector is sourced from [World Bank] (https://databank.worldbank.org/metadataglossary/world-development-indicators/series/NV.MNF.FBTO.ZS.UN). Data on infrastructure investment and maintenance in transport is aggregated from [World Bank] (https://prosperitydata360.worldbank.org/en/indicator/GIH+GIO+24) and two different databases from [OECD]. Data on the total value of crop, animal, and aquaculture production is procured from [USDA] (https://www.ers.usda.gov/data-products/international-agricultural-productivity). Data for the regional export shares is computed based on [FAO] (https://www.fao.org/faostat/en). Data on disaster days is obtained from [International Disaster Database] (https://public.emdat.be). Data regarding the geopolitical risk index is sourced from [American Economics Association] (https://www.matteoiacoviello.com/gpr.htm) and data for the agriculture, forestry, and fishing, annual value added is gathered from [World Bank] (https://data.worldbank.org/indicator/NV.AGR.TOTL.KD.ZG).

## Code availability

In this study, informative predictor identification, future projections, and supply concentration analysis of export shares are performed using Python in Jupyter Notebook version 7.2.2. All code will be made publicly available at the first author’s GitHub page and the access link will be included here upon acceptance. All code is publicly available at https://github.com/Amirho3einghr/Underlying-export-characteristics-and-projected-country-positions-in-the-agri-food-trade.
