# Supplementary material for: Underlying export characteristics and projected country positions in the agri-food trade: A global system analysis
Source: NPJ Sci Food. 2026 Jun 26;10:225. doi: 10.1038/s41538-026-00944-y (PMC13385785; doi:10.1038/s41538-026-00944-y)
Supplement: Supplementary file 1 — Supplementary Information [file 41538_2026_944_MOESM1_ESM.pdf]

Supplementary Information

Underlying export characteristics and projected country positions in the agri-food trade: A global system analysis

Amirhosein Ghozatfar[1], Tina Sardashti[2], and Deniz Berfin Karakoc*[3]

[1]Department of Management Science,
University of Strathclyde, Glasgow, UK
[2]STOR-i Centre for Doctoral Training,
Lancaster University, Lancaster, UK
[3]School of Computing and Augmented Intelligence,
Arizona State University, Tempe, AZ USA

E-mail: dkarakoc@asu.edu

Input data

To assess government reliability, we calculate an average of the Worldwide Governance Indicators providing six dimensions of governance, including Voice and Accountability, Political Stability and Absence of Violence/Terrorism, Government Effectiveness, Regulatory Quality, Rule of Law, and Control of Corruption. The Worldwide Governance Indicators aggregate subjective perceptions from more than 30 original data sources, including surveys of households and firms, expert assessments by commercial providers, non-governmental organizations, and public sector institutions. These sources consistently measure key aspects of governance quality across countries, and are rescaled and merged using an Unobserved Components Model to form the six aggregate indicators. Moreover, these indicators measure governance on a scale where the global average is set to zero for each period. The scores are relative, reflecting how each country compares to others at that time, similar to Export Market Shares, which are also calculated in relation to the performance of other countries in each period. Voice and accountability reflect how freely citizens can choose their government and enjoy freedoms of expression, association, and a free media. Political stability and absence of violence/terrorism reflect perceptions of how likely a country is to experience political unrest or violence, including terrorism. Government effectiveness reflects perceptions of the quality of public services,

civil service independence, policy formulation and implementation, and the government's credibility in its commitments. Regulatory quality reflects perceptions of the government's ability to create and enforce policies and regulations that support private sector growth. The rule of law reflects perceptions of confidence in societal rules, including the quality of contract enforcement, property rights, the police, courts, and the likelihood of crime and violence. Control of corruption reflects perceptions of how much public power is used for private gain, including petty and grand corruption and state capture by elites or private interests. The constructed composite governance measures are scaled as standard normal variates, with a mean of zero, a standard deviation of one, and an approximate range of -2.5 to 2.5. In this framework, elevated values correspond to superior governance performance [1].

The Logistics Performance Index (LPI) scores countries on six key trade dimensions, which are derived from a survey of logistics professionals, assessing customs performance (efficiency of customs and border management clearance, including the speed, simplicity, and predictability of formalities), the quality of trade and transport infrastructure (e.g., ports, railroads, roads, information technology), the ease of arranging competitively priced shipments, the competence and quality of logistics services (trucking, forwarding, and customs brokerage), the ability to track and trace consignments, and the timeliness of shipments reaching their destinations within the scheduled or expected delivery time. The six core indicators are rated on a scale of very low (1) to very high (5). The overall LPI score is calculated as the weighted mean of national scores across the six core dimensions [2].

To obtain costs of Infrastructure Investment and Maintenance in Transport, we utilize a combination of databases offered by the World Bank Group's Prosperity Data360 [3] and the Organization for Economic Co-operation and Development [4] and [5], performing necessary calculations to derive the required metrics and a comprehensive database. More specifically, we extract the 'Expenditure on transport, Percent of GDP' indicator from the World Bank Group's Prosperity Data360, focusing only on entries where the Attribute 1 column specifies 'Budgetary central government'. Since this indicator is expressed as a percentage of GDP, we multiply its values by the corresponding GDP figures [6] to convert them into absolute dollar amounts. Additionally, values of two datasets from the Organization for Economic Co-operation and Development are aggregated across different modes of transport per country-year pairs, and their values are converted from euros to dollars using the respective annual average exchange rates. The final consolidated database prioritizes data from the World Bank Group's Prosperity Data360; when data is missing,

it is supplemented with the figures from the Organisation for Economic Co-operation and Development.

To calculate the Number of Trade Agreements, we count the number of trade agreements each country has in each year [7].

We utilize data from the United States Department of Agriculture (USDA)'s Economic Research Service (ERS) International Agricultural Productivity data product (an attribute of 'Outall_Q' in the referenced database) for the Total Value of Crop, Animal, and Aquaculture Production. Most of the data on production comes from United Nations organizations, namely the Food and Agriculture Organization (FAO) and the International Labor Organization (ILO). These are supplemented with data from national statistical agencies, business organizations, and published academic studies [8]. Its unit is constant 2015 global average farmgate prices, in $1000 Purchasing-Power-Parity (PPP) dollars, meaning that the values are adjusted for inflation to 2015 prices and converted using PPP exchange rates. Purchasing-Power-Parity ensures that the dollar values reflect equivalent purchasing power across countries by accounting for differences in local price levels, allowing for meaningful comparisons of farmgate prices worldwide.

The Geopolitical Risk (GPR) Index, developed by Dario Caldara and Matteo Iacoviello, provides a quantitative measure of adverse geopolitical events and the associated risks as perceived through the lens of the English-speaking press. The index is constructed by systematically tallying the frequency of newspaper articles covering geopolitical tensions and related topics, then expressing this as a share of the total number of articles published. Specifically, the Recent GPR Index is compiled using automated text searches of the electronic archives of ten major newspapers: The Chicago Tribune, The Daily Telegraph, Financial Times, The Globe and Mail, The Guardian, The Los Angeles Times, The New York Times, USA Today, The Wall Street Journal, and The Washington Post. For the Historical GPR Index, three newspapers of The New York Times, The Chicago Tribune, and The Washington Post are used to extend the measure further back in time. The index captures a broad range of geopolitical risks by organizing news coverage into eight categories: (1) War Threats, (2) Threats to Peace, (3) Military Buildups, (4) Nuclear Threats, (5) Terror Threats, (6) Outbreak of War, (7) Escalation of War, (8) Terrorist Acts. Formally, the index is calculated as in Eq. S1 where G is the number of articles mentioning adverse geopolitical events, and U is the total number of articles published in a given period. By design, higher GPR values indicate greater current intensity of adverse geopolitical events, a higher perceived likelihood of future conflicts, and greater expected severity of such events. In our study, we employ the recent version

of the GPR Index and compute annual averages by aggregating the monthly data for each country [9] and [10].

$$\text{GPR} \propto \frac{U}{G} \tag{S1}$$

The data of disasters are sourced from diverse organizations, including United Nations (UN) agencies, non-governmental organizations (NGOs), insurance companies, research institutes, and press agencies, and are managed and distributed by the Centre for Research on the Epidemiology of Disasters (CRED) with support from the United States Agency for International Development (USAID) [11]. To calculate Disaster Days, we use the database's natural classification and compute the percentage of days each year that each country experiences any type of disaster.

Our trade data are sourced from the FAOSTAT Detailed Trade Matrix (TM) database, which includes all FAO-defined agricultural and food commodities reported in the TM dataset, in nominal values. Thus, our commodity coverage corresponds to the full set of agri-food items covered by FAOSTAT, which are listed in Table S1 with their standardized classification codes. We include every single food item under each one of these food groups in our study to calculate agri-food export shares. Export Market Share is calculated by dividing each country's export value by the total export value for all countries in that year [12]. Export market shares in the baseline analysis are computed using export values reported in current USD, consistent with other explanatory predictors, which are mostly in USD, and standard practice in macro-level trade analyses that focus on realized trade influence rather than physical volumes; moreover, these values are nominal. As we adopt a macro, policy-oriented perspective and focus on export market shares measured in value terms, consistent with the reported values by FAOSTAT TM, these value-based measures are used intentionally to capture countries' economic presence and influence in global agri-food trade, rather than physical supply volumes. We acknowledge that nominal export values may reflect price dynamics and commodity composition in addition to quantities traded.

To address missing values in the datasets, we apply linear interpolation. It is a method of estimating an unknown value that lies between two known values on a straight line. It assumes that the change between the two points is linear and uses the formula of a straight line to compute the intermediate value. In this context, y_0 and y_1 are the known values of the variable, while x_0 and x_1 are the known periods. Mathematically, for two known

points (x_0, y_0) and (x_1, y_1), the interpolated value y at a point x is [13]:

$$y = y_0 + (x - x_0) \cdot \frac{(y_1 - y_0)}{(x_1 - x_0)} \tag{S2}$$

We apply linear interpolation to estimate missing values for each country–variable pair and for each country-partner pair in supply concentration analysis.

We standardize all key informative predictors before predicting them to have a mean of zero and a standard deviation of one by Z-score Normalization, allowing for comparability across variables with different units and scales. For a variable x, the Z-score normalization is defined as:

$$z = \frac{x - \mu}{\sigma} \tag{S3}$$

where x is the original value, μ is the mean of the variable, σ is the standard deviation, and z is the standardized value [14].

For the section in which we predict the future values of the key informative predictors, we construct a unique identifier for each country–variable pair. This ensures that the Neural Hierarchical Interpolation for Time Series (NHITS) forecasting model can learn each time series independently while leveraging shared dynamics across all series. Each unique identifier is grouped along with its corresponding values and year, forming the final columns that serve as input to the model. This structure allows NHITS to capture country-specific trends and variable-specific temporal patterns more effectively, improving the accuracy of multi-series forecasts [15].

In the data preprocessing stage for predicting Export Market Share, we remove the year column because the predicted key informative predictors already incorporate the time dimension and are intended to forecast the export share for each corresponding year. Moreover, we apply one-hot encoding to the country variable, transforming it into a set of binary indicators to capture country-specific fixed effects within the model. One-hot encoding is employed to transform the nominal country variable into a set of binary dummy variables. For each unique country, a separate binary column is generated, indicating the presence or absence of that country in each observation. This transformation ensures that the categorical information is represented numerically without introducing any artificial ordinal structure, thereby allowing the learning algorithms to capture country-specific fixed effects and heterogeneity [16]. We also apply this to the predicted key informative predictors that will be used to forecast the corresponding Export Market Share, to maintain alignment with the trained model used for Export Market Share prediction.

To analyze supply concentration for the selected countries based on the database of the Food and Agriculture Organization of the United Nations (FAO) [17], we first aggregate the export values for all agri-food commodities by country-partner-year. Next, we compute the total export value for each country and for each region within each year. We then calculate the share of each region by dividing its export value by the respective country's total export value for that year. Finally, we compute the average regional shares for each country over the period 1988–2022.

Lastly, some re-exporting or trans-shipment economies - particularly within Europe - are included in the analysis. There are two main reasons associated with this approach: one is scope-relevant, and the other is data limitation-relevant. Firstly, global trade statistics generally do not provide consistent, disaggregated export versus re-export data for all agri-food commodities and countries, which makes it infeasible to separate these flows in our study. Data challenges in tracking re-exports and processing trade at the global level are widely recognized in the trade literature. The purpose of our operationalization is not to conflate these export structures, nor to interpret export market share as a direct proxy for domestic production competitiveness. Rather, consistent with the managerial and policy-oriented perspective of the study, export value–based market shares are used as an outcome measure of realized participation and influence in agri-food trade, regardless of whether this influence arises from production, processing, or trade intermediation roles. From a policy and strategic standpoint, this distinction is important but not contradictory to our framework. Countries often pursue different pathways to expanding their presence in agri-food trade, through production upgrading, value-added processing, or investments in logistics, infrastructure, and trade facilitation. Our analysis is designed to capture these pathways jointly, reflecting how national export performance is commonly assessed at the macro policy level. This is consistent with macro trade-system perspectives where overall export performance is treated as an indicator of trade influence. Re-export hubs are acknowledged in the literature as significant nodes in global trade, serving as regional trade and logistics centers that facilitate trade flow [1,2]. Focusing on aggregated agri-food trade helps reduce the impact of outliers driven by specific commodity production or specialized logistics roles, allowing us to emphasize general trade-system patterns rather than getting misled by sector-specific anomalies.

However, constraints related to data availability and completeness do not limit the generality of the proposed framework. Any exporter or importer with sufficiently long and consistent trade time series can be directly incorporated without modification of the methodology. As reporting coverage improves for currently excluded countries, including

emerging exporters or regions affected by geopolitical disruptions, they can be seamlessly integrated into future analyses. This would allow future applications to explicitly focus on primary exporters or production-based trade structures where suitable disaggregated data become available.

Key informative predictors

For discrete variables, the mutual information regression formulation is demonstrated in Eq. S4, where the integration in Eq. 1 that is dedicated for continuous variables is replaced by summation:

$$I(X;Y) = \sum_{y \in \mathcal{Y}} \sum_{x \in \mathcal{X}} p(x,y) \log \left(\frac{p(x,y)}{p(x)p(y)} \right) \tag{S4}$$

where $p(x, y)$ is the joint probability distribution function of X and Y, $p(x)$ is the marginal probability distribution function of X, and $p(y)$ is the marginal probability distribution function of Y.

Future predictions

The primary reason for our two-step approach is to establish a more generalizable and broadly applicable framework for forecasting export shares. This involves first forecasting key informative predictors and then using them to predict export shares. By training a single random forest regression model with a fixed set of informative predictors whose coefficients remain consistent across nations and years, we enable the projection of export shares for any country and future year simply by inputting the corresponding predictor values [18]. If we instead modeled export shares directly for each nation individually, the resulting regression functions would vary substantially from one country to another and across years. This would undermine the identification of a consistent set of key informative predictors because the importance of predictors could fluctuate or even disappear in some individual models. In other words, the explanatory power and interpretability of the predictors would be fragmented and less reliable. Our approach, by contrast, leverages the predicted key informative predictors as inputs to a unified model, allowing us to capture broad global trends while accommodating national-level variations through the predictors themselves [19]. This approach enables the model to be readily generalized and applied across diverse countries, commodity categories, and temporal horizons without the necessity of retraining separate models for each specific instance. Conversely, predicting

each nation's export share independently with distinct regression models would limit the analysis to isolated case studies, thereby precluding the development of a unified machine learning framework capable of broad applicability across varying nations, commodities, time periods, and analytical contexts.

For the projections of the key informative predictors, we specifically employ the Neural Hierarchical Interpolation for Time Series model (NHITS). NHITS builds upon the successful Neural Basis Expansion Analysis for Interpretable Time Series Forecasting (N-BEATS) architecture by introducing novel hierarchical interpolation and multi-rate data sampling techniques. This model allows us to effectively predict the future values of the key informative predictors, which then serve as essential inputs for our subsequent analysis of predicting 2035 Export Market Shares.

At its core, NHITS operates by decomposing the forecasting problem into a sum of contributions from multiple stacked blocks. Each block learns to perform both a forecast ($\hat{\mathbf{f}}_b$) and a backcast ($\hat{\mathbf{b}}_b$), where the backcast portion is used to remove learned components from the input time series for the subsequent block, allowing each block to focus on the residual or unexplained patterns. The hierarchical interpolation mechanism processes input series at different frequencies, allowing it to capture both short-term fluctuations and long-term trends effectively.

Let $\mathbf{x}_{t-L:t}$ be the input time series window of length L (history) available up to time t, and $\hat{\mathbf{y}}_{t+1:t+H}$ be the forecasted output sequence of length H. The NHITS architecture comprises B stacked blocks.

The overall prediction of the NHITS model for the future horizon H can be conceptually represented as an additive decomposition:

$$\hat{\mathbf{y}}_{\text{NHITS}}(\mathbf{x}) = \sum_{b=1}^{B} \hat{\mathbf{f}}_b(\mathbf{x}_{b-1}) \tag{S5}$$

where $\hat{\mathbf{y}}_{\text{NHITS}}(\mathbf{x})$ is the final forecast sequence of length H generated by the NHITS model, B is the total number of stacked blocks in the architecture, and $\hat{\mathbf{f}}_b(\cdot)$ represents the forecast component generated by the b-th block. $\mathbf{x}_{b-1}$ is the input to the b-th block; for the first block ($b = 1$), $\mathbf{x}_0 = \mathbf{x}_{t-L:t}$ (the original input history), and for subsequent blocks ($b > 1$), $\mathbf{x}_{b-1}$ is the residual backcast from the previous block, meaning it represents the portion of the input time series that was not explained by the preceding blocks, specifically, after each block b, the original input $\mathbf{x}_{t-L:t}$ is updated by subtracting its backcast component $\hat{\mathbf{b}}_b$.

Each block b internally consists of Deep Neural Networks (fully-connected layers that learn to transform the input), a Forecast Head ($\hat{\mathbf{f}}_b$) that generates the forecast for the future horizon, a Backcast Head ($\hat{\mathbf{b}}_b$) that generates a "backcast" for the input window (this backcast is subtracted from the input, passing the residual to the next block, enabling the model to learn different components of the series sequentially), and Hierarchical Interpolation. Crucially, NHITS incorporates an interpolation layer designed to operate at multiple scales through methods like upsampling (increasing the data's resolution to capture fine details) and downsampling (reducing the data's resolution to discern broader trends), which enables the model to effectively capture hierarchical patterns across various frequencies within the time series, allowing it to understand both rapid fluctuations and slow-moving cycles simultaneously.

In brief, NHITS works by iteratively extracting and forecasting components from the time series, passing the unexplained residuals to subsequent blocks, and utilizing a multi-rate interpolation strategy to enhance its ability to model diverse temporal patterns.

We employ the NHITS model, sourced from the NeuralForecast library, to forecast time series data, which internally utilizes the PyTorch deep learning framework for its neural network computations. A comprehensive hyperparameter optimization process is conducted using the Optuna framework. This optimization targets three objectives: minimizing Root Mean Squared Error (RMSE), minimizing Mean Absolute Error (MAE), and maximizing the coefficient of determination (R^2, achieved by minimizing its negative value). The dataset is divided into distinct training and validation periods. The forecast horizon (h) for all predictions was consistently set to 13 time steps. We use a strict time-ordered hold-out split for the NHITS forecasting of informative predictors. Annual data from 1988–2017 are used for training, and the final five years, 2018–2022, are reserved for validation. Table S2 details the specific hyperparameter space explored during the Optuna optimization, which involves 150 trials. The best configuration, determined by minimizing the validation RMSE among the final results, is presented in Table S3. This configuration has achieved the validation metrics detailed in Table S4.

The final model is subsequently retrained on the full dataset using these best-found hyperparameters and then deployed for prediction. For reproducibility, a fixed random seed of 42 is employed during the final training of the NHITS model. Training has been efficiently conducted using a single GPU, specifically leveraging the Apple M3 Pro's Metal Performance Shaders backend. It is important to note that no external target scaling (`scaler_type=None`) or transformations are applied by the `NeuralForecast` library itself. The model utilizes identity stack layers (`stack_types=['identity']`)

and enables input padding (`start_padding_enabled=True`) for sequence alignment. Specifically, `start_padding_enabled=True` ensures that time series sequences shorter than the required input length are left-padded with zeros (or a neutral value), maintaining consistent input dimensionality across batches and preserving temporal alignment in early time steps.

For Export Market Share predictions, we utilize random forest regression, which is a non-parametric ensemble learning method, specifically a bagging algorithm, that models complex relationships and predicts future variables based on machine learning. It aggregates the predictions from multiple decision trees to produce a more robust and accurate forecast. More specifically, Random Forest Regression builds multiple decision trees to make predictions. Each tree is trained on a randomly drawn subset of the original dataset, using a method called bootstrap sampling, where some data points may appear multiple times and others not at all. At each split in the tree, only a random subset of the input features is considered. This process introduces variation among trees, making each one slightly different. In a single decision tree, the algorithm repeatedly splits the data into smaller groups based on thresholds in the input features, such as "greater than or less than" rules. The goal of each split is to group together samples that have similar target values. This process continues until the data in each group is as uniform as possible. These final groups are called "leaves". For each leaf, the model calculates the average of the target values (e.g., Export Market Share) in that group. This average becomes the predicted value for any new input that ends up in the same group. In a Random Forest, once all trees are trained, a new input is passed through each tree independently. Each tree produces its own prediction based on the leaf it falls into, and the final prediction is the average of all the individual tree outputs. In other words, for regression tasks, the final prediction of the Random Forest model for a given input $\mathbf{x}$ is the average of the predictions from all individual decision trees within the forest. This averaging process reduces the risk of overfitting and increases robustness, especially compared to using a single decision tree.

The core idea behind Random Forest is that while individual trees may exhibit high variance or be sensitive to noise, combining many diverse trees yields more stable and accurate predictions. Bootstrapping and feature randomness ensure that the forest captures a broader view of the data, making the overall model more generalizable and reliable. In essence, the Random Forest Regression model combines the strengths of multiple, slightly different, and uncorrelated decision trees, reducing variance and improving generalization compared to a single decision tree.

Each individual regression tree T_k (where $k = 1, \ldots, N_{\text{tree}}$) is trained on a

bootstrapped subset of the original training data and, at each split, considers only a random subset of features. For a given input vector $\mathbf{x}$, each tree T_k provides a prediction, denoted as $\hat{y}_k(\mathbf{x})$. The final prediction of the Random Forest Regression model, $\hat{Y}_{\text{RF}}(\mathbf{x})$, is then calculated as the average of the predictions from all individual trees:

$$\hat{Y}_{\text{RF}}(\mathbf{x}) = \frac{1}{N_{\text{tree}}} \sum_{k=1}^{N_{\text{tree}}} \hat{y}_k(\mathbf{x}) \tag{S6}$$

where $\hat{Y}_{\text{RF}}(\mathbf{x})$ is the predicted output of the Random Forest model for the input features $\mathbf{x}$, N_{tree} is the total number of decision trees in the ensemble (forest), and $\hat{y}_k(\mathbf{x})$ is the prediction made by the k-th individual decision tree for the input features $\mathbf{x}$. Each $\hat{y}_k(\mathbf{x})$ is typically the mean of the target values of the training samples that fall into the terminal leaf node for input $\mathbf{x}$ in tree T_k.

The final Random Forest Regressor is tuned on the full dataset using Bayesian Optimization with 5 initial random points and 15 optimization iterations. Namely, the hyperparameters for the Random Forest Regressor are optimized using Bayesian Optimization. The optimization aims to maximize the out-of-sample R^2 score on a test set (80/20 train-test split). Table S5 outlines the specific search ranges defined for each hyperparameter during this optimization process. Moreover, Table S6 presents the best-found hyperparameters that govern the architecture and training of the Random Forest model. The final Random Forest model, configured with the optimized hyperparameters, is trained on the full training set. The model is trained using `scikit-learn`'s `RandomForestRegressor` on a CPU-based environment to predict Export Market Share. For reproducibility, a fixed random seed of 42 is employed. It is important to note that no imputation, feature scaling, or target variable transformation is applied to the data. Cross-validation is performed on the training set using 10 folds, yielding consistently high R^2 scores with a mean of approximately 0.984, ranging between 0.9697 and 0.9933. The final model's performance on both the training and test sets is summarized in Table S7.

These robust results, particularly the high R^2 on both training and test the training and test sets and the low MSEs, confirm the model's strong fit to the training data and its excellent generalization These results demonstrate that the Random Forest model effectively captures underlying patterns in the data while maintaining robustness against overfitting.

Trends and key explanatory variables of agri-food export shares

In Table 2, the mutual information coefficients typically range from 0 to 2, where theoretically they can be higher, ranging from 0 upwards. As mutual information is measured in bits to quantify the shared information between variables, a coefficient value of 1 suggests that knowing the key explanatory variable reduces the uncertainty about the agri-food Export Market Share by 1 bit of information.

In our study, the mutual information coefficients range from 0 to 1.36. To facilitate interpretation, we categorize them into three categories due to the limited number of results: values between 1.36 and 0.91 are considered the most informative, those between 0.91 and 0.46 are considered moderately informative, and values from 0 to 0.46 are considered to have partial information.

To assess potential redundancy among explanatory variables, we evaluate linear multicollinearity using Variance Inflation Factors (VIF) computed on the pooled panel dataset. Multicollinearity diagnostics are relevant here as several macro-level indicators are structurally related in cross-country data. The resulting VIF values indicate moderate correlation among some predictors but do not suggest extreme or pathological multicollinearity (Table S9). A common heuristic in regression diagnostics is that VIF values below established thresholds, often taken as approximately 10, do not indicate severe multicollinearity among predictors, even when modest linear associations exist among explanatory variables [20], [21], [22], and [23]. Importantly, the purpose of this diagnostic is to verify that the explanatory space does not contain near-duplicate linear combinations that would undermine the stability of association-based analyses. Given that the second-stage model employs a non-parametric Random Forest, which is robust to correlated inputs, the observed levels of multicollinearity are consistent with the modeling strategy adopted in this study.

To further assess whether the explanatory variables highlighted in the analysis reflect meaningful structural dimensions, we conduct a Principal Component Analysis (PCA) on the pooled panel of explanatory variables. PCA is used here as an outcome-independent diagnostic tool to characterize the internal correlation structure of the predictor space, rather than to explain or predict export shares. All numeric explanatory variables are standardized prior to PCA, using z-scores, and the Export Market Share variable is excluded by construction. The results indicate that the explanatory space is structured but not dominated by a single latent factor (see Table S10 and S11). The first two principal components together account for approximately 60% of the total variance, with the leading

component explaining around one third of the variance. This suggests that correlated macro-level variables form coherent but genuinely multidimensional structures rather than collapsing into a single redundant dimension. Importantly, variables emphasised in the main analysis load strongly on the leading principal component, indicating that their associations with Export Market Shares are embedded within a meaningful macro-structural configuration of the explanatory space rather than arising from pathological multicollinearity. For interpretability, we report normalized weights derived from the first principal component by taking the absolute value of each variable's loading and rescaling these values to sum to one (i.e., the weight for variable j is computed as $|\ell_j|/\sum_k |\ell_k|$). These normalized weights quantify each variable's relative contribution to the dominant variance structure of the explanatory space and do not imply directionality, sign, or effect on export shares.

These results indicate that variables associated with Export Market Shares are structurally central within the explanatory space, while the absence of a single dominant principal component confirms that the predictor set is not driven by a one-dimensional macro variable. These variables should be understood as jointly characterizing the broader structural environment associated with Export Market Share outcomes.

The Mann-Kendall test is used to detect significant monotonic trends in the time-series data of the Export Market Shares and key informative predictors. The Mann-Kendall test is a non-parametric statistical test to detect trends in time series data without assuming any particular distribution [24]. The test outputs several key parameters. *Trend* indicates the direction of change over time; *increasing* suggests a statistically detectable upward trend, *decreasing* indicates a downward trend, and *no trend* means no consistent pattern was found. *P-value (P)* measures the probability that the observed trend occurred by chance; a smaller value (commonly below 0.05) indicates statistical significance. *Slope* represents the estimated magnitude of the trend per unit time (per year in this study), indicating how fast the variable is increasing or decreasing. *Kendall's Tau (τ)* is a rank correlation coefficient ranging from -1 to 1 that quantifies the strength and direction of the trend: values close to 1 indicate a strong increasing trend, close to -1 a strong decreasing trend, and near 0 little or no trend. The *Significant* column (True/False) is a binary interpretation of the P-value, highlighting whether the trend is statistically reliable. These metrics offer a robust view of how a variable behaves over time. Table S13-S37 presents the Mann-Kendall test results for export shares and key informative predictors for each one of the 25 nations individually.

Table S1. Central Product Classification (CPC) codes for the agri-food commodity groups we considered in their entirety in this study, obtained from UNCOMTRADE. For a detailed breakdown of individual food items under each group that is considered in this study, please refer to UNCOMTRADE.

Commodity Name	CPC Group Code
Cereals	011
Vegetables	012
Fruits and nuts	013
Oilseeds and oleaginous fruits	014
Edible roots and tubers with high starch or inulin content	015
Stimulant, spice and aromatic crops	016
Pulses (dried leguminous vegetables)	017
Sugar crops	018
Live animals	021
Raw milk	022
Eggs of hens or other birds in shell, fresh	023
Fish live, fresh or chilled for human consumption	042
Crustaceans, live, fresh or chilled	043
Molluscs live, fresh or chilled	044
Other aquatic invertebrates, live, fresh or chilled	045
Meat and meat products	211
Prepared and preserved fish, crustaceans, molluscs and other aquatic invertebrates	212
Prepared and preserved vegetables, pulses and potatoes	213
Prepared and preserved fruits and nuts	214
Animal fats	215
Vegetable oils	216
Margarine and similar preparations	217
Processed liquid milk, cream and whey	221
Other dairy products	222
Eggs, in shell, preserved or cooked	223
Grain mill products	231
Starches and starch products; sugars and sugar syrups	232
Bakery products	234
Sugar and molasses	235
Cocoa, chocolate and sugar confectionery	236
Macaroni, noodles, couscous and similar farinaceous products	237
Food products	239
Wines	242
Malt liquors and malt	243
Soft drinks; bottled mineral waters	244

Table S2. NHITS hyperparameter search space. The `input_size` specifies the number of historical time steps provided as input for forecasting. `max_steps` determines the total number of training iterations. The `learning_rate` controls the speed at which the model updates its weights during training. The `batch_size` influences both training stability and computational efficiency. Larger batch sizes can improve gradient estimation but may require more memory. The `dropout_prob_theta` applies dropout regularization to the parameterized outputs to prevent overfitting. This helps improve generalization by randomly deactivating neurons during training. The parameters `n_pool_kernel_size` and `n_freq_downsample` define the pooling kernel sizes and downsampling factors across the model's multi-resolution blocks, respectively, shaping how temporal information is compressed and processed. These are expressed as 3-element tuples (e.g., (2, 2, 1)) that correspond to the settings for each of the model's three hierarchical levels, allowing distinct configurations per resolution scale. The `n_blocks` setting controls the depth of the model at each hierarchical level, enabling flexible capacity adjustment; deeper configurations allow the model to capture more complex temporal patterns. Like the previous parameters, `n_blocks` is also specified as a tuple (e.g., (3, 3, 3)) indicating the number of blocks used at each of the three levels, facilitating structured multi-scale learning. The `mlp_units` specifies the number of hidden units per layer in the model's Multi-Layer Perceptron (MLP), which is a feedforward neural network used for non-linear transformation of temporal features. The MLP serves as a decoder for aggregating learned temporal features and producing interpretable output components.

Hyperparameter	Search Space / Value
`input_size`	$\{5, 10, 14, 17\}$
`max_steps`	$[500, 3000]$ (integer range)
`learning_rate`	$[1 \times 10^{-4}, 1 \times 10^{-2}]$ (log-uniform)
`batch_size`	$\{32, 64\}$
`dropout_prob_theta`	$[0.0, 0.2]$ (continuous range)
`n_pool_kernel_size`	$\{(2, 2, 1), (2, 1, 1)\}$
`n_freq_downsample`	$\{(1, 1, 1), (2, 1, 1)\}$
`n_blocks`	$\{(1, 1, 1), (2, 2, 2), (3, 3, 3), (4, 4, 4)\}$
`mlp_units`	$\{(128, 128)\}$ (repeated per block layer)

Table S3. Optimal NHITS hyperparameter configuration.

Hyperparameter	Optimal Value
`input_size`	10
`max_steps`	1924
`learning_rate`	0.00126
`batch_size`	64
`dropout_prob_theta`	0.197
`n_pool_kernel_size`	$(2, 1, 1)$
`n_freq_downsample`	$(1, 1, 1)$
`n_blocks`	$(2, 2, 2)$
`mlp_units`	$(128, 128)$ (repeated across all blocks)

Table S4. Validation metrics for the optimal NHITS model. The table reports the performance of the trained NHITS model on the validation set using three standard regression metrics. The Root Mean Squared Error (RMSE) and the Mean Absolute Error (MAE) both indicate high predictive accuracy, where lower values reflect smaller average prediction errors, with RMSE penalizing larger errors more heavily. The coefficient of determination (R^2) means that approximately 94.22% of the variance in the target variable is explained by the model. A higher R^2 value (closer to 1) denotes a better fit. Collectively, these metrics confirm that the NHITS model generalizes well and demonstrates strong forecasting performance.

**Metric**	**Value**
RMSE	0.3092
MAE	0.1096
R^2	0.9422

Table S5. Random forest regressor hyperparameter search space. The `max_depth` parameter limits the maximum depth of each decision tree, controlling model complexity and risk of overfitting. The `max_features` parameter determines the fraction of input features considered when looking for the best split at each node, affecting both randomness and accuracy. `min_samples_leaf` sets the minimum number of samples required at a leaf node, influencing tree pruning and generalization. The `min_samples_split` parameter specifies the minimum number of samples needed to split an internal node, affecting tree branching. Finally, `n_estimators` defines the number of trees in the ensemble, balancing predictive performance with computational cost.

**Hyperparameter**	**Type/ Search Range**
`max_depth` (Maximum Tree Depth)	Integer range: $[4, 20]$
`max_features` (Maximum Feature Fraction)	Continuous range: $[0.5, 1.0]$
`min_samples_leaf` (Minimum Samples per Leaf)	Integer range: $[1, 5]$
`min_samples_split` (Minimum Samples per Split)	Integer range: $[2, 10]$
`n_estimators` (Number of Trees)	Integer range: $[100, 1000]$

Table S6. Optimal random forest hyperparameters.

**Hyperparameter**	**Optimal Value**
`n_estimators` (Number of Trees)	528
`max_depth` (Maximum Tree Depth)	18
`max_features` (Maximum Feature Fraction)	≈ 0.50
`min_samples_leaf` (Minimum Samples per Leaf)	1
`min_samples_split` (Minimum Samples per Split)	7

Table S7. Random forest model performance metrics. This table reports the performance of the Random Forest model on both the training and test sets using two standard evaluation metrics. The coefficient of determination (R^2) measures the proportion of variance in the target variable that is explained by the model; values closer to 1 indicate a better fit. The Mean Squared Error (MSE) quantifies the average squared difference between predicted and actual values, where smaller values indicate higher accuracy.

Metric	Training Set	Test Set
R^2	0.996	0.983
MSE	3.06×10^{-6}	1.03×10^{-5}

Table S8. Historical global and study-specific export ranks by country. Each Export Market Share value represents a country's historical average, based on data observed during the study period.

Country	Export Share (%)	Historical Global Rank	Historical Rank Within This Study
United States	11.88%	1	1
Netherlands	7.52%	2	2
France	7.15%	3	3
Germany	5.99%	4	4
Brazil	4.54%	5	5
China	4.14%	6	6
Belgium	3.55%	7	7
Italy	3.48%	8	8
Spain	3.31%	9	9
Canada	3.27%	10	10
Australia	2.97%	11	11
United Kingdom	2.90%	12	12
Argentina	2.54%	13	13
Thailand	2.09%	14	14
Malaysia	1.75%	17	15
Mexico	1.62%	18	16
India	1.56%	19	17
Poland	1.24%	22	18
Ukraine	0.84%	25	19
Russia	0.73%	27	20
Chile	0.67%	29	21
Portugal	0.40%	38	22
Philippines	0.36%	41	23
Finland	0.18%	64	24
Tunisia	0.11%	76	25

Table S9. VIF diagnostics for explanatory variables. All VIF values remain below commonly cited multicollinearity thresholds, indicating no severe collinearity concerns among model covariates.

Explanatory Variable	VIF
Total Value of Crop, Animal, and Aquaculture Production	8.97
Logistics Performance Index	6.52
Population	6.48
Government Reliability Index	6.18
GDP	2.44
Share of Food, Beverage, and Tobacco Subsector's Value Added within the Total Manufacturing Sector	1.50
Number of Trade Agreements	1.24
Infrastructure Investment and Maintenance in Transport	1.03

Table S10. Explained variance ratios of the leading principal components. Values indicate the proportion of total variance captured by each component.

Principal Component	Explained Variance Ratio
PC1	0.313
PC2	0.292
PC3	0.131
PC4	0.099
PC5	0.092
PC6	0.054
PC7	0.010
PC8	0.008

Table S11. Normalized weights of explanatory variables on the first principal component. Normalized PC1 weights indicate relative contributions to the dominant variance structure.

Explanatory Variable	PC1 Weight
Government Reliability Index	0.205
Population	0.204
Total Value of Crop, Animal, and Aquaculture Production	0.199
Logistics Performance Index	0.157
Number of Trade Agreements	0.119
GDP	0.074
Share of Food, Beverage, and Tobacco Subsector's Value Added within the Total Manufacturing Sector	0.028
Infrastructure Investment and Maintenance in Transport	0.016

Table S12. Mann-Kendall test results for Export Market Share [Percentage] by country.

Country	Trend	P	Slope	Tau	Significant
Argentina	increasing	0.0332	0.018	0.253782	True
Australia	decreasing	6.30E-06	-0.039	-0.536134	True
Belgium	decreasing	1.46E-08	-0.041	-0.653782	True
Brazil	increasing	3.45E-10	0.1	0.744538	True
Canada	no trend	0.0608	9.14E-03	0.223529	False
Chile	increasing	2.92E-09	1.38E-02	0.704202	True
Germany	decreasing	8.97E-03	-2.25E-02	-0.310924	True
Finland	decreasing	5.51E-06	-2.79E-03	-0.539496	True
France	decreasing	7.10E-15	-1.99E-01	-0.922689	True
India	increasing	1.73E-09	4.95E-02	0.714286	True
Italy	no trend	0.1397	-9.07E-03	-0.176471	False
Malaysia	increasing	0.04085	7.63E-03	0.243697	True
Mexico	increasing	3.47E-09	4.14E-02	0.700840	True
Netherlands	decreasing	1.868E-08	-8.76E-02	-0.667227	True
China	no trend	0.9321	1.18E-03	0.011765	False
Philippines	decreasing	6.40E-03	-1.85E-03	-0.324370	True
Poland	increasing	5.77E-15	6.50E-02	0.926050	True
Portugal	increasing	9.95E-14	7.36E-03	0.882353	True
Russia	increasing	1.19E-06	3.74E-02	0.576471	True
Spain	increasing	1.58E-04	2.87E-02	0.448739	True
Thailand	increasing	3.19E-06	1.77E-02	0.552941	True
Tunisia	no trend	0.4777	3.49E-04	0.085714	False
Ukraine	increasing	8.6E-12	4.04E-02	0.808403	True
United Kingdom	decreasing	7.64E-12	-7.49E-02	-0.811765	True
United States	decreasing	8.13E-09	-1.47E-01	-0.684034	True

Table S13. Mann-Kendall test results for Argentina.

Independent Variable	Trend	P	Slope	Tau	Significant
LPI Score	no trend	0.1099	0.00	-0.171429	False
Number of Trade Agreements	increasing	6.49E-13	0.18	0.831933	True
Population	increasing	0.00	4.10E+05	1	True
Total Value of Crop, Animal, and Aquaculture Production [Thousand $ USD]	increasing	1.40E-14	1.32E+06	0.912605	True
Government Reliability Index	decreasing	1E-04	-0.01	-0.448739	True
GDP [$ USD]	increasing	1.86E-07	1.33E+10	0.618487	True
Share of Food, Beverage, and Tobacco Subsector's Value Added within the Total Manufacturing Sector [Percentage]	increasing	1.0E-04	0.22	0.452101	True
Infrastructure Maintenance and Investment in Transport [$ USD]	increasing	2.32E-07	1.94E+10	0.613445	True

Table S14. Mann-Kendall test results for Australia.

Independent Variable	Trend	P	Slope	Tau	Significant
LPI Score	decreasing	0.0319	0.00	-0.225210	True
Number of Trade Agreements	increasing	1.90E-12	0.40	0.798319	True
Population	increasing	0.00	2.92E+05	1	True
Total Value of Crop, Animal, and Aquaculture Production [Thousand $ USD]	increasing	4.89E-09	4.84E+05	0.694118	True
Government Reliability Index	decreasing	1.8E-03	-2.27E-03	-0.368067	True
GDP [$ USD]	increasing	5.64E-12	4.23E+10	0.816807	True
Share of Food, Beverage, and Tobacco Subsector's Value Added within the Total Manufacturing Sector [Percentage]	increasing	1.38E-06	0.23	0.573109	True
Infrastructure Maintenance and Investment in Transport [$ USD]	increasing	2.55E-10	2.20E+10	0.724370	True

Table S15. Mann-Kendall test results for Belgium.

Independent Variable	Trend	P	Slope	Tau	Significant
LPI Score	increasing	1.66E-08	4.9E-03	0.6	True
Number of Trade Agreements	increasing	2.22E-16	1.40	0.981513	True
Population	increasing	0.00	5.82E+04	1	True
Total Value of Crop, Animal, and Aquaculture Production [Thousand $ USD]	no trend	0.4477	0.00	0.089076	False
Government Reliability Index	decreasing	3.86E-07	-0.01	-0.596639	True
GDP [$ USD]	increasing	5.64E-12	1.29E+10	0.816807	True
Share of Food, Beverage, and Tobacco Subsector's Value Added within the Total Manufacturing Sector [Percentage]	no trend	0.3786	0.08	0.105882	False
Infrastructure Maintenance and Investment in Transport [$ USD]	increasing	3.87E-06	6.67E+07	0.544538	True

Table S16. Mann-Kendall test results for Brazil.

Independent Variable	Trend	P	Slope	Tau	Significant
LPI Score	increasing	2.93E-05	0.01	0.445378	True
Number of Trade Agreements	increasing	1.17E-13	0.23	0.865546	True
Population	increasing	0.00	2.17E+06	1	True
Total Value of Crop, Animal, and Aquaculture Production [Thousand $ USD]	increasing	2.22E-16	5.74E+06	0.973109	True
Government Reliability Index	decreasing	8.3E-03	-0.01	-0.310924	True
GDP [$ USD]	increasing	2.72E-07	5.39E+10	0.610084	True
Share of Food, Beverage, and Tobacco Subsector's Value Added within the Total Manufacturing Sector [Percentage]	increasing	5.56E-04	0.17	0.410084	True
Infrastructure Maintenance and Investment in Transport [$ USD]	decreasing	2.71E-08	-1.62E+10	-0.556303	True

Table S17. Mann-Kendall test results for Canada.

Independent Variable	Trend	P	Slope	Tau	Significant
LPI Score	decreasing	4.42E-07	-2.7E-03	-0.537815	True
Number of Trade Agreements	increasing	5.44E-14	0.42	0.877311	True
Population	increasing	0.00	3.26E+05	1	True
Total Value of Crop, Animal, and Aquaculture Production [Thousand $ USD]	increasing	2.34E-13	7.58E+05	0.868908	True
Government Reliability Index	decreasing	0.0304	-6.25E-04	-0.255462	True
GDP [$ USD]	increasing	9.06E-13	4.75E+10	0.847059	True
Share of Food, Beverage, and Tobacco Subsector's Value Added within the Total Manufacturing Sector [Percentage]	increasing	2.22E-04	0.15	0.438655	True
Infrastructure Maintenance and Investment in Transport [$ USD]	increasing	2.59E-07	4.72E+08	0.606723	True

Table S18. Mann-Kendall test results for Chile.

Independent Variable	Trend	P	Slope	Tau	Significant
LPI Score	no trend	0.06138	0.00	0.196639	False
Number Of Trade Agreements	increasing	1.55E-15	1.04	0.937815	True
Population	increasing	0.00	1.63E+05	1.00	True
Total Value of Crop, Animal, and Aquaculture Production [Thousand $ USD]	increasing	2.89E-15	4.02E+05	0.936134	True
Government Reliability Index	decreasing	3.78E-03	-5.42E-03	-0.341176	True
GDP [$ USD]	increasing	7.37E-13	8.94E+09	0.850420	True
Share of Food, Beverage, and Tobacco Subsector's Value Added within the Total Manufacturing Sector [Percentage]	increasing	3.40E-13	5.65E-01	0.862185	True
Infrastructure Maintenance and Investment in Transport [$ USD]	increasing	2.3E-09	1.13E+10	0.657143	True

Table S19. Mann-Kendall test results for Germany.

Independent Variable	Trend	P	Slope	Tau	Significant
LPI Score	increasing	2.41E-04	3.92E-04	0.391597	True
Number Of Trade Agreements	increasing	2.22E-16	1.40	0.981513	True
Population	increasing	1.34E-08	1.16E+05	0.673950	True
Total Value of Crop, Animal, and Aquaculture Production [Thousand $ USD]	increasing	0.03813	1.09E+05	0.247059	True
Government Reliability Index	decreasing	1.01E-06	-4.37E-03	-0.574790	True
GDP [$ USD]	increasing	7.07E-11	7.96E+10	0.773109	True
Share of Food, Beverage, and Tobacco Subsector's Value Added within the Total Manufacturing Sector [Percentage]	no trend	0.9885	0.00	-3.36E-03	False
Infrastructure Maintenance and Investment in Transport [$ USD]	increasing	1.66E-03	2.10E+08	0.371429	True

Table S20. Mann-Kendall test results for Finland.

Independent Variable	Trend	P	Slope	Tau	Significant
LPI Score	increasing	4.63E-05	1.94E-03	0.433613	True
Number Of Trade Agreements	increasing	8.88E-16	1.38	0.946218	True
Population	increasing	0.00	1.92E+04	1.00	True
Total Value of Crop, Animal, and Aquaculture Production [Thousand $ USD]	no trend	0.7547	-5.21E+02	-0.038655	False
Government Reliability Index	no trend	0.3818	2.50E-04	0.104202	False
GDP [$ USD]	increasing	9.27E-10	6.28E+09	0.726050	True
Share of Food, Beverage, and Tobacco Subsector's Value Added within the Total Manufacturing Sector [Percentage]	no trend	0.06085	-9.20E-02	-0.223529	False
Infrastructure Maintenance and Investment in Transport [$ USD]	no trend	0.7872	1.02E+07	0.033613	False

Table S21. Mann-Kendall test results for France.

Independent Variable	Trend	P	Slope	Tau	Significant
LPI Score	increasing	7.50E-07	2.96E-03	0.526050	True
Number Of Trade Agreements	increasing	2.22E-16	1.40	0.981513	True
Population	increasing	0.00	3.29E+05	1.00	True
Total Value of Crop, Animal, and Aquaculture Production [Thousand $ USD]	decreasing	0.01458	-1.03E+05	-0.290756	True
Government Reliability Index	decreasing	4.53E-03	-3.89E-03	-0.334454	True
GDP [$ USD]	increasing	7.76E-10	5.91E+10	0.729412	True
Share of Food, Beverage, and Tobacco Subsector's Value Added within the Total Manufacturing Sector [Percentage]	increasing	9.32E-12	2.01E-01	0.808403	True
Infrastructure Maintenance and Investment in Transport [$ USD]	no trend	0.6392	-2.63E+08	-0.057143	False

Table S22. Mann-Kendall test results for India.

Independent Variable	Trend	P	Slope	Tau	Significant
LPI Score	increasing	1.59E-07	1.39E-03	0.557983	True
Number Of Trade Agreements	increasing	4.51E-14	6.52E-01	0.878992	True
Population	increasing	0.00	1.70E+07	1.00	True
Total Value of Crop, Animal, and Aquaculture Production [Thousand $ USD]	increasing	2.22E-16	9.60E+06	0.969748	True
Government Reliability Index	no trend	0.4122	3.47E-04	0.097479	False
GDP [$ USD]	increasing	1.11E-15	9.16E+10	0.951261	True
Share of Food, Beverage, and Tobacco Subsector's Value Added within the Total Manufacturing Sector [Percentage]	decreasing	3.14E-03	-7.84E-02	-0.351261	True
Infrastructure Maintenance and Investment in Transport [$ USD]	no trend	0.1764	2.01E+09	0.161345	False

Table S23. Mann-Kendall test results for Italy.

Independent Variable	Trend	P	Slope	Tau	Significant
LPI Score	increasing	3.91E-09	5.29E-03	0.626891	True
Number Of Trade Agreements	increasing	2.22E-16	1.40	0.981513	True
Population	increasing	2.89E-13	1.72E+05	0.865546	True
Total Value of Crop, Animal, and Aquaculture Production [Thousand $ USD]	decreasing	2.98E-05	-2.03E+05	-0.495798	True
Government Reliability Index	decreasing	2.64E-12	-1.54E-02	-0.821849	True
GDP [$ USD]	increasing	5.34E-07	3.77E+10	0.594958	True
Share of Food, Beverage, and Tobacco Subsector's Value Added within the Total Manufacturing Sector [Percentage]	increasing	4.29E-07	9.84E-02	0.6	True
Infrastructure Maintenance and Investment in Transport [$ USD]	increasing	2.07E-03	4.48E+08	0.364706	True

Table S24. Mann-Kendall test results for Malaysia.

Independent Variable	Trend	P	Slope	Tau	Significant
LPI Score	decreasing	2.64E-03	0.00	-0.321008	True
Number Of Trade Agreements	increasing	8.66E-15	5E-01	0.910924	True
Population	increasing	0.00	5.25E+05	1.00	True
Total Value of Crop, Animal, and Aquaculture Production [Thousand $ USD]	increasing	2.18E-14	3.72E+05	0.905882	True
Government Reliability Index	no trend	0.05216	-1.73E-03	-0.228571	False
GDP [$ USD]	increasing	1.93E-14	1.09E+10	0.907563	True
Share of Food, Beverage, and Tobacco Subsector's Value Added within the Total Manufacturing Sector [Percentage]	no trend	0.05002	7.88E-02	0.233613	False
Infrastructure Maintenance and Investment in Transport [$ USD]	increasing	4.32E-09	9.82E+09	0.667227	True

Table S25. Mann-Kendall test results for Mexico.

Independent Variable	Trend	P	Slope	Tau	Significant
LPI Score	increasing	1.33E-06	6.67E-03	0.514286	True
Number Of Trade Agreements	increasing	1.55E-15	6E-01	0.939496	True
Population	increasing	0.00	1.47E+06	1.00	True
Total Value of Crop, Animal, and Aquaculture Production [Thousand $ USD]	increasing	2.22E-16	1.14E+06	0.979832	True
Government Reliability Index	no trend	0.07548	-4.82E-03	-0.210084	False
GDP [$ USD]	increasing	7.37E-13	3.62E+10	0.850420	True
Share of Food, Beverage, and Tobacco Subsector's Value Added within the Total Manufacturing Sector [Percentage]	decreasing	0.04836	-1.22E-01	-0.235294	True
Infrastructure Maintenance and Investment in Transport [$ USD]	increasing	0.02846	0.00	0.220168	True

Table S26. Mann-Kendall test results for Netherlands.

Independent Variable	Trend	P	Slope	Tau	Significant
LPI Score	decreasing	1.56E-06	-3.95E-03	-0.510924	True
Number Of Trade Agreements	increasing	2.22E-16	1.40	0.981513	True
Population	increasing	0.00	7.74E+04	1.00	True
Total Value of Crop, Animal, and Aquaculture Production [Thousand $ USD]	increasing	1.20E-03	7.02E+04	0.384874	True
Government Reliability Index	decreasing	2.07E-06	-6.32E-03	-0.557983	True
GDP [$ USD]	increasing	2.24E-11	2.29E+10	0.793277	True
Share of Food, Beverage, and Tobacco Subsector's Value Added within the Total Manufacturing Sector [Percentage]	decreasing	3.81E-05	-9.30E-02	-0.489076	True
Infrastructure Maintenance and Investment in Transport [$ USD]	no trend	0.7654	-1.08E+08	-0.036975	False

Table S27. Mann-Kendall test results for China.

Independent Variable	Trend	P	Slope	Tau	Significant
LPI Score	increasing	3.91E-09	1E-02	0.626891	True
Number Of Trade Agreements	increasing	1.16E-12	5.56E-01	0.806723	True
Population	increasing	0.00	7.33E+06	1.00	True
Total Value of Crop, Animal, and Aquaculture Production [Thousand $ USD]	increasing	0.00	2.58E+07	1.00	True
Government Reliability Index	no trend	0.2341	1.39E-03	0.141176	False
GDP [$ USD]	increasing	0.00	4.96E+11	0.998319	True
Share of Food, Beverage, and Tobacco Subsector's Value Added within the Total Manufacturing Sector [Percentage]	decreasing	1.22E-05	-6.78E-02	-0.519328	True
Infrastructure Maintenance and Investment in Transport [$ USD]	increasing	6.66E-16	2.65E+10	0.951261	True

Table S28. Mann-Kendall test results for Philippines.

Independent Variable	Trend	P	Slope	Tau	Significant
LPI Score	increasing	2.93E-05	7E-03	0.445378	True
Number Of Trade Agreements	increasing	5.37E-14	2.92E-01	0.878992	True
Population	increasing	0.00	1.65E+06	1.00	True
Total Value of Crop, Animal, and Aquaculture Production [Thousand $ USD]	increasing	2.18E-14	6.01E+05	0.905882	True
Government Reliability Index	decreasing	0.01481	-6.79E-03	-0.287395	True
GDP [$ USD]	increasing	7.99E-15	1.11E+10	0.921008	True
Share of Food, Beverage, and Tobacco Subsector's Value Added within the Total Manufacturing Sector [Percentage]	no trend	0.4432	-8.60E-02	-0.092437	False
Infrastructure Maintenance and Investment in Transport [$ USD]	increasing	6.33E-11	2.32E+10	0.774790	True

Table S29. Mann-Kendall test results for Poland.

Independent Variable	Trend	P	Slope	Tau	Significant
LPI Score	increasing	3.87E-09	1.68E-02	0.626891	True
Number Of Trade Agreements	increasing	5.74E-12	7.78E-01	0.766387	True
Population	no trend	0.9773	-1.65E+02	-5.04E-03	False
Total Value of Crop, Animal, and Aquaculture Production [Thousand $ USD]	no trend	0.4432	4.18E+04	0.092437	False
Government Reliability Index	no trend	0.07088	-4.96E-03	-0.213445	False
GDP [$ USD]	increasing	1.35E-14	1.87E+10	0.912605	True
Share of Food, Beverage, and Tobacco Subsector's Value Added within the Total Manufacturing Sector [Percentage]	decreasing	2.86E-03	-3.04E-01	-0.354622	True
Infrastructure Maintenance and Investment in Transport [$ USD]	no trend	0.9659	0.00	6.72E-03	False

Table S30. Mann-Kendall test results for Portugal.

Independent Variable	Trend	P	Slope	Tau	Significant
LPI Score	increasing	8.66E-05	2.50E-03	0.418487	True
Number Of Trade Agreements	increasing	2.22E-16	1.40	0.981513	True
Population	increasing	1.09E-03	1.70E+04	0.388235	True
Total Value of Crop, Animal, and Aquaculture Production [Thousand $ USD]	increasing	8.87E-05	2.66E+04	0.465546	True
Government Reliability Index	decreasing	6.98E-09	-1.28E-02	-0.680672	True
GDP [$ USD]	increasing	2.17E-10	5.73E+09	0.752941	True
Share of Food, Beverage, and Tobacco Subsector's Value Added within the Total Manufacturing Sector [Percentage]	increasing	0.02874	8.09E-02	0.260504	True
Infrastructure Maintenance and Investment in Transport [$ USD]	no trend	0.07166	-4.10E+07	-0.213445	False

Table S31. Mann-Kendall test results for Russia.

Independent Variable	Trend	P	Slope	Tau	Significant
LPI Score	increasing	4.73E-09	1.25E-02	0.623529	True
Number Of Trade Agreements	increasing	6.02E-04	1.38E-01	0.4	True
Population	decreasing	1.38E-06	-2.12E+05	-0.573109	True
Total Value of Crop, Animal, and Aquaculture Production [Thousand $ USD]	increasing	0.04085	8.83E+05	0.243697	True
Government Reliability Index	no trend	0.06341	-1.67E-03	-0.218487	False
GDP [$ USD]	increasing	1.91E-05	5.17E+10	0.507563	True
Share of Food, Beverage, and Tobacco Subsector's Value Added within the Total Manufacturing Sector [Percentage]	decreasing	0.02933	-4.93E-02	-0.258824	True
Infrastructure Maintenance and Investment in Transport [$ USD]	increasing	6.33E-09	2.05E+10	0.682353	True

Table S32. Mann-Kendall test results for Spain.

Independent Variable	Trend	P	Slope	Tau	Significant
LPI Score	increasing	4.48E-10	9.69E-03	0.663866	True
Number Of Trade Agreements	increasing	2.22E-16	1.40	0.981513	True
Population	increasing	1.11E-14	2.77E+05	0.915966	True
Total Value of Crop, Animal, and Aquaculture Production [Thousand $ USD]	increasing	1.73E-09	5.81E+05	0.714286	True
Government Reliability Index	decreasing	6.05E-07	-1.30E-02	-0.586555	True
GDP [$ USD]	increasing	1.71E-08	3.31E+10	0.668908	True
Share of Food, Beverage, and Tobacco Subsector's Value Added within the Total Manufacturing Sector [Percentage]	increasing	9.75E-03	8.57E-02	0.307563	True
Infrastructure Maintenance and Investment in Transport [$ USD]	no trend	0.1321	-9.51E+09	-0.179832	False

Table S33. Mann-Kendall test results for Thailand.

Independent Variable	Trend	P	Slope	Tau	Significant
LPI Score	no trend	0.2962	0.00	0.112605	False
Number Of Trade Agreements	increasing	1.66E-14	4.40E-01	0.902521	True
Population	increasing	0.00	4.67E+05	1.00	True
Total Value of Crop, Animal, and Aquaculture Production [Thousand $ USD]	increasing	1.89E-13	9.03E+05	0.872269	True
Government Reliability Index	decreasing	1.37E-04	-1.98E-02	-0.448739	True
GDP [$ USD]	increasing	5.98E-13	1.43E+10	0.853782	True
Share of Food, Beverage, and Tobacco Subsector's Value Added within the Total Manufacturing Sector [Percentage]	no trend	0.2004	1.44E-02	0.152941	False
Infrastructure Maintenance and Investment in Transport [$ USD]	increasing	8.14E-06	1.51E+10	0.529412	True

Table S34. Mann-Kendall test results for Tunisia.

Independent Variable	Trend	P	Slope	Tau	Significant
LPI Score	no trend	0.1837	0.00	-0.142857	False
Number Of Trade Agreements	increasing	5.37E-12	2.22E-01	0.788235	True
Population	increasing	0.00	1.11E+05	1.00	True
Total Value of Crop, Animal, and Aquaculture Production [Thousand $ USD]	increasing	4.41E-11	1.14E+05	0.781513	True
Government Reliability Index	decreasing	1.40E-05	-4.11E-03	-0.510924	True
GDP [$ USD]	increasing	4.83E-11	1.22E+09	0.779832	True
Share of Food, Beverage, and Tobacco Subsector's Value Added within the Total Manufacturing Sector [Percentage]	increasing	2.62E-04	1.49E-01	0.433613	True
Infrastructure Maintenance and Investment in Transport [$ USD]	increasing	1.75E-08	1.05E+09	0.657143	True

Table S35. Mann-Kendall test results for Ukraine.

Independent Variable	Trend	P	Slope	Tau	Significant
LPI Score	increasing	1.25E-07	7.04E-03	0.563025	True
Number Of Trade Agreements	increasing	8.66E-15	5.52E-01	0.910924	True
Population	decreasing	2.22E-15	-2.99E+05	-0.939496	True
Total Value of Crop, Animal, and Aquaculture Production [Thousand $ USD]	increasing	0.04679	3.81E+05	0.236975	True
Government Reliability Index	no trend	0.1688	-7.58E-04	-0.163025	False
GDP [$ USD]	increasing	8.35E-05	3.70E+09	0.467227	True
Share of Food, Beverage, and Tobacco Subsector's Value Added within the Total Manufacturing Sector [Percentage]	decreasing	5.26E-03	0.00	-0.263866	True
Infrastructure Maintenance and Investment in Transport [$ USD]	increasing	6.13E-08	5.44E+09	0.628571	True

Table S36. Mann-Kendall test results for the United Kingdom.

Independent Variable	Trend	P	Slope	Tau	Significant
LPI Score	no trend	0.83	0.00	-0.021849	False
Number Of Trade Agreements	increasing	6.66E-16	1.28	0.954622	True
Population	increasing	0.00	3.30E+05	1.00	True
Total Value of Crop, Animal, and Aquaculture Production [Thousand $ USD]	no trend	0.3634	1.81E+04	0.109244	False
Government Reliability Index	decreasing	5.14E-11	-1.08E-02	-0.771429	True
GDP [$ USD]	increasing	3.99E-11	6.96E+10	0.783193	True
Share of Food, Beverage, and Tobacco Subsector's Value Added within the Total Manufacturing Sector [Percentage]	no trend	0.07824	7.94E-02	0.210084	False
Infrastructure Maintenance and Investment in Transport [$ USD]	increasing	6.26E-06	1.02E+11	0.532773	True

Table S37. Mann-Kendall test results for United States.

Independent Variable	Trend	P	Slope	Tau	Significant
LPI Score	increasing	6.70E-07	1.72E-03	0.529412	True
Number Of Trade Agreements	increasing	4.02E-13	5E-01	0.842017	True
Population	increasing	0.00	2.72E+06	1.00	True
Total Value of Crop, Animal, and Aquaculture Production [Thousand $ USD]	increasing	6.46E-14	4.13E+06	0.889076	True
Government Reliability Index	decreasing	9.74E-09	-9.56E-03	-0.673950	True
GDP [$ USD]	increasing	0.00	5.25E+11	0.991597	True
Share of Food, Beverage, and Tobacco Subsector's Value Added within the Total Manufacturing Sector [Percentage]	increasing	1.11E-09	1.49E-01	0.722689	True
Infrastructure Maintenance and Investment in Transport [$ USD]	increasing	1.83E-11	3.42E+09	0.791597	True

Future projections of exporters' positioning in the agri-food trade

As shown in Figures S1- S9, Chile strengthens its export market advantage by 2035, evidenced by consistent growth in almost all of its explanatory variables. Statistics from other investigations like [25] and [26] confirm the development of the Chilean export capacities, especially in some specialized crops. The emergence of new strong competitors is the reason why Mexico is expected to lose its position in the agri-food export market slightly [25]. Stable or slow growth in key explanatory variables of Mexico can worsen this trend. Similarly to Mexico, as discussed, the stability of the United States in the agri-food export market is also explained by marginal progress in its trade Agreements, and almost steady production volumes, coupled with minor declines in the logistics performance and government reliability. However, it maintains a strong position due to the strength of its other key explanatory variables and their robust growth. For Argentina, reduced infrastructure investment and maintenance in transport, along with stable or slightly rising other key explanatory variables relative to other countries, cause a decline in its export market standing.

As to 'Infrastructure Investment and Maintenance in Transport', based on their historical trends, it is important to note that most countries experienced a significant decline during the COVID-19 pandemic; moreover, projections vary, showing stability, growth, decline, or volatility. More specifically, the Association of Southeast Asian Nations (ASEAN)'s planning indicates increased funding commitments for associated projects in the Philippines, Malaysia, and Thailand, supporting their corresponding development projections [27]. Reports regarding Canada, Chile, China, India, and Tunisia also highlight significant investment of these countries through initiative programs, confirming the validity of the projections provided by other investigations [28]. Conversely, analyses of Argentina, Mexico, Russia, Ukraine, and the United Kingdom reveal either declining trends, stability, or volatility largely influenced by financial constraints [33].

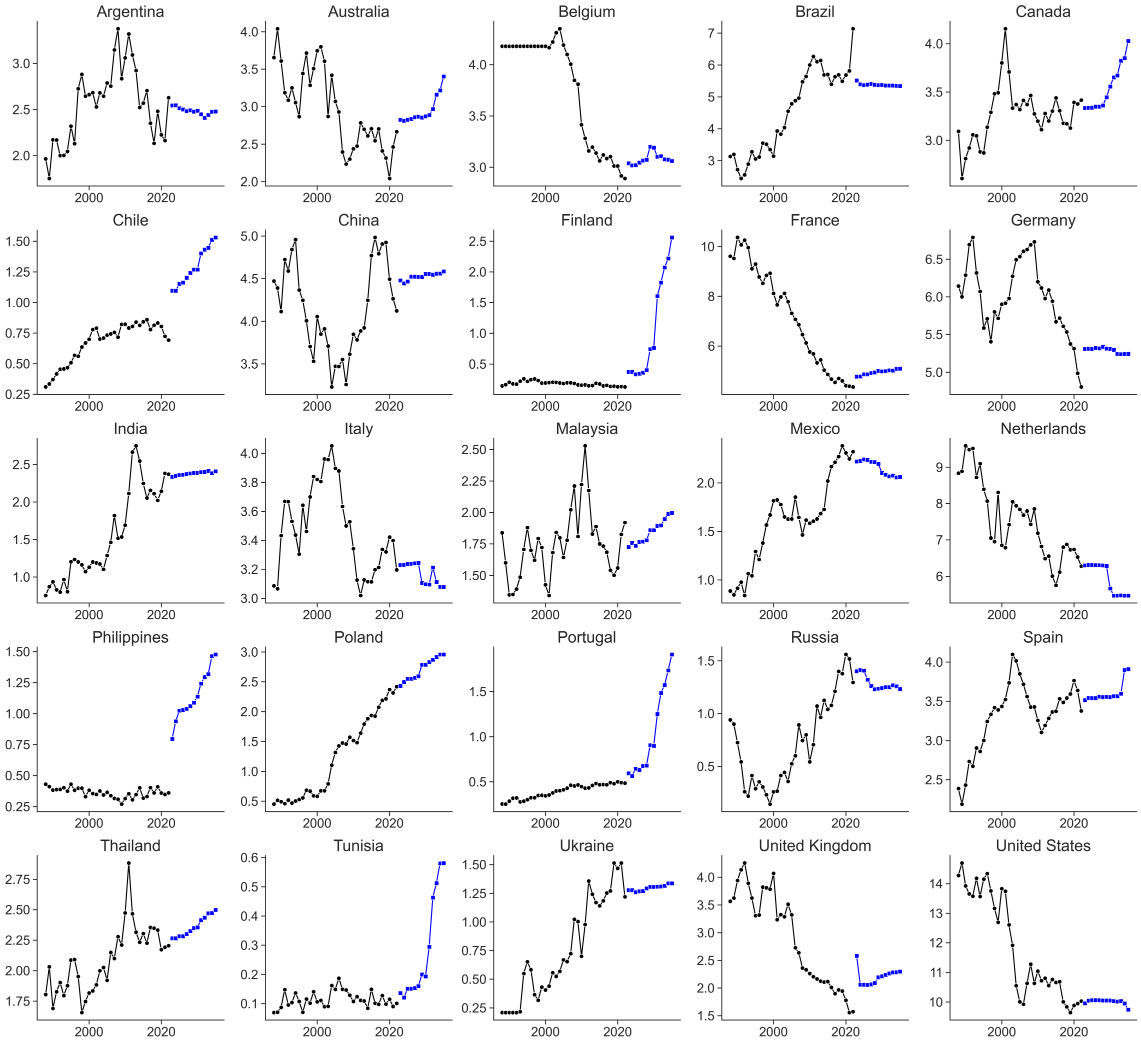

Figure S1. Export Market Share [Percentage] - real vs predicted (1988-2035) across various countries.

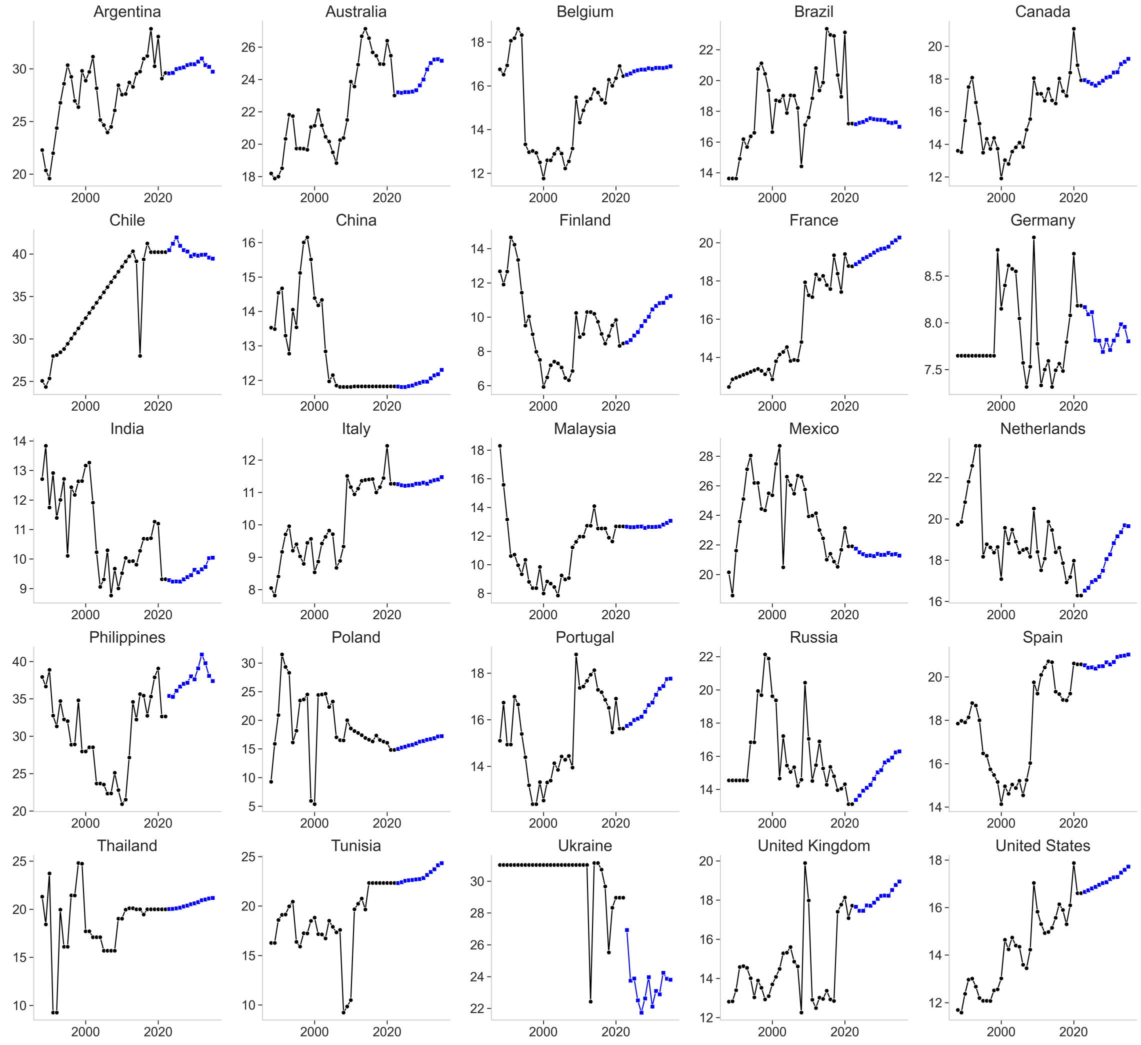

Figure S2. Share of Food, Beverage, and Tobacco Subsector's Value Added within the Total Manufacturing Sector [percentage] - real vs predicted (1988-2035) across various countries.

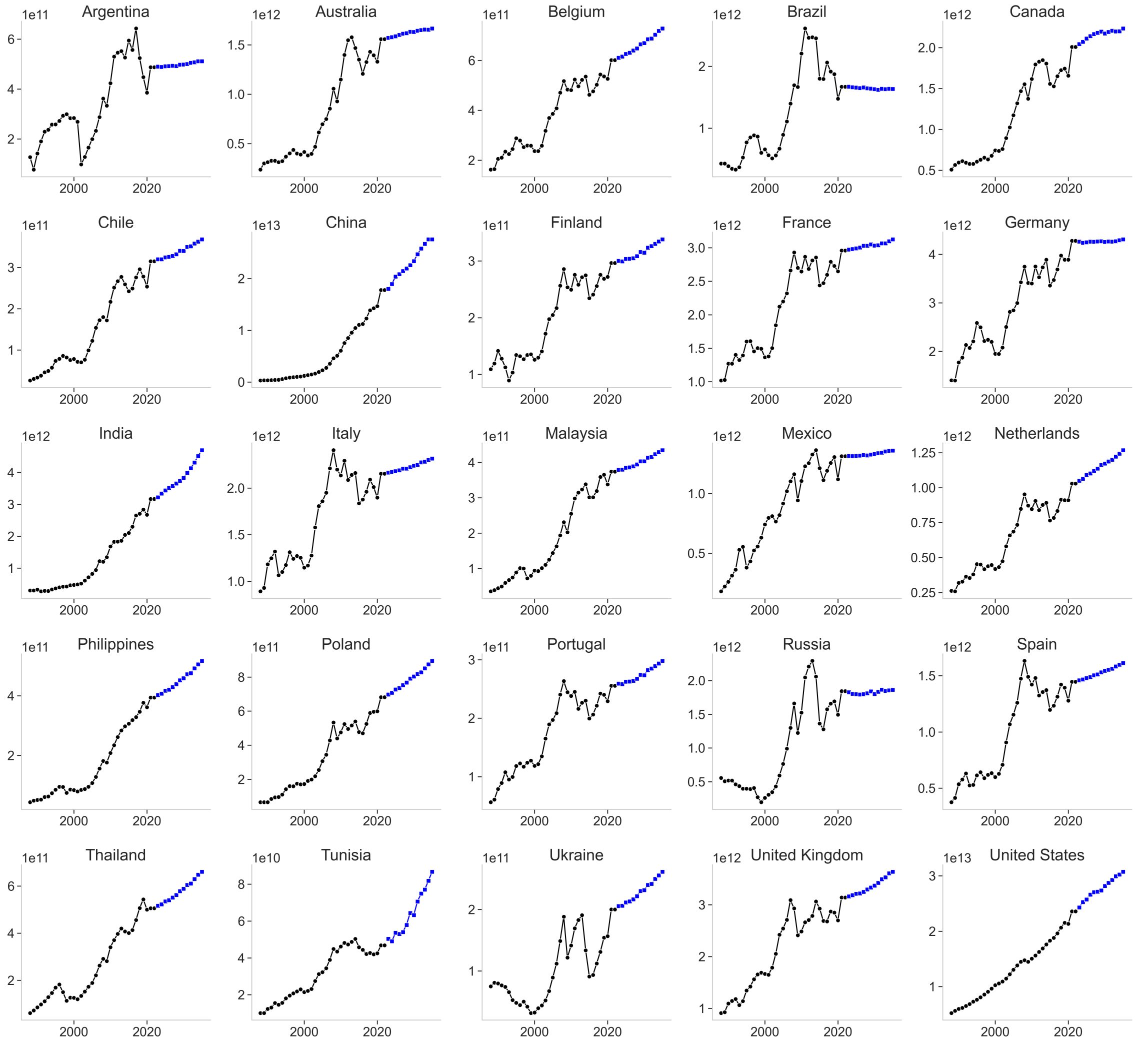

Figure S3. GDP ($ USD) - real vs predicted (1988-2035) across various countries.

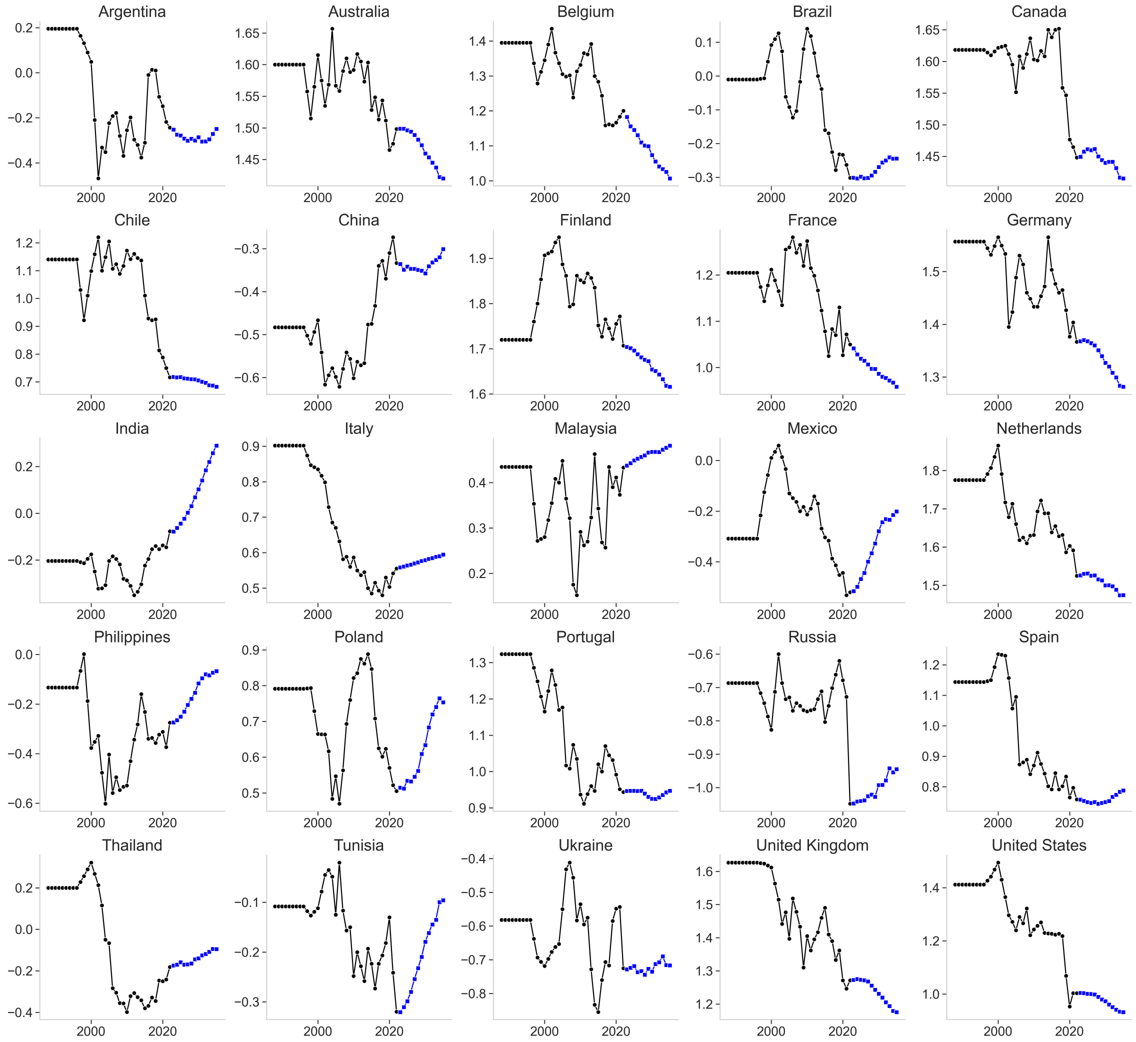

Figure S4. Government Reliability Index - real vs predicted (1988-2035) across various countries.

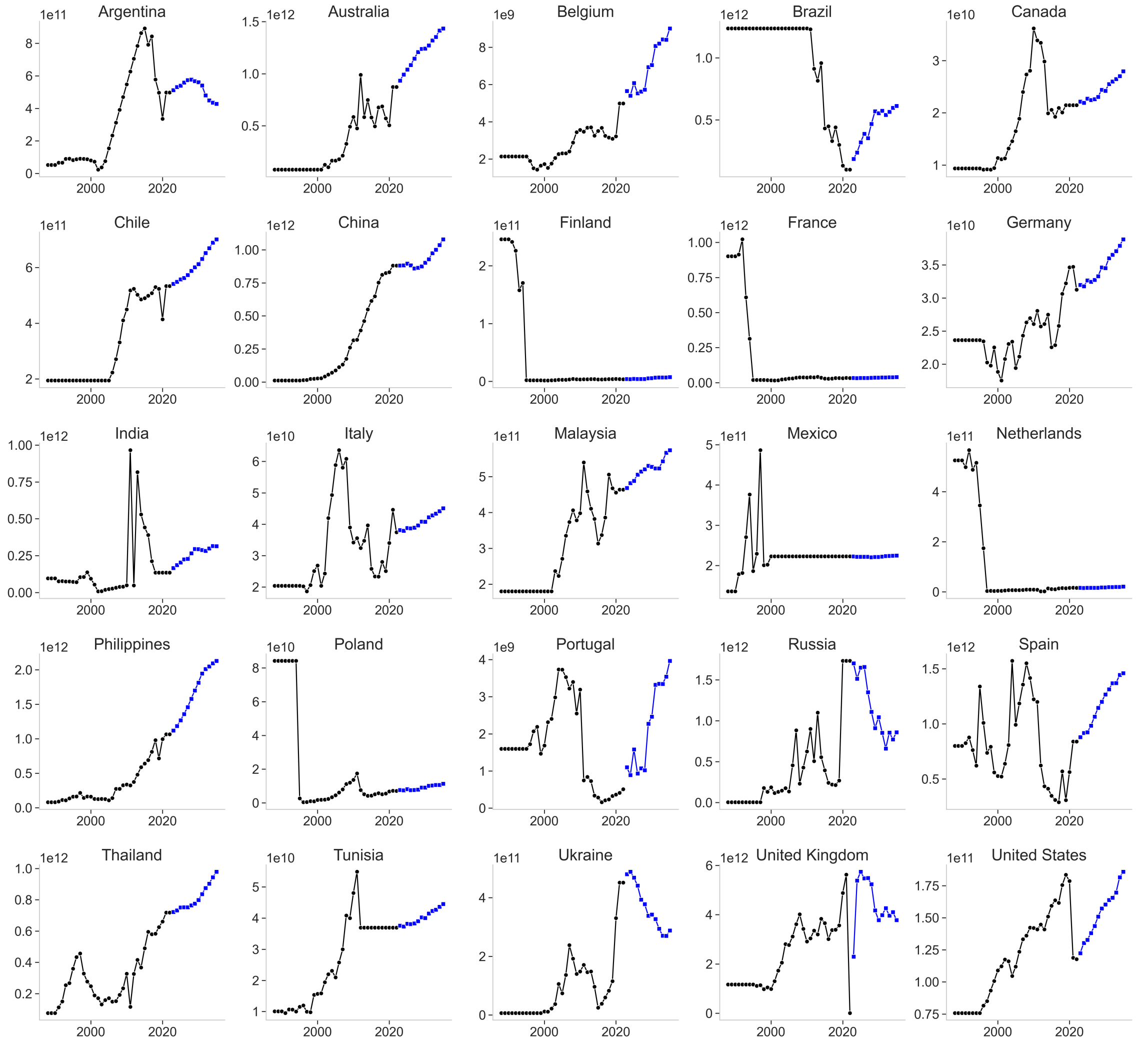

Figure S5. Infrastructure Investment and Maintenance in Transport ($ USD) - real vs predicted (1988-2035) across various countries.

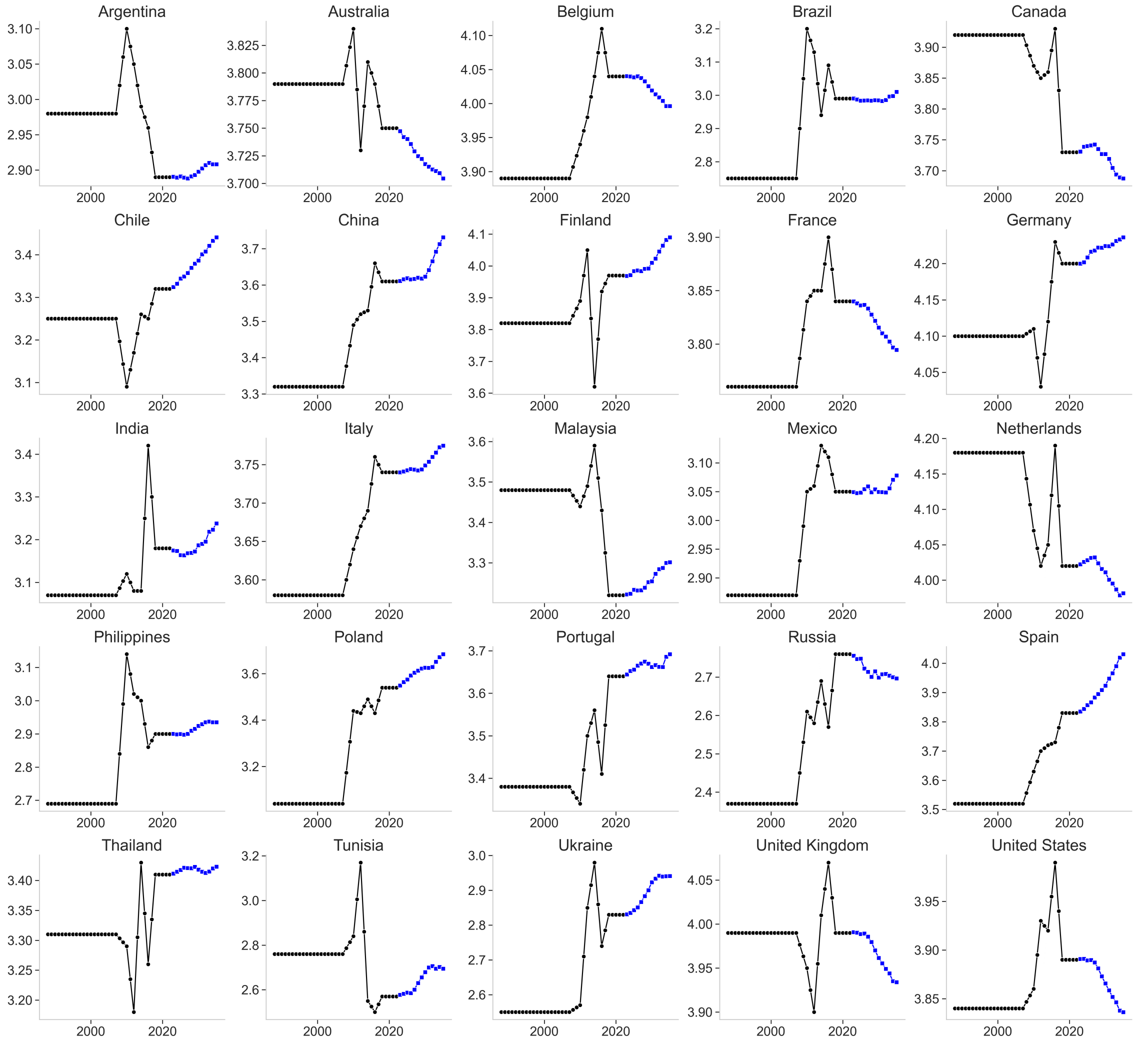

Figure S6. Logistics Performance Index (LPI) - real vs predicted (1988-2035) across various countries.

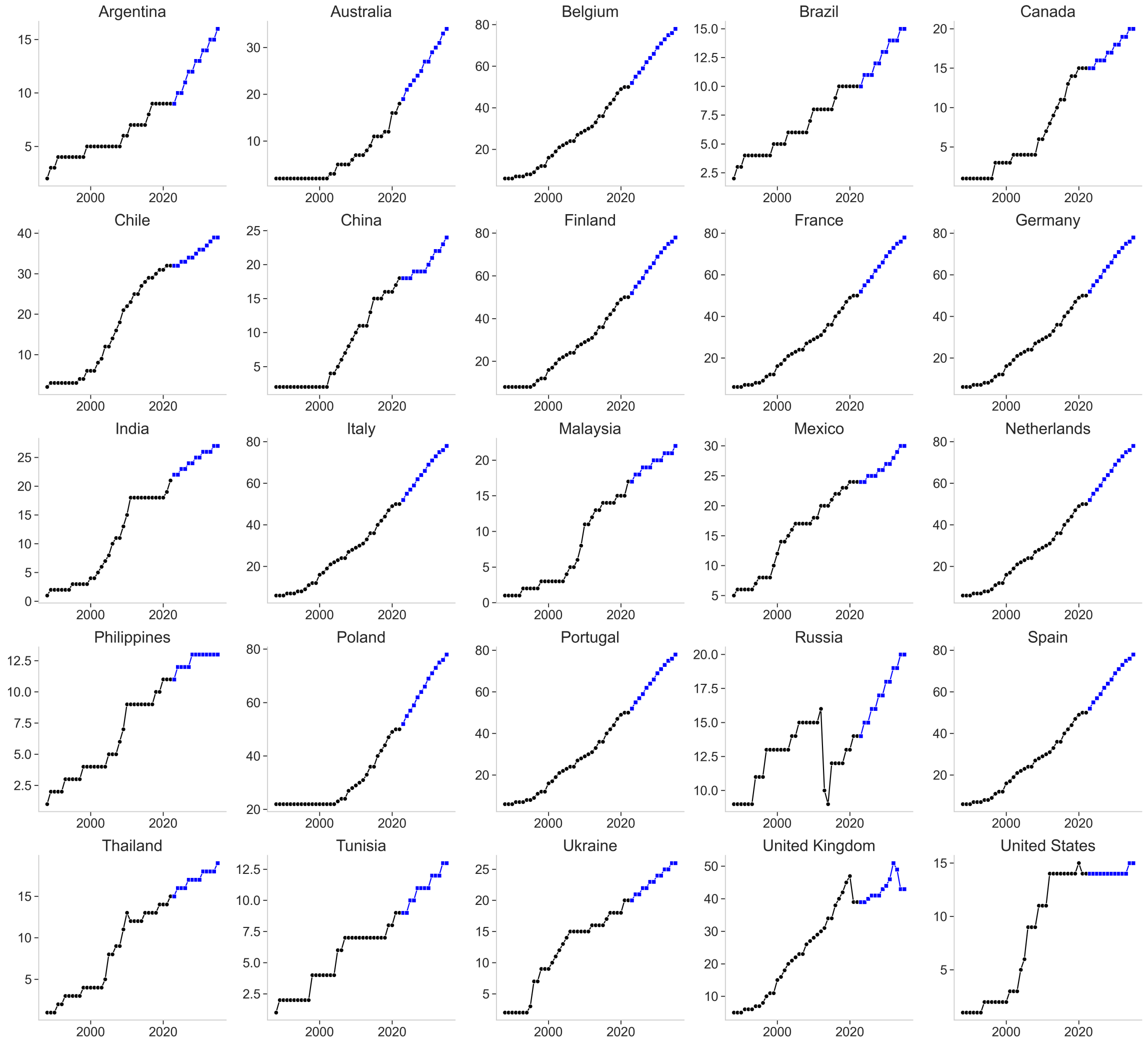

Figure S7. Number of Trade Agreements - real vs predicted (1988-2035) across various countries.

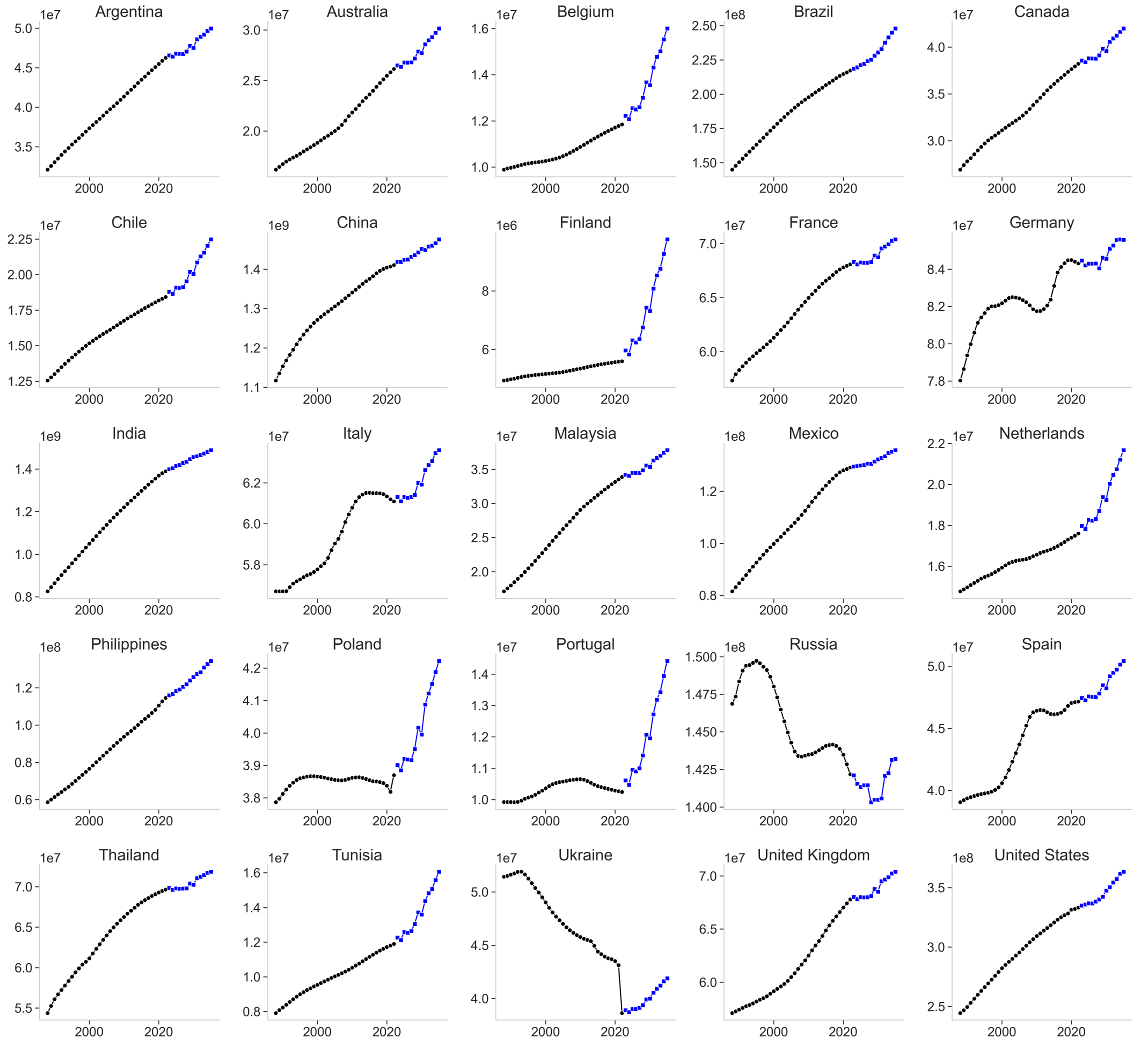

Figure S8. Population - real vs predicted (1988-2035) across various countries.

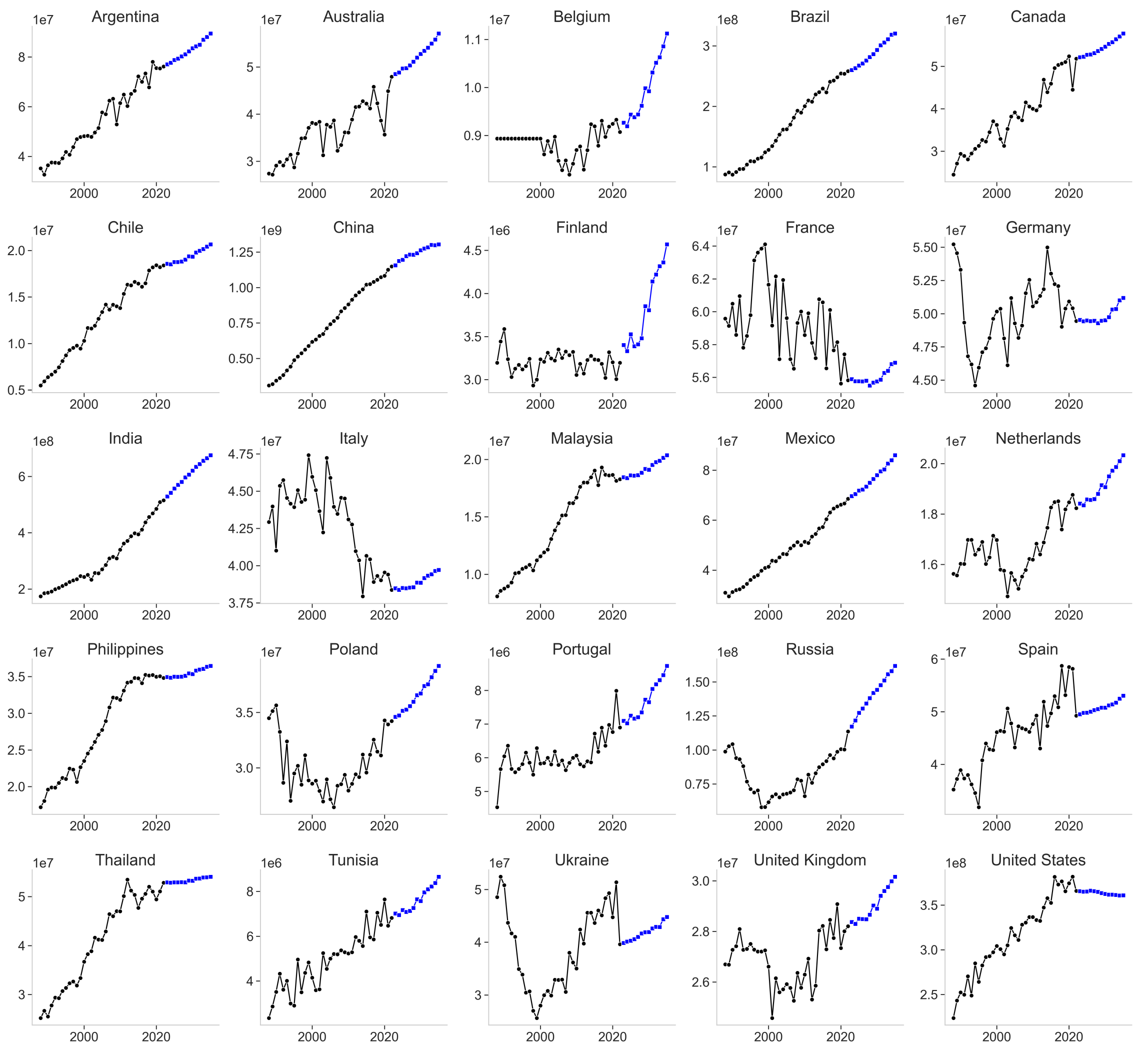

Figure S9. Total Value of Crop, Animal, and Aquaculture Production [Thousand $ USD] - real vs predicted (1988-2035) across various countries.

Table S38. Predicted Export Market Share by country in 2035 (ranked).

Country	Predicted Export Share (%) in 2035	Rank in This Study
United States	9.736	1
Netherlands	5.469	2
Brazil	5.341	3
Germany	5.243	4
France	5.089	5
China	4.586	6
Canada	4.028	7
Spain	3.909	8
Australia	3.403	9
Italy	3.078	10
Belgium	3.060	11
Poland	2.960	12
Finland	2.563	13
Thailand	2.499	14
Argentina	2.480	15
India	2.410	16
United Kingdom	2.300	17
Mexico	2.061	18
Malaysia	1.997	19
Portugal	1.912	20
Chile	1.532	21
Philippines	1.478	22
Ukraine	1.338	23
Russia	1.234	24
Tunisia	0.582	25

Table S39 reports the percentage change in projected Export Market Share in 2035 when individual explanatory variables are excluded from the Random Forest prediction model, relative to the baseline projection that includes the full set of informative predictors. For each predictor, the Random Forest model is retrained without that variable, while all other inputs and forecasting procedures are held fixed. Percentage changes are computed at the country level relative to the baseline 2035 projection, as the difference between the projection obtained with and without the excluded predictor, divided by the baseline projection and expressed in percent. It provides a sensitivity-based assessment of the dependence of long-horizon projections for Export Market Share on individual predictors. Larger absolute percentage changes indicate greater sensitivity of the 2035 projections to the excluded predictor, while smaller changes indicate more limited influence. The sensitivity analysis indicates that the projected 2035 agri-food Export Market Shares exhibit heterogeneous responses to the exclusion of individual predictors across countries. No single explanatory variable systematically dominates the projections for all exporters. Instead, sensitivity is distributed across multiple predictors and varies by country, reflecting differences in structural characteristics and baseline Export Market

Shares. Model fit metrics for the leave-one-out re-estimations are highly similar to those of the baseline Random Forest and are therefore not reported for brevity. In a nutshell, the results suggest that long-horizon projections are not driven mechanically by a single smooth macroeconomic trend, but rather reflect the joint contribution of multiple structural predictors.

Table S39. Sensitivity of 2035 agri-food Export Market Share projections to the exclusion of individual explanatory variables. Each column corresponds to a model re-estimate without the indicated explanatory variable. Entries report the percentage change (%) in projected 2035 Export Market Shares relative to the baseline forecast that includes all explanatory variables. Negative values indicate lower projected Export Market Shares relative to the baseline when the corresponding variable is excluded.

	**Logistics Performance Index (LPI)**	**Number of Trade Agreements**	**Population**	**Total Value of Crop, Animal, and Aquaculture Production**	**Government Reliability Index**	**GDP**	**Share of Food, Beverage, and Tobacco Subsector's Value Added within the Total Manufacturing Sector**	**Infrastructure Maintenance and Investment in Transport**
Argentina	4.82	-0.86	-3.78	-6.78	-2.29	-2.60	-2.38	-1.24
Australia	0.73	-1.93	-4.38	-18.36	-12.72	-12.77	-9.86	-11.00
Belgium	-0.47	1.49	0.02	2.06	1.83	-0.73	0.48	-1.96
Brazil	1.99	2.88	0.73	2.22	3.14	6.27	5.95	4.85
Canada	1.90	-5.71	-5.82	-18.04	-13.05	-8.11	-5.52	-9.10
Chile	12.29	4.14	6.63	15.02	7.37	-19.37	23.53	14.65
Germany	-0.07	-2.21	0.51	-3.81	1.81	-0.78	-3.21	-2.37
Finland	-38.36	13.25	12.07	31.60	-4.15	7.09	16.83	18.08
France	-12.97	-0.11	1.28	-1.61	3.42	-1.56	-3.34	-6.47
India	7.09	1.55	-2.43	0.21	-1.43	-5.50	3.34	-2.06
Italy	-5.91	2.39	6.80	4.69	-1.11	-0.44	-7.28	-14.02
Malaysia	19.08	1.77	1.01	5.04	-0.74	-3.85	-6.57	-8.58
Mexico	4.57	-2.38	-0.63	-3.00	7.70	-1.22	-3.36	-1.86
Netherlands	14.97	-2.59	1.33	-4.01	-0.27	-4.02	-0.46	-3.40
China	-4.33	0.04	-3.32	-1.92	-0.95	-3.36	-4.14	-3.18
Philippines	1.01	-0.02	2.54	1.17	-21.64	-12.78	-2.49	-14.78
Poland	-12.13	0.28	-2.87	0.31	-0.76	7.12	-0.76	-0.82
Portugal	-44.42	5.97	12.25	-49.58	19.41	33.21	10.31	-0.46
Russia	-1.48	4.67	7.73	-18.10	1.08	-9.92	0.47	3.95
Spain	-13.40	1.11	0.67	-5.83	3.95	3.51	-2.56	-2.52
Thailand	-5.17	-0.60	1.33	-3.26	-5.30	-8.44	-0.92	-5.03
Tunisia	46.10	7.36	-19.53	-4.84	0.73	-13.70	16.46	8.02
Ukraine	-3.09	2.39	-2.73	-3.06	6.71	13.34	-3.22	-3.66
United Kingdom	-11.00	-3.64	-5.72	-2.62	-8.26	-16.31	-5.74	-8.69
United States	2.66	1.61	1.23	0.99	1.25	1.01	3.21	1.91

Regional supply concentrations

The Shannon entropy index $H(c,t)$ ranges from 0, indicating no diversity where all exports are concentrated in a single region, to $\log_2(N)$, representing maximum diversity where exports are evenly distributed across all trading partners. Based on the classification of world regions by the Food and Agriculture Organization of the United Nations (FAO) [109], our study generally includes $N = 22$ regions, resulting in a possible, maximum entropy value of $\log_2(22) \approx 4.4594$. Accordingly, we define three levels of export market diversity using equal intervals within this range: low diversity when $H(c,t) \leq 1.4865$, medium diversity when $1.4865 < H(c,t) \leq 2.9729$, and high diversity when $2.9729 < H(c,t) \leq 4.4594$. These thresholds provide a consistent and interpretable basis for assessing the extent of trade diversification across countries and over time, considering the number of trading partners as an influential factor in diversification. The historical Shannon entropy index for all nations is depicted in Figure S10.

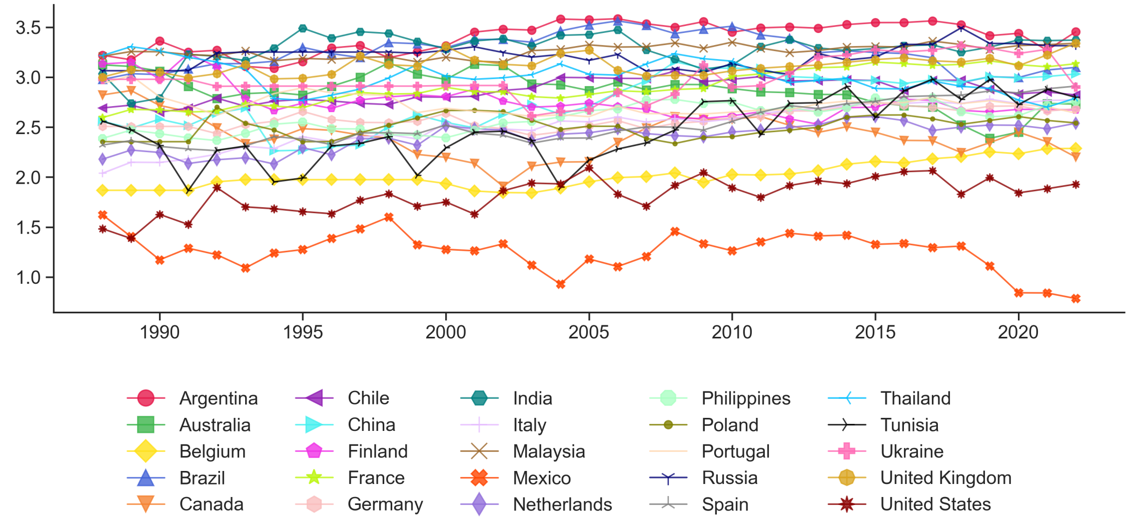

Figure S10. Shannon entropy index across various countries from 1988 to 2022.

Countries with highly concentrated exports (low diversification)

Mexico demonstrates a clear dependency on a single, dominant region for its exports, revealing potential vulnerabilities in its trade structures. More specifically, about 80% of

Mexico's historical exports go to Northern America, largely driven by its close integration with the United States and Canada through agreements. While this proximity and trade partnership facilitate efficiency and reduced logistics costs, they also render Mexico highly vulnerable to these countries' trade policy shifts. According to the Figure S11 illustrating Mexico's export share over the period, Northern America has shown a steady and gradual increase in its share. In contrast, exports to Central America and Western Europe have clearly declined.

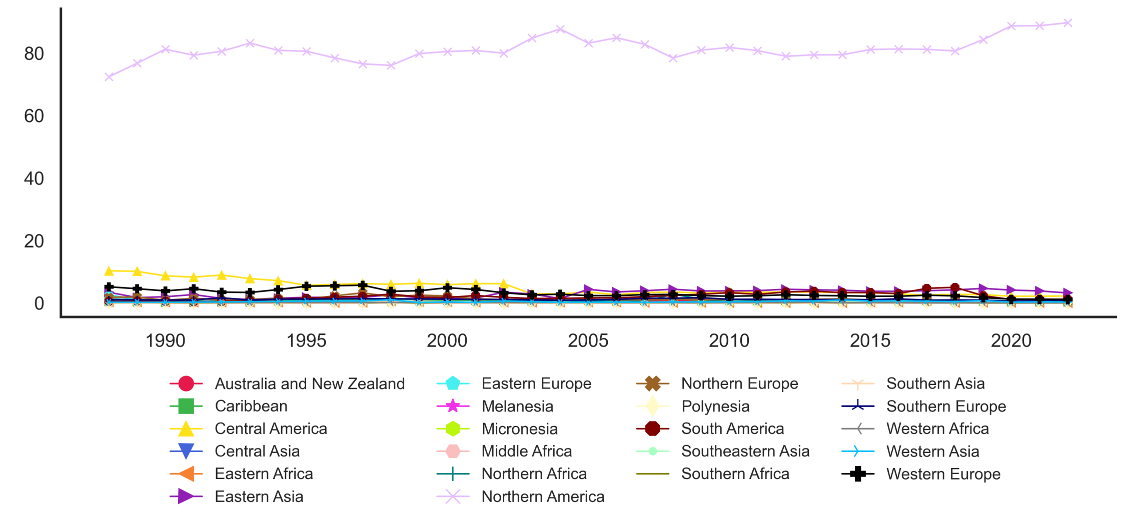

Figure S11. Evolving regional export shares [Percentage] of Mexico (1988-2022).

Mid-level export diversification

Some countries lie between the two extremes, showing moderate diversification. For instance, the United States' export patterns are characterized by relatively regional concentration, with notable regional concentration in Africa alongside slight expansion to other parts of the world. 55.76% is directed toward Northern Africa, positioning the United States as a major agri-food trade partner in the region. Complementary export flows to Eastern Africa (7.7%), Southern Africa (6.54%), Middle Africa (4.18%) further highlight this regional integration across Africa markets. Cumulatively, the United States directs over 74% of its agri-food exports to the African continent. The remaining export distribution, particularly to Australia and New Zealand (22.95%), demonstrates

that the United States also maintains selective global linkages, especially in high-income, politically stable regions. While this dual orientation can confer a very low level of resilience, the high dependence on a single region (Northern Africa) exposes the United States to concentrated geopolitical and economic risks. Disruptions in food subsidy programs, civil unrest, or import policy changes could significantly affect trade stability. From a policy standpoint, expanding commercial ties to Latin America, Asia, or Europe may enhance strategic flexibility and hedge against region-specific vulnerabilities, especially in light of increasing global food insecurity and supply chain shocks. Moreover, United States export shares to Northern Africa have declined over the past 35 years, while exports to Australia, New Zealand, and Middle Africa have increased, as shown in the Figure S12.

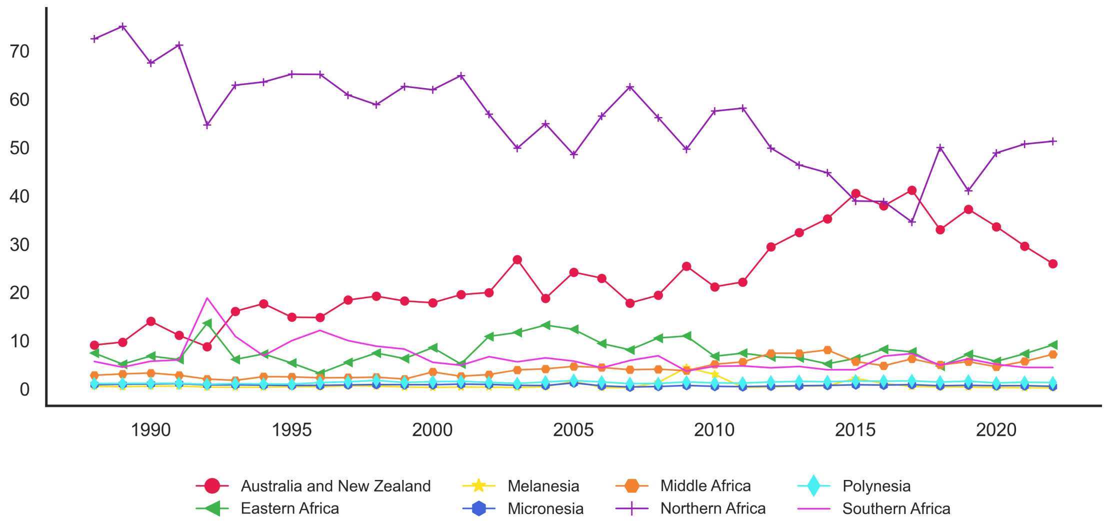

Figure S12. Evolving regional export shares [Percentage] of the United States (1988-2022).

Belgium displays a significant export reliance on Western Europe, accounting for nearly 63.49% of its exports. Belgium also directs approximately 12.68% of its exports to Northern Europe, highlighting a notable, though secondary, regional trade connection beyond its dominant ties within Western Europe. As a core European Union (EU) economy, Belgium's trade pattern reflects the highly integrated intra-EU trade network. However, this regional concentration means external demand fluctuations in Western and Northern Europe (e.g., Brexit-driven changes or countries' economic slowdowns) can

disproportionately affect Belgium. Based on the Figure S13, the overwhelming dominance of Western Europe in Belgium's export share shows a consistent and significant downward trend from the mid-2000s. Eastern Europe also demonstrates a modest but steady increase in its share. Other regions, while individually smaller, generally maintain low and stable export shares, with minor fluctuations over time.

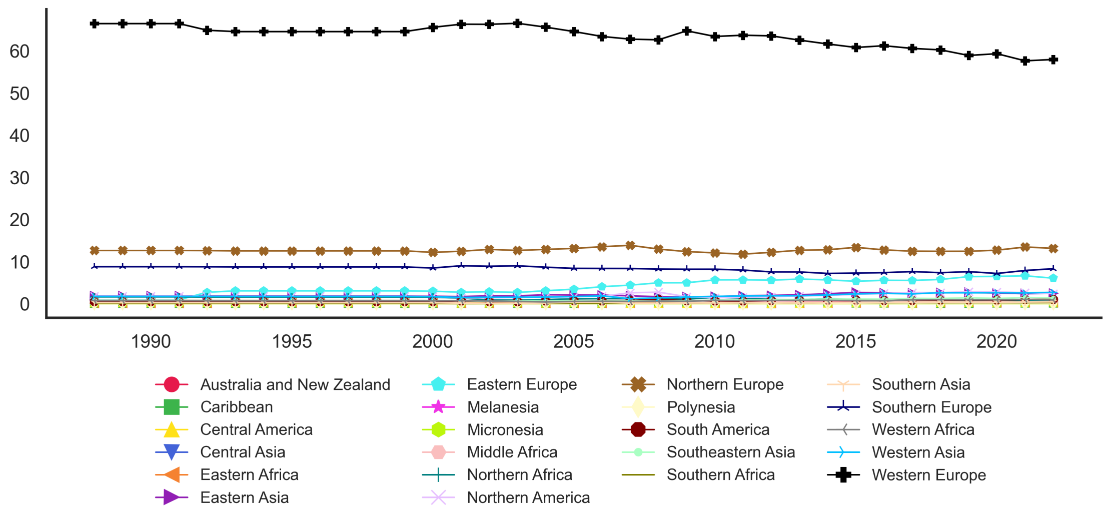

Figure S13. Evolving regional export shares [Percentage] of Belgium (1988-2022).

The Netherlands exhibits a partly regionally concentrated export structure, with over half (50.86%) of its exports directed toward Western Europe. This heavy reliance reflects its deep integration within the EU's single market, facilitated by geographical proximity, shared infrastructure, and harmonized regulatory frameworks. As a result, the Netherlands benefits from efficient trade logistics and intra-EU economic synergies, particularly through its role as a logistics hub via the Port of Rotterdam. Beyond this dominant export partner, the Netherlands also maintains moderate export shares to other European subregions, notably Northern Europe (16.39%), Southern Europe (11.91%), and to a lesser extent, Eastern Europe (5.12%). Collectively, nearly 84% of Dutch exports are concentrated within Europe, underscoring the country's identity as a regionally focused exporter. Its concentration introduces exposure to region-specific shocks. For example, economic stagnation in the Eurozone, energy crises, or political disruptions such as post-Brexit trade frictions could disproportionately impact the Netherlands' export

performance. Its limited engagement with fast-growing emerging markets, particularly in Asia, Africa, and Latin America, may represent a missed opportunity for hedging regional risk and capitalizing on global demand shifts. Pertaining to the Figure S14, the Netherlands' export profile underwent a persistent reduction in market shares for Western and Southern Europe during the period. Conversely, a sustained expansion is discernible in exports directed towards Eastern Europe, signifying its burgeoning importance as a trade partner. Likewise, Northern Europe and Eastern Asia also recorded a consistent, albeit more gradual, increase in their share. Other regional destinations typically maintained marginal and stable export contributions, exhibiting only subtle variations across the years.

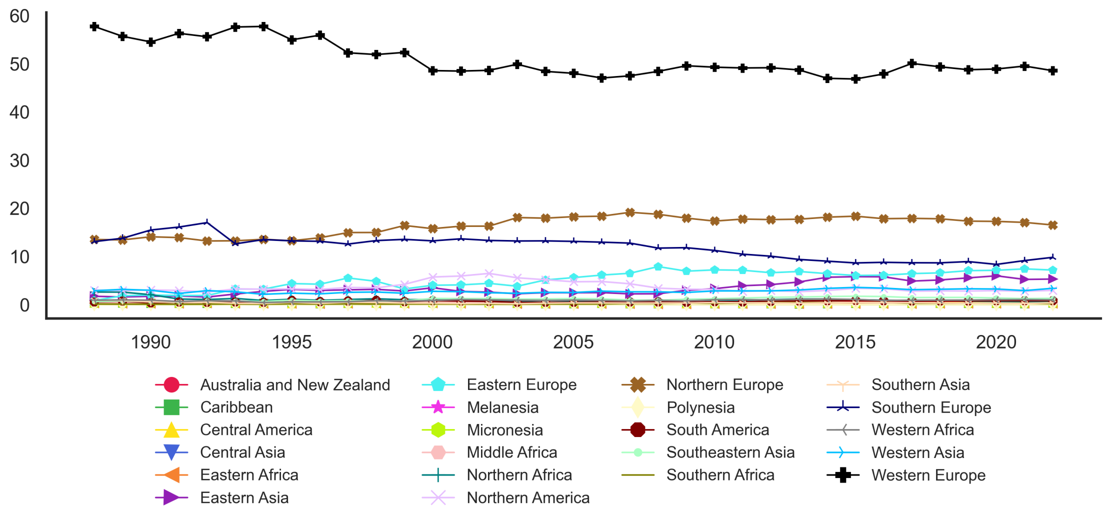

Figure S14. Evolving regional export shares [Percentage] of the Netherlands (1988-2022).

Canada exports predominantly to Northern America, again due to its trade ties with the United States and Mexico. This results in roughly 52.9% of export dependency, suggesting limited diversification outside its regional trade bloc. Canada's next major food trade partners after Northern America are countries in Eastern Asia, accounting for approximately 19.4% of its agri-food exports. This heavy reliance on just two regions suggests that concurrent natural, economic, or political disruptions in either could severely impact Canada's agri-food export performance. Recent United States tariff implementations highlight how such dependency can quickly become a strategic risk. In accordance with the Figure S15, Canada's market share directed towards North America

has experienced a gradual expansion overall, notwithstanding a pronounced reduction observed between the early 2000s and early 2010s. Conversely, the aggregate export shares allocated to both Eastern Asia and Eastern Europe have demonstrably declined. Other regional export destinations typically maintain relatively modest and stable contributions, exhibiting only minor fluctuations throughout the years.

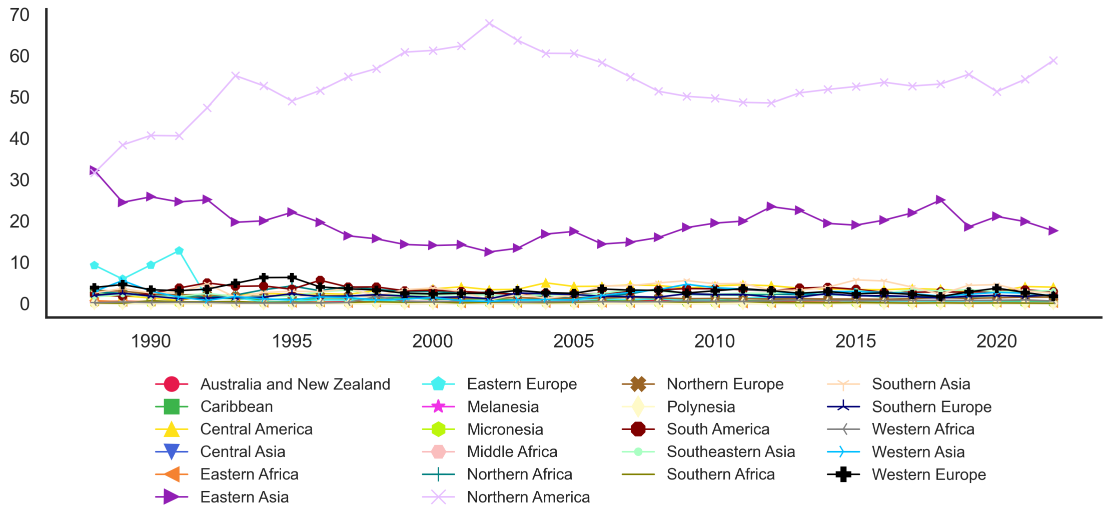

Figure S15. Evolving regional export shares [Percentage] of Canada (1988-2022).

Tunisia's agri-food export profile is partially globalized. Notably, about 37.98% of its exports are directed toward Southern Europe, followed by Northern Africa (22.29%), and Western Europe (18.52%). Additionally, Northern America accounts for a modest but significant share (5.58%). Concentrating exports in Southern Europe makes Tunisia vulnerable; if that region faces economic or political problems, Tunisian exporters could be hit hard. Similarly, Tunisia's close integration with Northern African markets may expose it to regional spillover risks tied to political unrest or fragile institutions in neighboring countries. For policymakers, the findings underscore the importance of strategically expanding trade relationships beyond the Mediterranean region, which could help Tunisia reduce vulnerability and build a more resilient agri-food trade strategy in the face of global uncertainty. Tunisia's export shares fluctuate considerably across most regions over time. Given that and Figure S16, exports to Northern Africa and Northern America have increased, while those to Western and Southern Europe have declined. Other regions

generally contribute small, consistent export shares with minimal changes throughout the period.

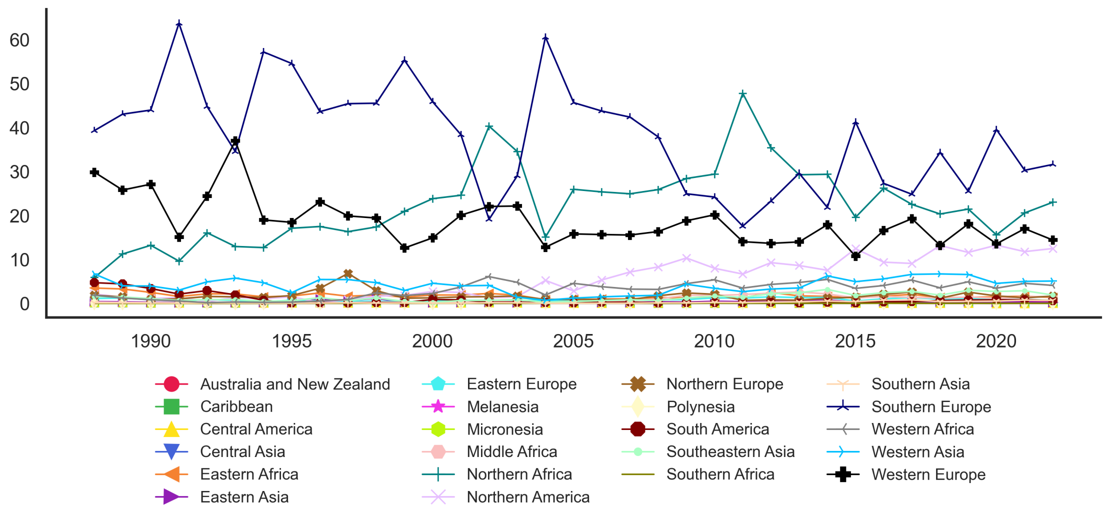

Figure S16. Evolving regional export shares [Percentage] of Tunisia (1988-2022).

Poland demonstrates a moderately regional export profile, with a substantial share (38.85%) directed toward Western Europe. However, rather than being overwhelmingly dependent on a single subregion, Poland distributes its exports across multiple parts of Europe, including Eastern Europe (22.31%), Northern Europe (14.64%), and Southern Europe (11.22%). This distribution across four distinct European subregions suggests a degree of diversification within its regional sphere. Collectively, over 87% of Poland's exports remain within the broader European region, indicating that while it is not heavily global, it is not narrowly regional either. The Figure S17 shows that Poland's export share over time has risen in Northern and Eastern Europe but reduced in Southern and Western Europe.

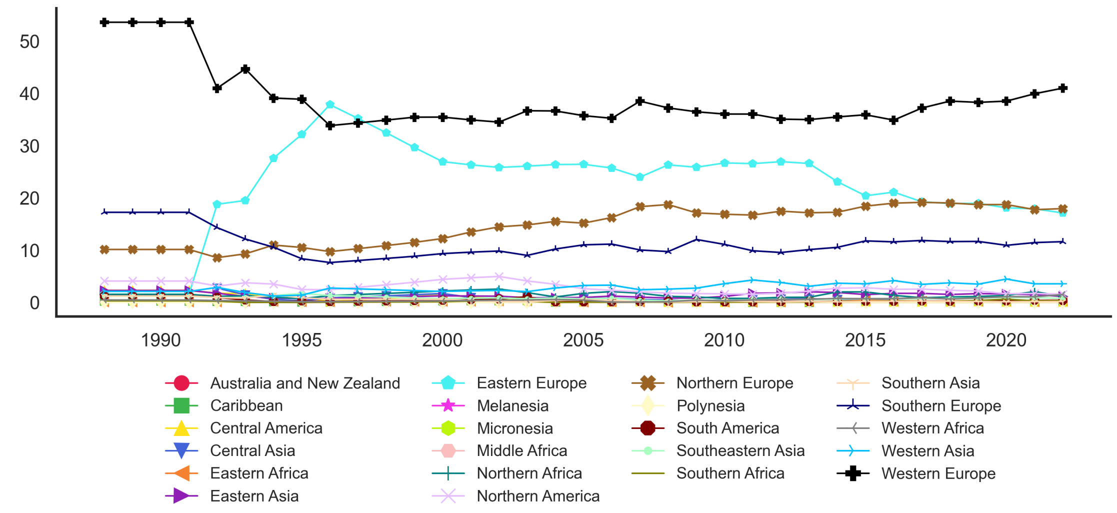

Figure S17. Evolving regional export shares [Percentage] of Poland (1988-2022).

Italy demonstrates a relatively regional orientation in its agri-food export structure, with Western Europe accounting for a dominant 49.26% of its exports. This reflects Italy's deep integration into the EU single market and its reliance on neighboring countries for trade. Additional strong regional ties are visible through significant exports to Northern Europe (12.96%), Eastern Europe (5.63%), and Southern Europe (8.39%). Collectively, over 76% of Italy's agri-food exports are concentrated within Europe, underscoring its regional interconnectedness and close economic ties across the continent. At the same time, Italy demonstrates limited global involvement, particularly with Northern America (10.74%), which represents a substantial non-European trade partner, indicating that while Europe remains the central trade hub, Italy does maintain a subtle presence in other strategic regions. This strategy results in minimal diversification, but the country's substantial dependence on European markets, especially Western Europe, still introduces vulnerability to region-specific shocks. Taking into consideration the Figure S18, Italy's export share to Western Europe has experienced a substantial reduction in its share, despite remaining a dominant partner. Conversely, both Eastern and Southern Europe, as well as Northern America, exhibit a gradual upward growth.

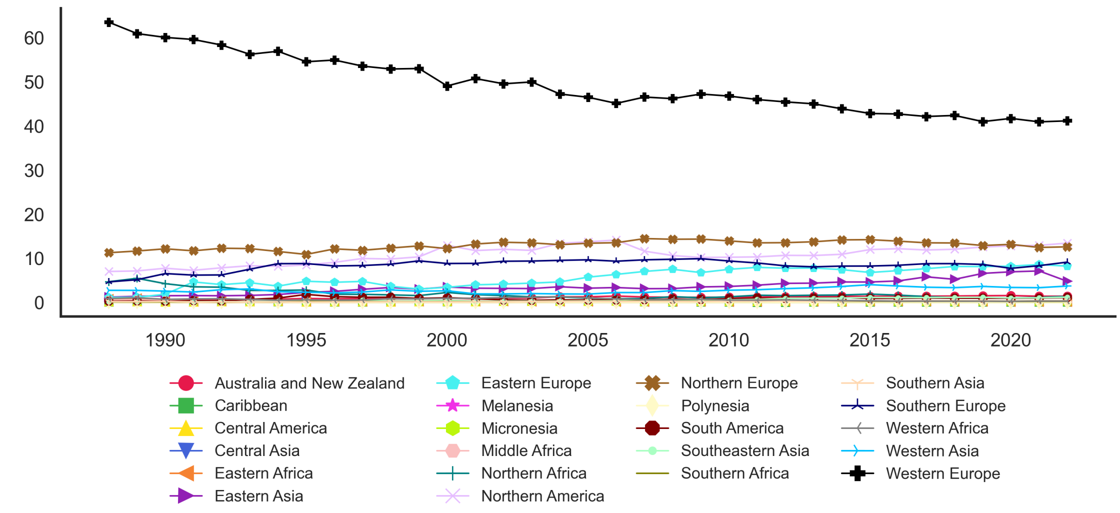

Figure S18. Evolving regional export shares [Percentage] of Italy (1988-2022).

Spain also presents a moderately regional export profile. 43.76% is directed toward Western Europe, followed by substantial shares to other European subregions, including Southern Europe (20.82%), Northern Europe (13.45%), and Eastern Europe (4.47%). Collectively, around 83% of Spain's exports remain within Europe, signaling a strong regional interconnectedness. The 35-year export data of Figure S19 for Spain reveals a notable decline in Western Europe's market share, despite its continued dominance. In contrast, Eastern Europe and Eastern Asia show steady growth, reflecting their rising importance in Spain's trade network. Other regions maintain small, stable export shares with minimal changes over time.

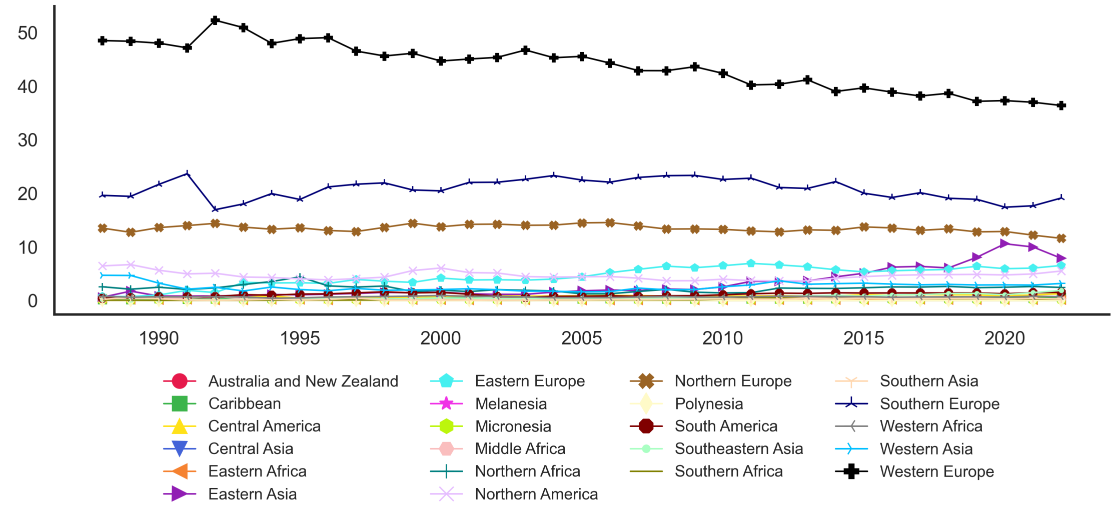

Figure S19. Evolving regional export shares [Percentage] of Spain (1988-2022).

Similarly, Germany presents a moderately regional export profile. While Western Europe absorbs the largest single share (39.7%), substantial volumes also flow to Southern Europe (18.92%), Eastern Europe (12.56%), and Northern Europe (14.63%). This dispersion across four distinct European sub-regions signals a balanced intra-European footprint, yet it still leaves roughly 86% of German exports anchored within the continent. This marks Germany as neither narrowly regional nor fully global in its trade orientation. Looking at the Figure S20, Germany's export shares to Western and Southern Europe have slightly decreased. Conversely, Eastern Europe exhibits a dramatic surge in its share. Other regions typically represent smaller and more consistent export shares, demonstrating only minor variations across the years.

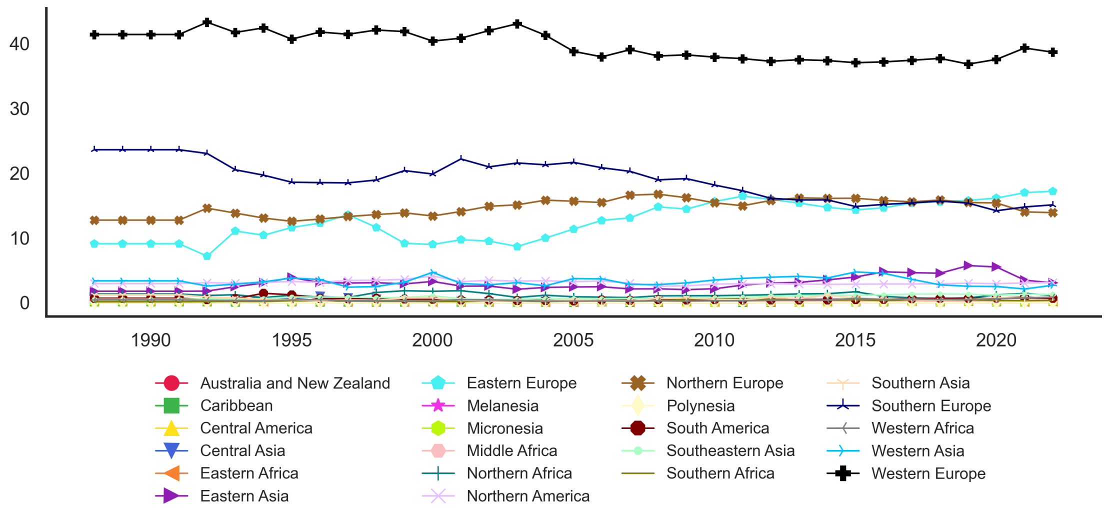

Figure S20. Evolving regional export shares [Percentage] of Germany (1988-2022).

The Philippines demonstrates a relatively diversified agri-food export structure, with leading destinations including Eastern Asia (30.64%), Northern America (27.97%), Western Europe (15.79%), and Southeastern Asia (11.15%). This diversified profile points to robust connections both regionally and globally. Furthermore, strong trade relations with Eastern and Southeastern Asia are supported by geographic proximity and deepening economic integration. The Figure S21 shows clear changes in the Philippines' export profile over time. A prominent trend is a gradual fall in market share for Northern America and Western Europe. Conversely, Southeastern Asia exhibits a sustained and significant expansion in its export share, signaling a deepening of regional trade ties. Despite fluctuations, Eastern Asia rose slightly during the duration.

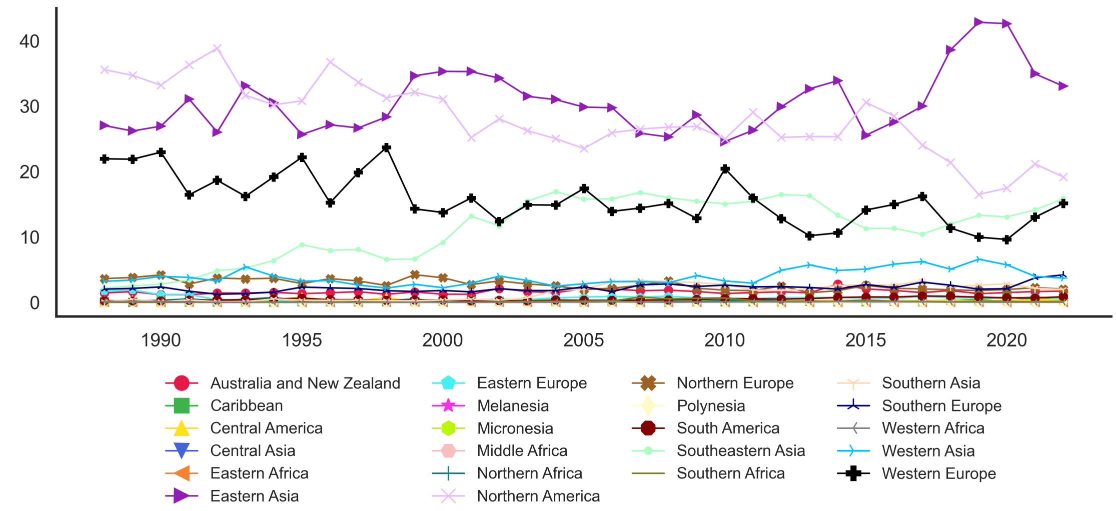

Figure S21. Evolving regional export shares [Percentage] of the Philippines (1988-2022).

Portugal has agri-food exports with a relative diversification, with major flows to Southern Europe (31.6%), Western Europe (27.19%), Northern Europe (11.23%), and Middle Africa (9.83%). Its strong presence in Europe reflects the advantages of EU integration, such as seamless regulations and efficient logistics. This diversification enhances resilience. To strengthen its position, Portugal can expand into stable regions of America and Asia. The Figure S22 highlights the substantial and persistent reduction in Portugal's export share for Western Europe, which, despite its historical dominance, saw a considerable decrease in its contribution. Similarly, Northern Europe and North America also experienced a tangible drop in their respective export proportions. In contrast, exports to Southern Europe steadily grew, making it a more important destination. Furthermore, South America and West Asia demonstrated slow, modest growth in their shares. Other regions kept small, steady export shares with minor changes.

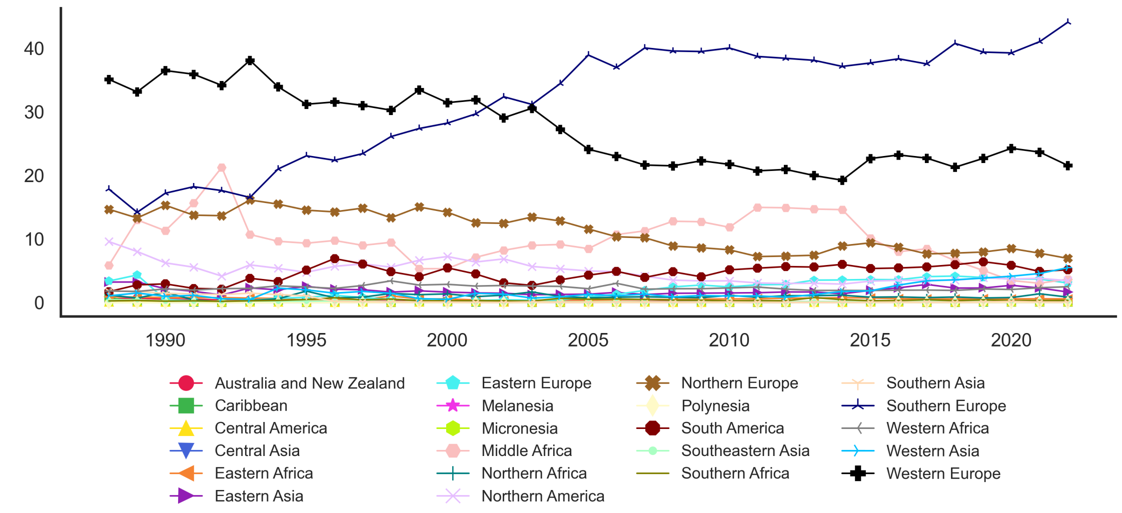

Figure S22. Evolving regional export shares [Percentage] of Portugal (1988-2022).

China's agri-food export portfolio is regionally concentrated yet globally active, with major flows to Eastern Asia (45.04%), Southeastern Asia (16.23%), Eastern Europe (5.22%), Western Europe (7.42%), and Northern America (7.45%). This structure reflects China's strong intra-Asian integration and deep trade networks with both emerging and advanced economies. High dependence on Eastern Asia signals logistical efficiency but increases exposure to regional market fluctuations or geopolitical tensions. Exports to Europe and North America indicate diversification into high-value markets. However, engagement with Africa and Latin America remains limited. To strengthen resilience, China could enhance its presence in underrepresented regions. Pertaining to the Figure S25, China's export profile demonstrates significant reconfigurations across the period to become more diverse. The most prominent trends are the substantial and consistent expansion of market shares directed towards North America and Southeastern Asia, solidifying their position as primary export destinations. Conversely, Eastern Asia, despite its proximity, and Eastern Europe have experienced a discernible downturn in their export shares over the same timeframe.

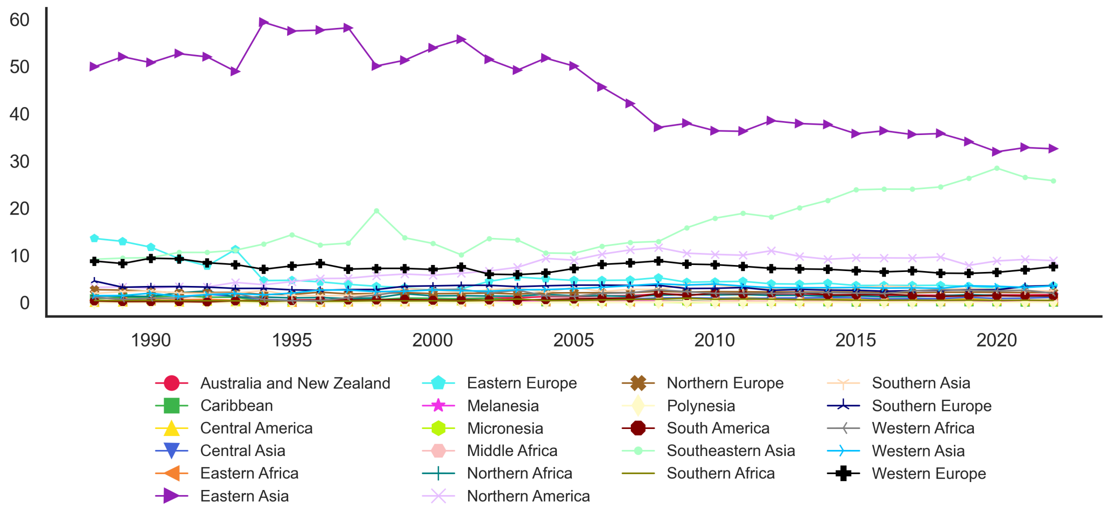

Figure S23. Evolving regional export shares [Percentage] of China (1988-2022).

Finland's agri-food export structure shows pretty global diversification, with major destinations including Northern Europe (30.04%), Eastern Europe (20.05%), Eastern Asia (16.37%), and Western Europe (13.51%). Northern America also accounts for a notable share (5.71%). This concentration in Europe reflects Finland's regional integration within the EU and neighboring economies, offering stability and streamlined trade logistics. The significant share of Eastern Asia signals growing outreach to high-demand Asian markets, though this remains secondary to Europe. While this structure supports trade efficiency, it also increases exposure to EU market dependencies. To enhance resilience, Finland could strengthen ties with underrepresented regions in Africa and Asia. In line with the Figure S24, Finland's export landscape has undergone a significant transformation over the depicted period. The most striking development is the dramatic and sustained surge in export shares directed towards Northern Europe, showcasing a profound reorientation of trade. This contrasts sharply with the substantial decline observed in export shares to Northern America. Moreover, reliance on Southern Europe for agricultural exports has slightly declined. Furthermore, Eastern Europe has also experienced a noticeable downturn in its share of Finnish exports. Conversely, Southeastern Asia exhibits a gradual upward trajectory, indicating its growing importance as a trade partner. Other regions hold smaller, stable export shares with minor changes.

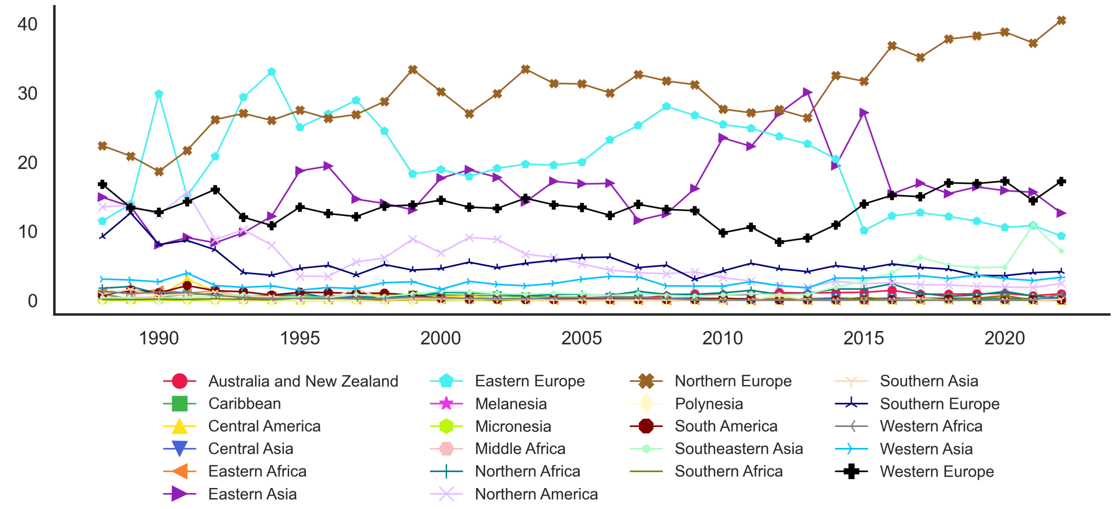

Figure S24. Evolving regional export shares [Percentage] of Finland (1988-2022).

Chile partly exhibits a globally diversified agri-food export pattern, with key markets in Northern America (29.12%), Eastern Asia (15.08%), South America (18.13%), Western Europe (12.02%), Northern Europe (8.2%), and Central America (6.08%). This balanced mix across the Americas, Asia, and Europe enhances Chile's resilience to regional shocks. While intra-regional trade in South America and Central America adds logistical advantages, high exposure to Northern America creates potential vulnerability to the United States' tariff-related trade policy changes. Moreover, Chile's relatively lower presence in Africa and South Asia suggests room for growth. In line with the Figure S25, Chile's export dynamics exhibit a profound reorientation over the depicted period. The most striking trend is the significant and sustained ascent of Eastern Asia's share, particularly from the late 1990s, solidifying its position as Chile's predominant export destination. Concurrently, Central America has shown a rise in its share despite a decline starting in the mid-2000s. Conversely, a clear and consistent downturn is observable in the export shares directed towards traditional markets in Western Europe and Western Asia, reflecting a diminishing reliance on these destinations. Similarly, North America experienced a noticeable and steady decrease in its overall export share throughout the timeframe. South America, while retaining a considerable, albeit fluctuating, share, has generally seen a gradual contraction from its peaks in the mid-1990s. Other regions typically accounted for smaller and more stable portions of Chile's export portfolio,

exhibiting only minor fluctuations.

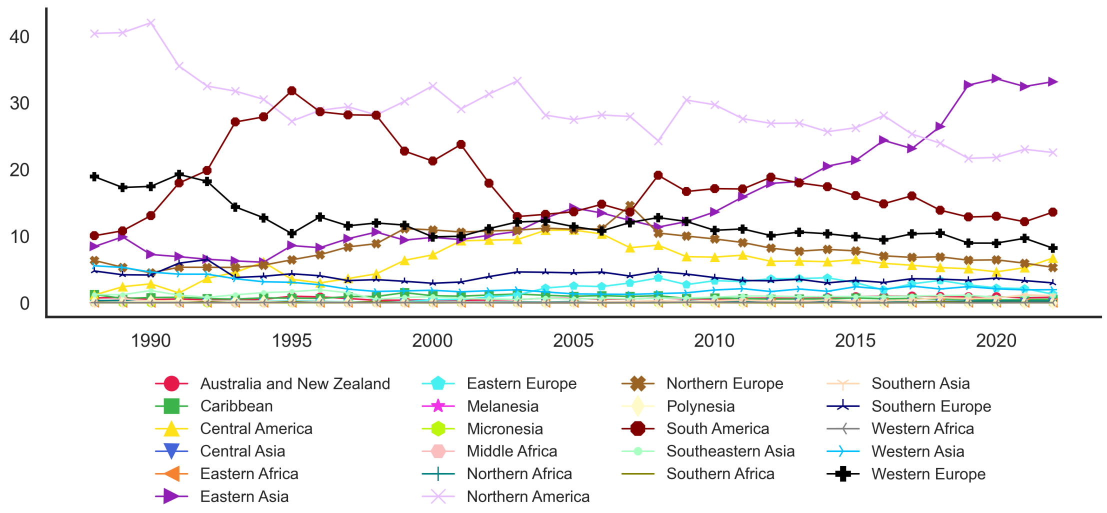

Figure S25. Evolving regional export shares [Percentage] of Chile (1988-2022).

Australia's agri-food export structure is globally engaged to a limited extent, with major flows to Eastern Asia (40%), Southeastern Asia (16.3%), and Northern America (11%). These regions provide high-income, stable markets with growing food demand, enhancing Australia's trade resilience. Secondary exports to Western Asia (6.7%) further diversify Australia's trade footprint. This distribution reflects both geographic proximity to Asia and strategic engagement with established global markets. However, heavy reliance on Eastern Asia poses geopolitical and market concentration risks. Trade tensions or policy shifts in the region could significantly impact Australia's agri-food sector. For policymakers, diversifying further into underutilized regions like Latin America and Africa, and strengthening trade with Europe, could reduce exposure to Asia-specific risks. According to the Figure S26, the dominance of Eastern Asia in Australia's export share is a striking feature, showing a consistent and often significant upward trajectory, particularly from the early 2000s, reflecting the deepening economic ties with major Asian economies. Conversely, exports to all regions of Europe have seen a general but slight decline in their share over the entire period. Trade with North America has also experienced a decrease in its overall share, especially in later years; similarly, Northern Africa has seen a slight overall decline. Meanwhile, Southeastern and Western Asia show a more mixed but

generally growing trend, suggesting their increasing importance as trade partners. Other regions, while individually smaller, have varying minor fluctuations over time.

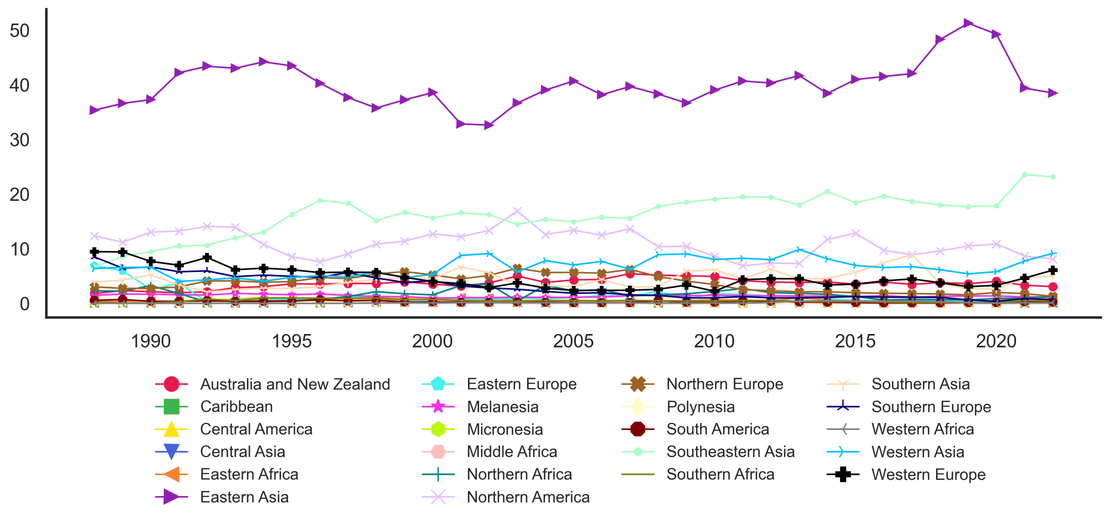

Figure S26. Evolving regional export shares [Percentage] of Australia (1988-2022).

France has a somewhat broader global export diversification relative to other major European exporters such as Germany, Spain, or Poland. While Western Europe still accounts for the largest single share (35.43%), France demonstrates broader engagement across non-European regions. Notably, Northern America (6.25%) and Eastern Asia (5.66%) are prominent extra-regional destinations. While France still sends around 73% of its exports to European subregions, including Southern Europe (21.92%) and Northern Europe (12.71%), its relatively higher global footprint suggests a more balanced position between regional orientation and global outreach. This approach partly offers greater resilience against regional shocks and enables better access to emerging market demand, compared to Germany, Spain, and Poland. Considering the Figure S27, France's export share to Western Europe illustrates a diminishing reliance on this traditional market over the years. Conversely, Eastern Asia and Northern America have also manifested a clear increase in their export share over time. Other regions usually have small, steady export shares with minor yearly variations.

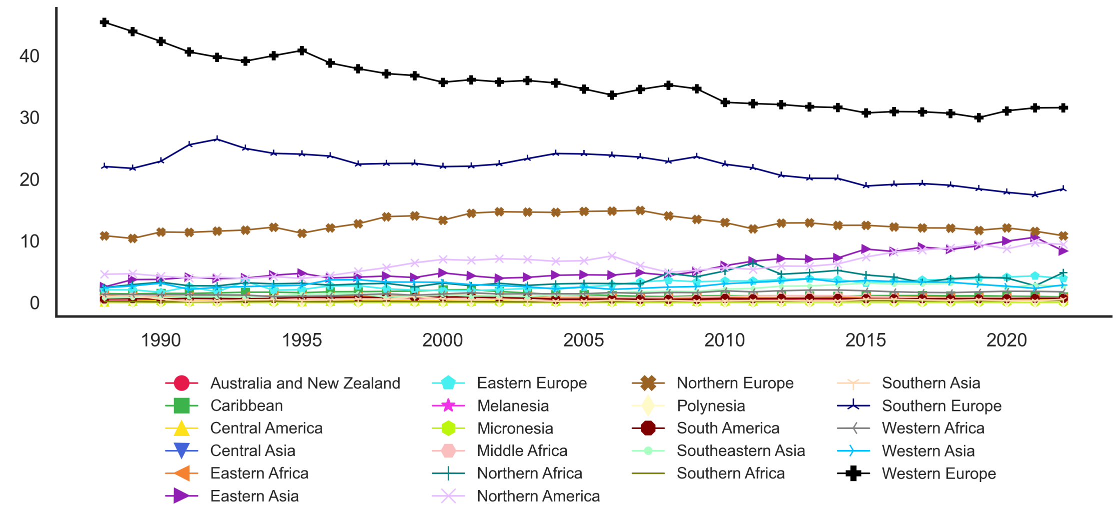

Figure S27. Evolving regional export shares [Percentage] of France (1988-2022).

From a resilience and food security perspective, this intra-European concentration among European nations may still pose systemic risks. For example, ongoing geopolitical instability in Eastern Europe, particularly due to the Russia–Ukraine conflict, can disrupt trade flows, impose logistical bottlenecks, and increase political uncertainty. Moreover, the lack of strong engagement with non-European markets may reduce these European countries' ability to absorb regional shocks or respond to shifting global food demand trends, especially in the context of climate change, global inflation, or supply chain disruptions. For policy-makers, this underscores the importance of strategically diversifying trade relations toward more global and structurally different economies. In the context of agri-food trade, such overdependence on regional buyers could also undermine food system resilience. If regional disruptions occur, such as disease outbreaks, protectionist policies, or climate-related harvest failures, they may struggle to reallocate their export flows quickly.

Countries with high export diversification (global exporters)

In contrast, some countries distribute their exports more evenly across multiple regions, exhibiting a strategic position as global exporters. For instance, Ukraine's agri-food export profile is characterized by a notably globalized and diversified composition.

The largest export shares are concentrated in Eastern Europe (24.36%), Western Asia (17.37%), Southern Asia (5.77%), Northern Africa (11.2%), Southern Europe (13.91%), Western Europe (10.96%), and the like. This distribution illustrates Ukraine's strong integration within its immediate European neighborhood, including Eastern Europe, Southern Europe, and Western Europe, collectively accounting for nearly half of its total exports and highlighting its role as a key agri-food supplier in a politically and economically interconnected region. Additionally, the substantial export share to Western Asia and Northern Africa underscores Ukraine's strategic outreach to markets beyond Europe, emphasizing both its geographic reach and commercial diversification. Given the complex geopolitical landscape in Eastern Europe, maintaining strong ties with Southern and Western Europe is likely to provide economic stability. However, the notable export reliance on Western Asia and Northern Africa introduces exposure to regions that have experienced periodic instability, including conflict zones and political upheaval, which could affect demand and increase Ukraine's vulnerability. Exports to Southern and Eastern Asia mark important emerging markets where growing populations and rising food demand present long-term opportunities. As depicted in the Figure S28, there is a rising trend in Ukraine's export shares to Eastern and Southern Asia.

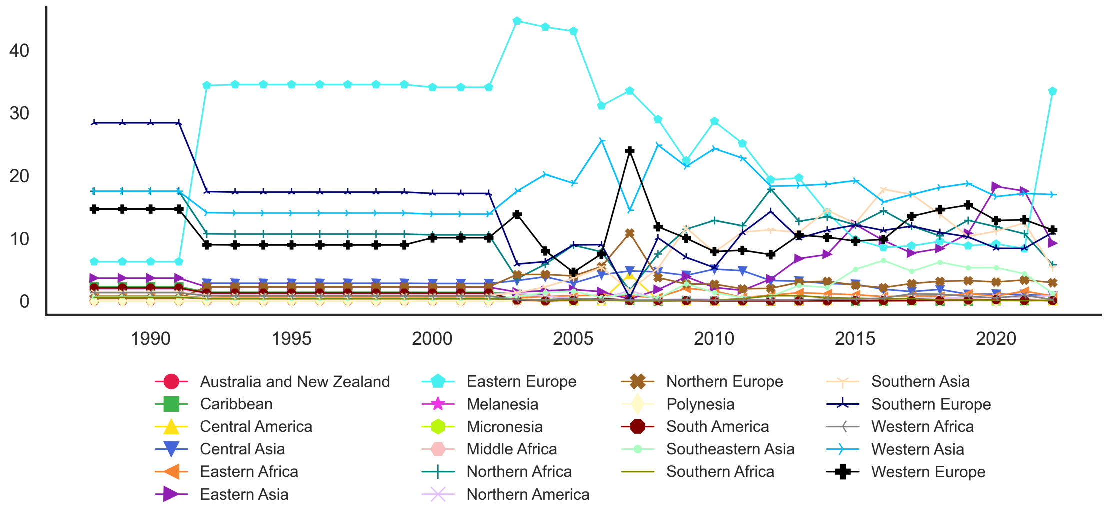

Figure S28. Evolving regional export shares [Percentage] of Ukraine (1988-2022).

Thailand's agri-food export structure is diversified. It includes Eastern Asia (34.1%),

Southeastern Asia (20.4%), Northern America (9.2%), Western Europe (7.4%), Western Africa (6.1%), and so on. This reflects Thailand's strategic location, robust production base, and active participation in global and regional trade networks. The dominance of Eastern and Southeastern Asia (together contributing over 54%) is consistent with Thailand's geographic proximity and deep integration within regional value chains. Its global participation provides a hedge against regional volatility. Thailand's export reach also spans Southern Asia (3.5%) and Southern Europe (2.0%). These smaller but stable markets present opportunities for deepening trade through targeted market entry strategies. Moreover, given some export concentration in neighboring and regional markets, continued diversification into Latin America, Sub-Saharan Africa, or Central Asia would enhance systemic resilience in the face of geopolitical uncertainty and global supply chain disruptions. The Figure S29 shows that Thailand's export shares to Eastern Asia and Northern America have increased despite some fluctuations over the course of time. Southeastern Asia also demonstrates strong, sustained growth, highlighting deeper regional trade integration. Meanwhile, exports to Western Europe and Southern Asia have declined. Other regions maintain small, stable export shares with minimal changes over time.

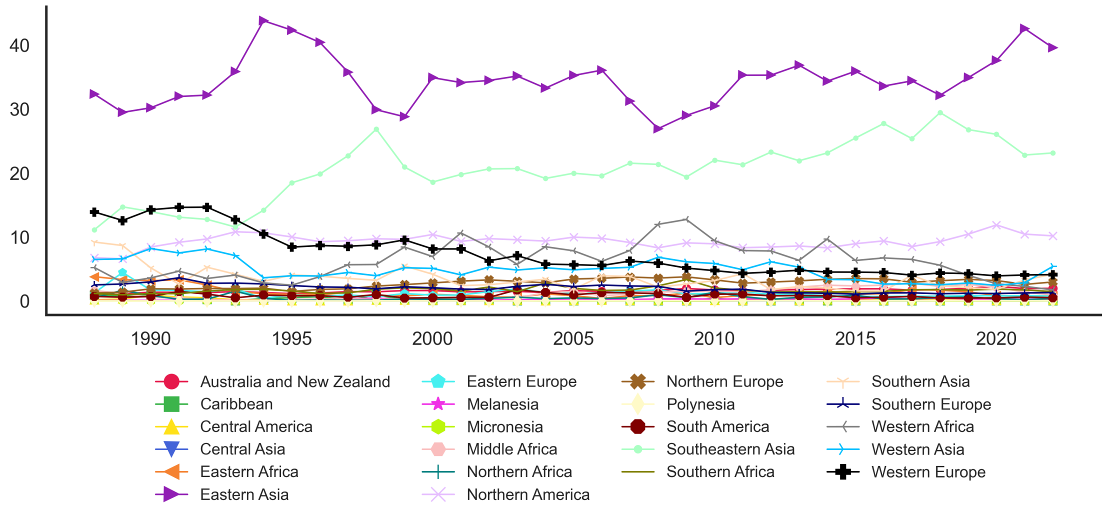

Figure S29. Evolving regional export shares [Percentage] of Thailand (1988-2022).

The United Kingdom's agri-food exports are both highly varied and extensively

linked to global markets. The largest shares of United Kingdom exports are sent toward Western Europe (28.88%), Northern Europe (19.74%), Southern Europe (12.28%), Northern America (10.07%), and Eastern Asia (7.46%), with additional notable flows to Western Asia (5.59%). This export distribution demonstrates the United Kingdom's strong commercial presence in high-income, politically stable regions. The United Kingdom's dominant export relationships with Western and Northern Europe underscore its deep integration within the European market, despite recent geopolitical shifts such as Brexit. These regional linkages provide the United Kingdom with a stable and lucrative market base, supporting resilience in its agri-food sector through access to consumers with high purchasing power and regulatory alignment. The significant export share to Southern Europe further diversifies this European portfolio, reducing the risk of over-dependence on any single sub-region. Exports to Northern America and Eastern Asia highlight the United Kingdom's ability to maintain robust transatlantic and Asia-Pacific connections, crucial for accessing large and growing consumer markets. However, these markets also present challenges, including tariff barriers, regulatory complexity, and geopolitical tensions that could affect trade flows. The United Kingdom's diversified export strategy enhances its resilience by spreading risk across stable and emerging markets, thereby mitigating vulnerabilities associated with economic downturns, trade disruptions, or geopolitical shocks in any single region. The Figure S30 shows limited changes in the UK's agricultural export profile during the 35 years. Notably, Western and Southern Europe's market share has significantly declined, while exports to Eastern Asia have steadily grown, highlighting its rising importance. Other regions maintain small, stable shares with minor fluctuations.

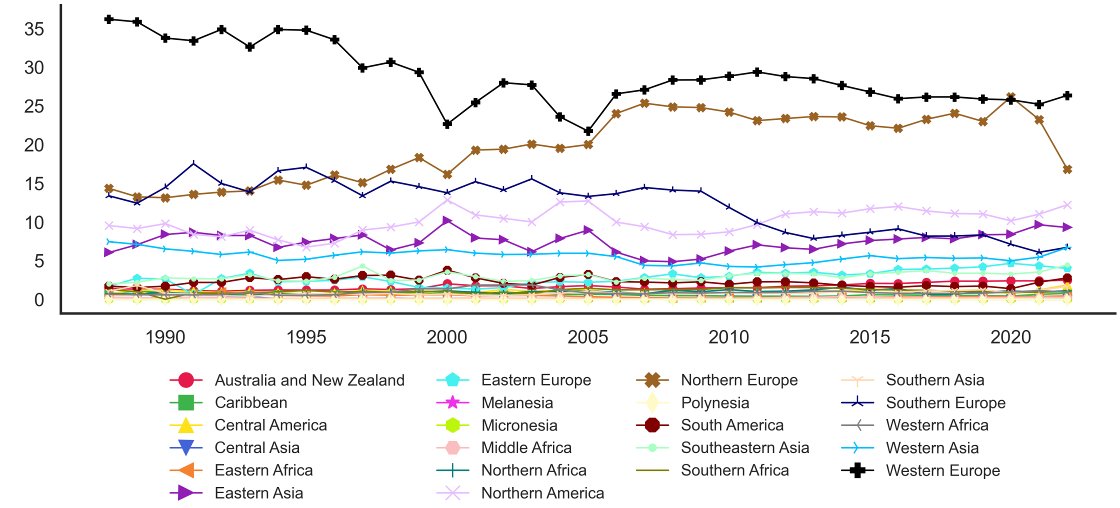

Figure S30. Evolving regional export shares [Percentage] of the United Kingdom (1988-2022).

Russia demonstrates an extensive agri-food export framework, with significant trade volumes dispersed across multiple regions, including Western Asia (22.17%), Central Asia (15.72%), Northern Africa (8.26%), Eastern Europe (9.7%), Southern Europe (8.5%), Northern Europe (6.11%), Western Europe (6.09%), and Micronesia (9.73%). This distribution highlights Russia's strategic position at the intersection of Europe and Asia, enabling it to access both traditional European markets and emerging Eurasian and Middle Eastern economies. The prominence of Western Asia and Central Asia can be partly attributed to Russia's geopolitical orientation. The substantial export shares to Northern and Southern Europe, as well as to Northern Africa, suggest active engagement with Mediterranean and EU-adjacent markets, considering these regions offer robust logistics channels. However, the relatively modest share directed toward Eastern Asia (5.21%), a region of high purchasing power and demand, indicates underexploited potential. From a resilience standpoint, Russia benefits from a balanced distribution across nearby (Western and Central Asia) and more stable, high-income markets (Europe). Nonetheless, the concentration in geopolitically sensitive regions, such as Western Asia and Central Asia, introduces vulnerability to sanctions, diplomatic frictions, and regional instability. Strengthening institutional trade frameworks beyond its immediate neighborhood, especially with Southeast Asia or Sub-Saharan Africa, would also improve strategic autonomy and mitigate geopolitical concentration risks. Regarding the Figure

S31, Russia's export profile demonstrates significant reconfigurations across the period. A clear trend is the noticeable decline in market shares for Western Europe, Micronesia, and Southern Europe, reflecting reduced dependence on these traditional markets. In contrast, exports to Eastern Asia have grown significantly and steadily, establishing it as a key export destination. Similarly, Northern Africa, Eastern Europe, Western Asia, and Southern Asia have shown growth, highlighting their increasing significance in Russia's agricultural trade network. Other regional destinations generally hold small, steady export shares with minimal changes over time.

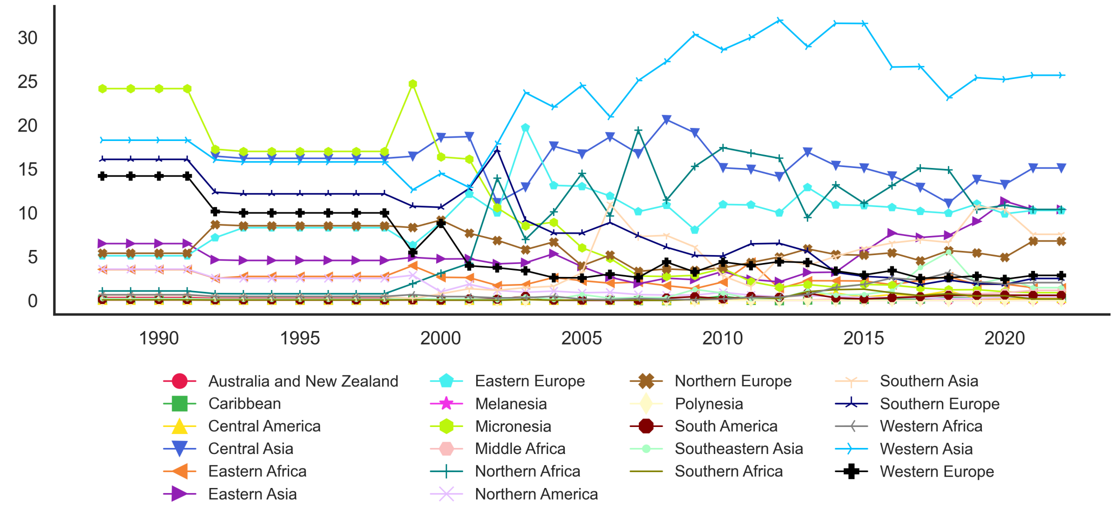

Figure S31. Evolving regional export shares [Percentage] of Russia (1988-2022).

Brazil demonstrates a well-diversified agri-food export profile, with major flows to Western Europe (22.42%), Eastern Asia (21.24%), Southern Europe (7.95%), and Northern America (8.4%). This balanced presence in both high-income Western markets and fast-growing Asian economies enhances trade resilience. Additional diversification toward Western Asia (6.92%), Eastern Europe (5.78%), and South America (5.5%) reflects Brazil's strong integration across multiple global regions. To further improve resilience, Brazil could expand underrepresented links in Africa and Southeast Asia while continuing to invest in trade diplomacy. According to the Figure S32, Eastern Asia shows a significant upward trend. South America also demonstrates a modest increase in its share, due to deepening regional trade integration. Conversely, exports to Western Europe have seen a

significant downward trend over the entire period. North America has also experienced a noticeable decrease in its overall share. Exports to Eastern, Southern, and Northern Europe have generally experienced a downturn. On the contrary, exports to Western, Southern, and Southeastern Asia have generally shown a notable increase. Other regions have small, stable export shares with slight fluctuations.

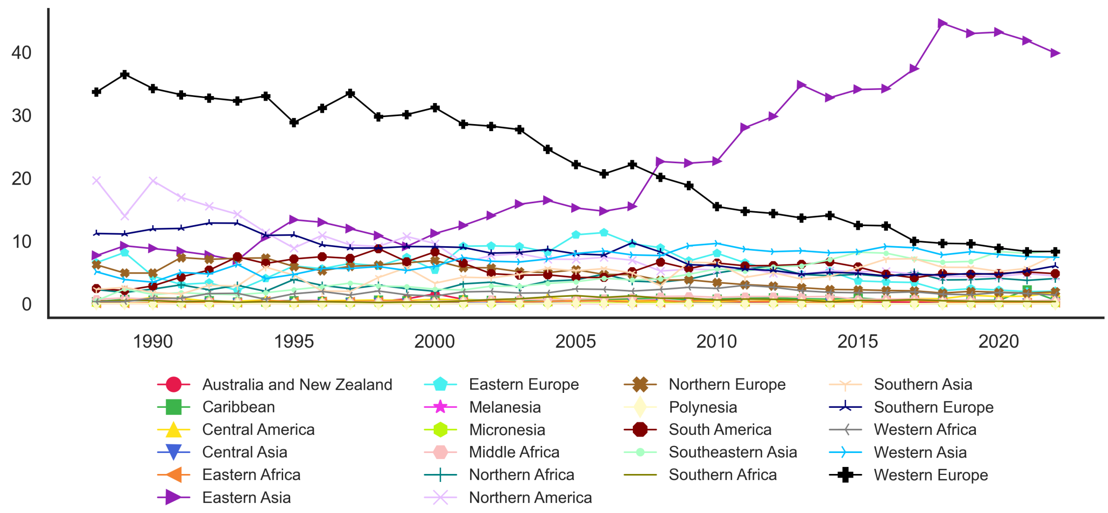

Figure S32. Evolving regional export shares [Percentage] of Brazil (1988-2022).

Malaysia also displays a highly diversified agri-food export structure, with major flows to Eastern Asia (22.97%), Southeastern Asia (20.92%), Southern Asia (14.84%), Western Europe (9.12%), Western Asia (6.63%), and Northern America (5.63%). This mix reflects Malaysia's strong integration within Asia, leveraging proximity and trade agreements, while maintaining valuable access to Western high-income markets. The balance between regional and global partners provides resilience to localized disruptions. However, significant reliance on East and Southeast Asia could create exposure to regional shocks or trade tensions. Policymakers should focus on expanding trade to Africa and Latin America. In the Figure S33 showing Malaysia's export share throughout the period, a discernible decline is evident in exports to North America and Western Europe, which points to a reduced reliance on these traditional markets. In contrast, Western Africa, Eastern Africa, Southern Asia, and Western Asia have all displayed a slight, gradual upward trend in their shares.

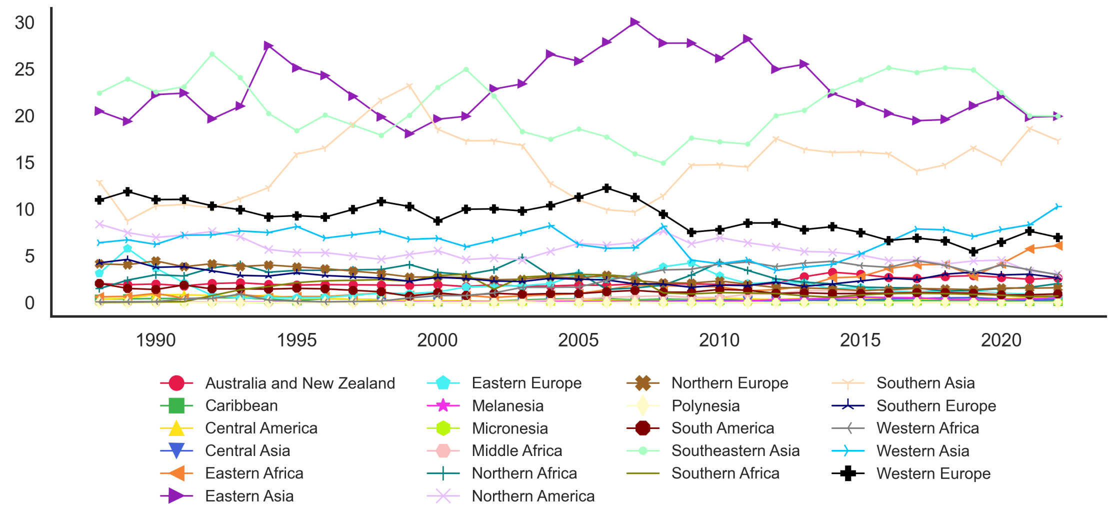

Figure S33. Evolving regional export shares [Percentage] of Malaysia (1988-2022).

India maintains a broad agri-food export footprint, with key destinations including Western Asia (22.67%), Southern Asia (13.48%), Southeastern Asia (13.34%), Eastern Europe (7.25%), Eastern Asia (8.8%), Northern America (8.86%), and Western Europe (7.59%). This diversified structure reflects India's strategic links across neighboring regions, emerging markets, and high-income economies. Strong ties with Western and Southern Asia benefit from logistical proximity, while growing engagement with Europe enhances market stability. However, high reliance on politically volatile regions like Western Asia may pose risks. To further strengthen resilience, India should deepen partnerships with African and Latin American markets. Taking into account the Figure S34, India's export shares demonstrate significant shifts over the period. A gradual upward growth is observed in exports to Western and Eastern Africa. Concurrently, Southern and Southeastern Asia also exhibit a marked and sustained increase in their respective shares, reflecting India's growing trade integration with the Asian continent. Conversely, a discernible downturn is evident in the export shares directed towards Western, Northern, Southern, and considerably Eastern Europe, signaling a decreasing reliance on this continent. Other regions generally have small, stable export shares with minimal variation over time.

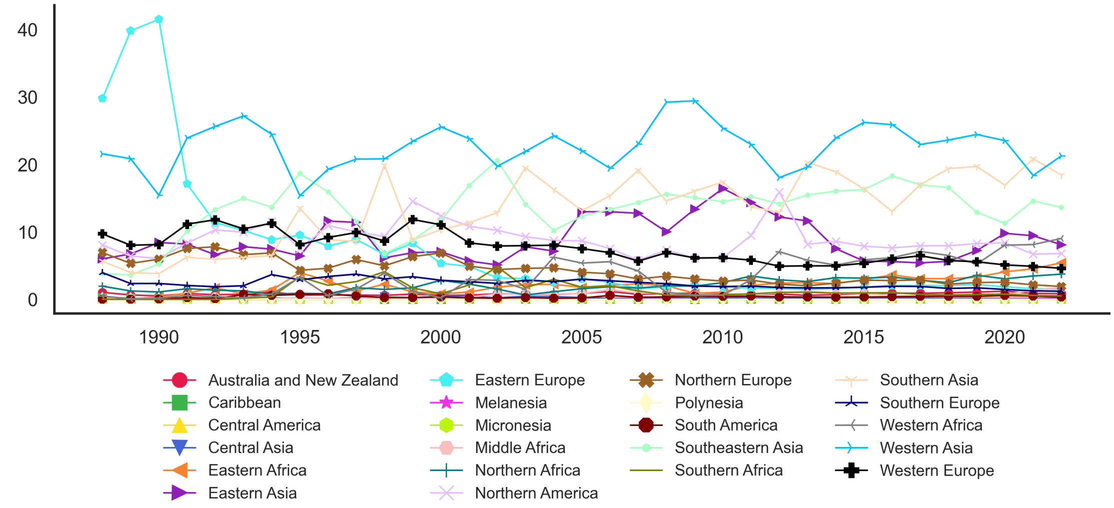

Figure S34. Evolving regional export shares [Percentage] of India (1988-2022).

Argentina displays a highly diversified agri-food export profile, with significant shares in South America (22.72%), Western Europe (13.23%), Eastern Asia (11.64%), Southeastern Asia (7.7%), Southern Europe (7.73%), Southern Asia (7.11%), Northern Africa (6.27%), Western Asia (4.85%), and Northern America (4.83%). This broad geographic spread reduces reliance on any single market, enhancing resilience against regional economic or political shocks. Strong ties within South America leverage geographic proximity, while access to Europe and Asia taps into high-value and rapidly growing markets. According to Figure S35, the prominence of South America often shows strong co-movements and a generally upward trend in export share, especially in the 1990s following Mercosur's establishment. Asia's growing importance is also a key feature, particularly Eastern, Southern, Western, and Southeastern Asia, which shows a significant upward trajectory in export share from the late 1990s onwards. This reflects the broader shift in Argentina's increasing engagement with fast-growing Asian economies, driven by demand for its agricultural commodities. Its agricultural trade with the EU, especially Western, Southern, and Eastern Europe, has generally declined. Meanwhile, trade with North Africa has grown somewhat, and trade with North America has decreased slightly.

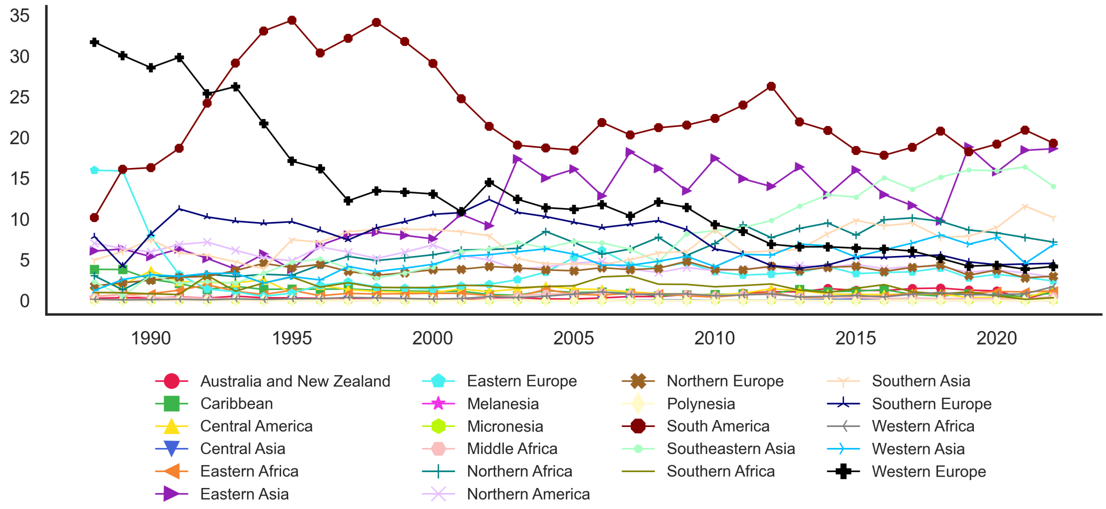

Figure S35. Evolving regional export shares [Percentage] of Argentina (1988-2022).

Discussion

The primary limitation of this study arises from data availability issues, which may constrain the depth and scope of analysis. Indeed, the remaining exporters were excluded due to incomplete and inconsistent data coverage, as their inclusion would have required substantial imputation and could materially bias the results. However, our data sources are well-accepted organizational platforms that are constantly being regulated and updated. Thus, our findings have a solid foundation and a significant importance to initiate better-informed trade strategies for both exporter and importer nations around the globe. Secondly, we acknowledge that export market shares may vary due to factors outside a country's direct control, including exchange-rate movements, commodity price cycles, and supply disruptions in competing exporters. Accordingly, export market share should not be interpreted as a structural measure of comparative advantage, but rather as a revealed, aggregate outcome of trade dynamics over time. Thirdly, this analysis treats agri-food exports as an aggregate category and does not distinguish between individual product segments. Accordingly, the results should be interpreted as reflecting aggregate export trajectories rather than product-specific performance. This aggregation is primarily driven by data availability and comparability constraints, as

Table S40. Shannon diversity index averaged across 1988–2022 for the 25 agri-food exporting countries. This table ranks countries based on the average Shannon entropy of their agri-food export market shares across global regions. Higher values indicate greater historical diversity in export destinations, reflecting a more balanced and diversified export structure.

Country	Shannon Diversity Index
Argentina	3.3987
India	3.2777
Malaysia	3.2609
Brazil	3.2561
Russia	3.2106
United Kingdom	3.1188
Thailand	3.0061
Ukraine	2.9854
France	2.9015
Australia	2.8693
Chile	2.8630
Finland	2.7630
China	2.7586
Portugal	2.7275
Philippines	2.5919
Germany	2.5835
Spain	2.5361
Italy	2.5132
Poland	2.5035
Tunisia	2.4706
Canada	2.4105
Netherlands	2.4035
Belgium	2.0164
United States	1.8207
Mexico	1.2588

consistent product-level determinants are not uniformly reported across countries and over long time horizons. Moreover, different agri-food products exhibit heterogeneous market structures and require distinct explanatory variables, making large-scale product-level forecasting unreliable under current data limitations. Nonetheless, differences in export composition may influence aggregate trajectories. More specifically, agri-food exports encompass heterogeneous product segments that differ in value added, volatility, demand elasticity, and exposure to policy and logistical constraints. Countries specializing in higher value-added or processed products may exhibit more stable and persistent export growth, while exporters concentrated in primary commodities may experience greater sensitivity to price cycles and supply shocks. As a result, shifts in export composition can influence both the level and dynamics of aggregate export performance. Future estimates produced by our two-stage forecasting framework are best interpreted as conditional, scenario-based projections. Indeed, the approach assumes that historical associations

Table S41. Regional classification of 25 selected countries. This categorization is based on the definitions of the Food and Agriculture Organization of the United Nations (FAO) [109].

Country	Region
Argentina	South America
Australia	Australia and New Zealand
Belgium	Western Europe
Brazil	South America
Canada	Northern America
Chile	South America
China	Eastern Asia
Finland	Northern Europe
France	Western Europe
Germany	Western Europe
India	Southern Asia
Italy	Southern Europe
Malaysia	Southeastern Asia
Mexico	Central America
Netherlands	Western Europe
Philippines	Southeastern Asia
Poland	Eastern Europe
Portugal	Southern Europe
Russia	Eastern Europe
Spain	Southern Europe
Thailand	Southeastern Asia
Tunisia	Northern Africa
Ukraine	Eastern Europe
United Kingdom	Northern Europe
United States	Northern America

between export shares and their explanatory variables observed during 1988–2022 remain broadly informative over the projection horizon. Given the possibility of structural breaks arising from geopolitical events, climate shocks, policy regime changes, or structural transformations in food systems, these projections should be interpreted as indicative of the prospective positioning of leading exporters, rather than as deterministic or precise point forecasts. Similarly, we do not report formal prediction intervals because the forecasting pipeline is explicitly two-stage and nonlinear; constructing calibrated intervals would require careful propagation of uncertainty from multiple predictor forecasts through the export-share mapping. Reporting naïve or nominal intervals in the absence of such propagation could therefore be misleading. Finally, Value-based measures may be influenced by commodity composition, price cycles, and unit-value differences. While these effects are inherent to macro trade data, they are directly relevant for policy discussions focused on trade revenue and economic exposure rather than tonnage alone.

SI References

[1] World Bank. 2025. Worldwide Governance Indicators. Online database. World Bank Group. Accessed date: 8 April 2025.

[2] World Bank. 2025. Logistics Performance Index (LPI): International LPI. Online interactive data tool. World Bank Group. Accessed date: 8 April 2025.

[3] World Bank. 2025. Prosperity Data 360: Global Investment and Innovation Indicators. Online data dashboard. World Bank Group. Accessed date: 10 April 2025. Indicators: GIH, GIO, 24.

[4] Organisation for Economic Co-operation and Development (OECD). 2025. OECD Data Explorer: Infrastructure Maintenance and Investment Indicators. Online interactive data tool. OECD. Accessed date: 10 April 2025.

[5] Organisation for Economic Co-operation and Development (OECD). 2025. OECD Data Explorer: Infrastructure Maintenance and Investment. Online interactive data tool. OECD. Accessed date: 1 January 2025. Indicators: MNT (Maintenance), INV (Investment).

[6] World Bank. 2025. GDP (current US$): World Bank Open Data. World Bank Group. Accessed date: 8 April 2025.

[7] World Bank. 2025. World Bank Data Tables. Online data tables. World Bank Group. Accessed date: 9 April 2025.

[8] USDA Economic Research Service. 2023. International Agricultural Productivity. Online data product. USDA Economic Research Service. Accessed date: 12 April 2025. Data product: International Agricultural Productivity.

[9] Caldara, Dario, and Matteo Iacoviello. 2022. Measuring Geopolitical Risk. *American Economic Review*, 112(4): 1194–1225. American Economic Association.

[10] Caldara, Dario, and Matteo Iacoviello. 2024. Geopolitical Risk Index Data. Data version: December 2024.

[11] Centre for Research on the Epidemiology of Disasters (CRED). 2024. EM-DAT Disaster Event Records. Accessed date: 1 December 2024.

[12] Food and Agriculture Organization of the United Nations (FAO). 2025. FAOSTAT: Food and Agriculture Organization Statistical Database. FAO. Accessed date: 8 April 2025.

[13] Little, Roderick J. A., and Donald B. Rubin. 2019. *Statistical Analysis with Missing Data*. John Wiley & Sons.

[14] Field, Andy. 2024. *Discovering Statistics Using IBM SPSS Statistics*. Sage Publications Limited.

[15] Challu, Cristian, Kin G. Olivares, Boris N. Oreshkin, Federico Garza Ramirez, Max Mergenthaler Canseco, and Artur Dubrawski. 2023. N-HiTS: Neural Hierarchical Interpolation for Time Series Forecasting. In *Proceedings of the AAAI Conference on Artificial Intelligence*, 37(6): 6989–6997. Association for the Advancement of Artificial Intelligence, Washington, DC, USA.

[16] James, Gareth, Daniela Witten, Trevor Hastie, and Robert Tibshirani. 2013. *An Introduction to Statistical Learning*. 1st ed. Springer Texts in Statistics, Vol. 112(1). Springer, New York. With applications in R.

[17] Food and Agriculture Organization of the United Nations (FAO). 2024. FAOSTAT: Food and Agriculture Data. FAO. Accessed date: 26 April 2025.

[18] Breiman, Leo. 2001. Random Forests. *Machine Learning*, 45(1): 5–32. Springer.

[19] Biau, Gérard. 2012. Analysis of a random forests model. *The Journal of Machine Learning Research*, 13(1): 1063–1095.

[20] Mann, Henry B. 1945. Nonparametric Tests Against Trend. *Econometrica*, 13(3): 245–259. The Econometric Society.

[21] OECD and FAO. 2024. OECD-FAO Agricultural Outlook 2024-2033. OECD Publishing, Paris/FAO, Rome. Accessed date: 28 July 2025.

[22] Bojnec, Štefan, and Imre Fertő. 2019. European Union countries agri-food trade structures and main competitors on the internal and global agri-food markets. *Ekonomika poljoprivrede*, 66(2): 635–650. Accessed date: July 29, 2025.

[23] ASEAN Connectivity Coordinating Committee. 2020. Framework for Improving ASEAN Infrastructure Productivity. Technical report. ASEAN Secretariat, Jakarta, Indonesia. Published by the ASEAN Secretariat. Accessed date: 25 July 2025.

[24] Transport Canada. 2019. Transportation 2030: Trade Corridors to Global Markets. Strategic plan. Government of Canada. Part of Canada's Transportation 2030 initiative. Accessed date: 25 July 2025.

[25] International Trade Administration. 2024. Chile Infrastructure Opportunities. Market intelligence report. U.S. Department of Commerce. Accessed date: 26 July 2025.

[26] The State Council of the People's Republic of China. 2025. China Unveils New Infrastructure Investment Plan to Boost Economic Growth. *Gov.cn English*. The State Council of the People's Republic of China. Accessed date: 25 July 2025.

[27] European Investment Bank. 2024. Tunisia: Strategic corridor - EIB World invests €210 million with EU support for the strategic modernisation of the Sfax-Kasserine road corridor. Press release. European Investment Bank. Accessed date: 26 July 2025.

[28] Kumar, Santosh, Sarla Meena, and Rishita Aggarwal. 2025. Transforming India's Transport Infrastructure (2014–2025). Press Information Bureau, Government of India. Accessed date: 26 July 2025.

[29] International Trade Administration. 2024. Argentina Transportation Infrastructure Sector. Market intelligence report. U.S. Department of Commerce. Accessed date: 26 July 2025.

[30] Global Infrastructure Hub. n.d. Mexico Country Profile: Infrastructure Policy Assessment. Country benchmarking report. Global Infrastructure Hub. Accessed date: 26 July 2025.

[31] Global Infrastructure Hub. n.d. Russia Country Profile: Infrastructure Policy Assessment. Country benchmarking report. Global Infrastructure Hub. Accessed date: 25 July 2025.

[32] International Trade Administration (U.S. Department of Commerce). 2023. Ukraine - Infrastructure. Country Commercial Guide. U.S. Department of Commerce. Accessed date: 26 July 2025.

[33] Keep, Matthew, Georgina Hutton, and Sarah Lewis. 2025. Infrastructure in the UK. Research Briefing No. SN06594. House of Commons Library. Accessed date: 25 July 2025.
